# Direct characterisation of *m*_*J*_ = ±15/2 ground state in octahedral Dy(iii) single-molecule magnets†

**DOI:** 10.1039/d5dt00862j

**Published:** 2025-04-17

**Authors:** Vijay S. Parmar, Gemma K. Gransbury, Sophie C. Corner, Wei-Hao Chou, Stephen Hill, Richard E. P. Winpenny, Nicholas F. Chilton, David P. Mills

**Affiliations:** a Department of Chemistry, School of Natural Sciences, The University of Manchester Oxford Road Manchester M13 9PL UK david.mills@manchester.ac.uk; b National High Magnetic Field Laboratory, Florida State University Tallahassee FL 32310 USA; c Department of Physics, Florida State University Tallahassee FL 32306 USA; d Department of Chemistry and Biochemistry, Florida State University Tallahassee FL 32306 USA; e Research School of Chemistry, The Australian National University Canberra ACT 2601 Australia nicholas.chilton@anu.edu.au

## Abstract

Two heteroleptic octahedral Dy(iii) *cis*-aryloxide complexes, [Dy(OPh*)_2_(THF)_3_X] {HOPh* = 2,6-bis(diphenyl-methyl)-4-*tert*-butylphenol; X = Cl (1), Br (2)}, have been characterised by multi-frequency electron paramagnetic resonance (EPR) spectroscopy to determine *g*_*z*_ = 18.9(1) for 1 and 18.3(6) for 2. These are rare examples of Dy(iii) single-molecule magnets that have observable EPR spectra.

## Abstract 2

The magnetic ground states of two dysprosium bis-aryloxide single-molecule magnets are probed by EPR spectroscopy, magnetometry and *ab initio* calculations.

## Graphical abstract

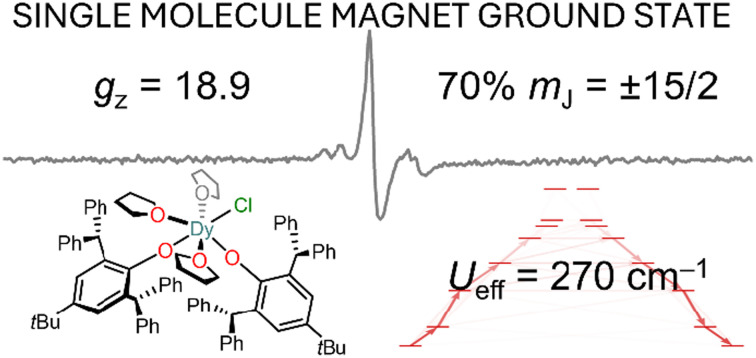

Molecules that show slow relaxation of magnetisation without long range ordering are known as single-molecule magnets (SMMs).^1,2^ SMMs could offer applications in ultra-high density data storage, molecular spintronics and quantum computing.^3^ Most recent examples of high-performing mononuclear lanthanide (Ln) SMMs contain a single Dy(iii) centre with anionic ligands on a unique axis and weak neutral ligands (or no ligands) in the equatorial plane.^4^ This arrangement constructs an energy barrier to magnetic reversal (*U*_eff_) by stabilising the largest, and destabilising the smallest, *m*_*J*_ projections for a given total angular momentum *J*.^1,2^ Thus, the ideal ground state for a Dy(iii) SMM is the *m*_*J*_ = ±15/2 state of its lowest lying ^6^H_15/2_ term. Modern characterisation of Ln SMMs, alongside traditional experimental magnetic measurements, has gravitated towards placing key significance on multiconfigurational quantum chemistry methods that enable *ab initio* prediction of molecular electronic structure and magnetic anisotropy. The explosion in popularity of these methods is in a large part because of the paucity of direct experimental methods available to characterise the *m*_*J*_ composition of the ground state in SMMs. While electron paramagnetic resonance (EPR) spectroscopy has been a long-standing technique to perform exactly this experiment, the properties of pure-*m*_*J*_ states in easy-axis magnetic anisotropy are such that EPR transitions within the ground state are forbidden by the selection rule Δ*m*_*J*_ = ±1 (*e.g.* Δ*m*_*J*_ = ±15 for the *m*_*J*_ = ±15/2 doublet). Another way to view this is that the *m*_*J*_ = ±15/2 doublet behaves as an effective *S*_eff_ = 1/2 with *g*_*z*_ = 20 and *g*_*x*_ = *g*_*y*_ = 0, and the vanishing transverse *g*-values means the state is EPR silent.^5^ In fact, given the propensity for monometallic Dy(iii) complexes to function as SMMs,^6,7^ and hence have ground states dominated by *m*_*J*_ = ±15/2, EPR spectra of such complexes are rare,^8–11^ and the maximum observed *g*_*z*_ is 15.5 for a diluted Dy(iii) aza-annulide SMM with a dominant *m*_*J*_ = ±13/2 ground state;^9^ although *g*_*z*_ = 19.55(16) has been measured for the inorganic salt [Dy(PO_4_)].^12^ As the mixing between the different *m*_*J*_ functions increases (and the SMM properties diminish), the EPR transition probability generally increases as components of the doublet having Δ*m*_*J*_ = ±1 increase, so that increases in *g*_*x*_ and *g*_*y*_ are observed along with a decrease in *g*_*z*_. However, a measured *g*_*z*_ value above the values for a given pure *m*_*J*_ state (Table 1) is a reporter for the dominant *m*_*J*_ component of the ground doublet: for example, the observation of *g*_*z*_ = 15.5 (being greater than the maximal value for *m*_*J*_ = ±11/2 of 14.67) implies significant *m*_*J*_ = ±13/2 components,^9^ and so observation of *g*_*z*_ > 17.33 would indicate a significant *m*_*J*_ = ±15/2 component.

**Table 1:** Effective *g*_*z*_ values for pure *m*_*J*_ states of Dy(iii)

*m*_*J*_	±15/2	±13/2	±11/2	±9/2	±7/2	±5/2	±3/2
*g*_*z*_	20.00	17.33	14.67	12.00	9.33	6.67	4.00

Many Ln(iii) alkoxide and aryloxide SMMs have been reported because of the wide array of ligand substituents that allows for tuning of the crystal field (CF) and resulting Ln(iii) electronic structure.^13^ There are many mononuclear Dy(iii) alk-/aryl-oxide SMMs with a coordination number of seven,^13^ but only a handful of six-coordinate examples reported to date, most of which contain two anionic ligands mutually *trans*- to one another and four neutral equatorial ligands.^13–21^ Here we report the synthesis and structural authentication of two six-coordinate Ln(iii) aryloxide complexes [Ln(OPh*)_2_(THF)_3_X] (HOPh* = 2,6-bis(diphenyl-methyl)-4-*tert*-butylphenol; Ln = Dy, X = Cl (1), Br (2)) with *cis* aryloxides. These compounds are SMMs and have EPR-active ground doublets allowing us to measure ground-state *g*_*z*_ values of 18.9(1) and 18.3(6) for complexes 1 and 2, respectively, presenting unambiguous experimental evidence of *m*_*J*_ = ±15/2 ground states.

Complexes 1 and 2 (along with the Y(iii) analogue of 1 (Ln = Y, X = Cl, 1-Y), and a doped ∼5% Dy(iii) in Y(iii) sample (Ln = Dy_0.05_Y_0.95_, X = Cl, 5%Dy@1-Y)) were prepared by reacting two equivalents of a group 1 metal salt of the aryloxide ligand, MOPh* (M = Na, K),^22^ with anhydrous DyX_3_ (X = Cl (1), Br (2)), YCl_3_ (1-Y) or a 1 : 19 ratio of DyCl_3_ and YCl_3_ (5%Dy@1-Y) in THF under reflux. The products were purified by filtration and recrystallisation in *ca*. 50% yields; elemental analysis, NMR and IR spectroscopy were performed to characterise all products, and these are compiled in the ESI together with full experimental details. The ^1^H and ^13^C NMR spectra of diamagnetic 1-Y are consistent with structural data obtained from a single crystal, providing additional confidence to the bulk formulations of 1, 2 and 5%Dy@1-Y.

The solid-state structures of 1, 1-Y, 5%Dy@1-Y and 2 were determined by single crystal XRD (Fig. 1 and ESI Fig. S11–S13 and Table S1†). All structures crystallize in the *P*1̄ space group with a single crystallographically unique metal complex. The co-crystallised lattice solvent varies: 2 Et_2_O (1), 3 THF (1-Y), 1 pentane (5%Dy@1-Y) and 1.5 *n*-hexane (2).

**Fig. 1:**
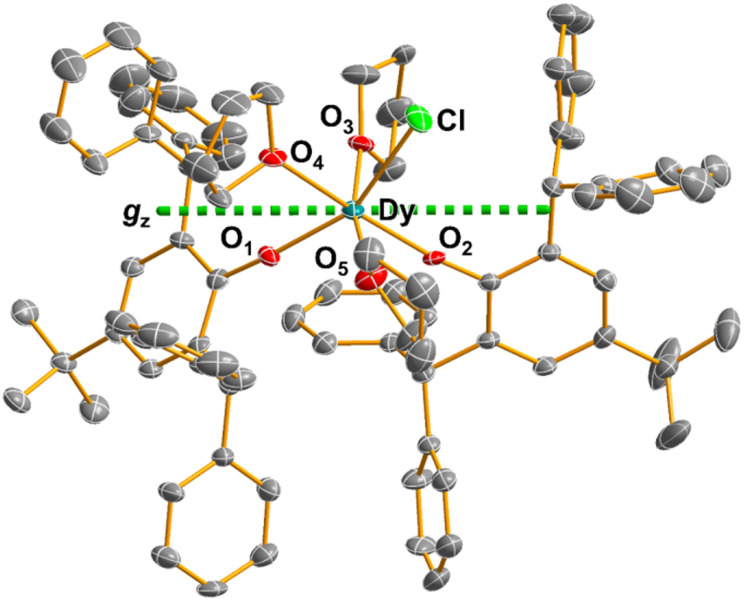
Molecular structure of 1 from single crystal XRD at 150 K with thermal ellipsoids drawn at 40% probability level and selective atom labelling (Dy teal, Cl green, O red, C grey). Dotted green line represents the calculated principal magnetic axis for 1. H atoms and lattice solvent are omitted for clarity.

All complexes exhibit distorted octahedral geometries, as confirmed by Shape2.0^23^ (shape index of 2.10, 2.21, 1.19 and 1.16 for 1, 2, 1-Y and 5%Dy@1-Y, respectively) (Table S2, and Fig. S14†). The Y/Dy(iii) coordination spheres of 1, 1-Y, 5%Dy@1-Y and 2 show a meridional arrangement of the three THF ligands, with the two aryloxide ligands *cis*- to each other, and one of them *trans*- to the halide. Selected bond distances in 1, 1-Y, 5%Dy@1-Y and 2 are given in Table S3.† The Dy–O_Ph*_ distances *trans*- to the Dy–X bonds (2.141(3) Å for 1 and 2.130(3) Å for 2) are longer than those that are *cis*- to the halides (2.109(4) Å for 1 and 2.106(3) Å for 2). As expected from electrostatic arguments, the Ln–O_Ph*_ distances are shorter than the Ln–O_THF_ distances. The Dy–X distances are 2.6462(14) (1) and 2.8301(5) Å (2), as expected from variation of the halide. The O_Ph*_–Dy–O_Ph*_ angles are 108.16(14)° for 1, and 105.86(10)° for 2. The nearest intermolecular Dy⋯Dy distances are 8.795 Å (1) and 11.303 Å (2; see Fig. S15–S23† for depictions of crystal packing).

*Ab initio* complete active space self-consistent field spin–orbit (CASSCF-SO) calculations were performed on 1, 2 and 5%Dy@1-Y (details in ESI†) and predict ground states (GS) with *ca.* 70% *m*_*J*_ = ±15/2 and 25% ±11/2 compositions for 1 and 2, with *g*_*z*_ values of 18.12 and 17.95 (0.2 < *g*_*x*,*y*_ < 1), respectively (Table S4†). The GS of 5%Dy@1-Y is slightly more axial with *ca.* 80% *m*_*J*_ = ±15/2 and 20% ±11/2, *g*_*z*_ = 18.53 and 0.1 < *g*_*x*,*y*_ < 0.5. The short Dy–O_Ph*_ bonds dictate the principal magnetic axes for 1, 2 and 5%Dy@1-Y (Fig. 1, S24–S26†), similar to that recently observed for a two-coordinate bis-amide Dy(iii) SMM.^24^

The CASSCF-SO calculation predicts a mixed ground state, which led us to study the EPR spectroscopy of 1 and 2. Continuous wave (CW) X-band (*ν* ∼ 9.4 GHz) EPR spectra of solid samples 1 and 2 at 6.9 K show a sharp feature at low field and a much broader feature at around 0.75 T (Fig. 2 and S30†). Simulation with a *S*_eff_ = ½ model gives *g*_*z*_ = 18.6, *g*_*y*_ = 0.83 (1) and *g*_*z*_ = 18.9, *g*_*y*_ = 0.82 (2), with *g*_*x*_ < 0.4 not observed in 1 nor 2 (Table S7†), in good agreement with CASSCF predictions. Frozen solution spectra of 1 and 2 (10 mM in 9 : 1 toluene : hexane, 5 K) show sharper resonances for the *g*_*z*_ feature but the broad feature for *g*_*y*_ broadens further still. The hyperfine coupling to ^161^Dy and ^163^Dy (both *I* = 5/2) is resolved on the *g*_*z*_ feature (Fig. S32 and S33†).

**Fig. 2:**
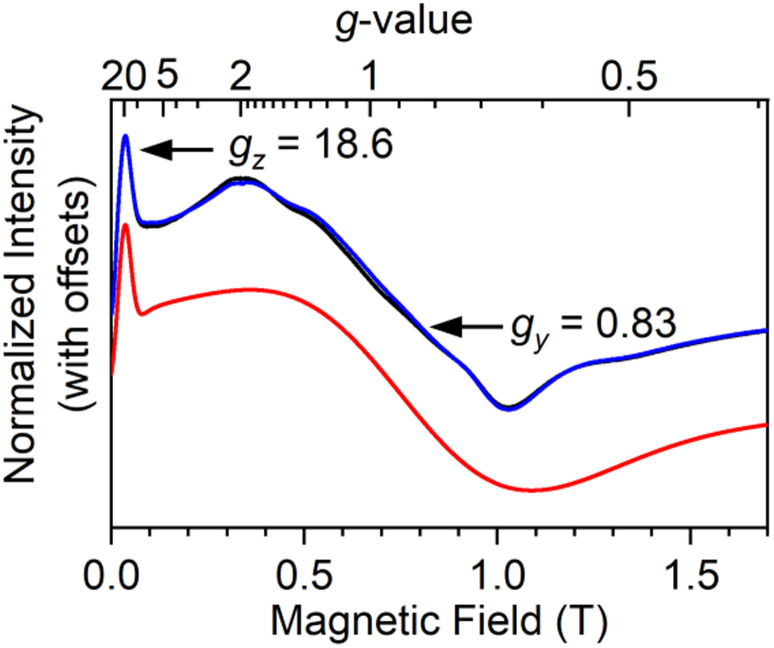
Continuous wave X-band (*ν* = 9.379332 and 9.380314 GHz) EPR powder spectra of 1 at 6.9 K showing two experimental spectra at approximately perpendicular sample rotations (black, blue) and simulation (red).

The EPR spectrum was also measured on a solid-state doped material 5%Dy@1-Y. The X-band spectrum of 5%Dy@1-Y has a sharper *g*_*z*_ feature at 19.44 and a broader but clearly visible derivative feature around *g*_*y*_ = 0.5 (Fig. S34 and Table S7†). The differences of *g*_*z*_ for 1 between 18.6 (pure solid), 18.45 (frozen solution) and 19.44 (doped solid) are within experimental uncertainty as large *g*-values move quickly with magnetic field at X-band, and these differences are only 1.5 mT (Fig. S35†), which compares with line widths of ∼4.4 mT (frozen solution and doped solid) and field calibrations of 1.7–3.3 mT (applied at *g* = 2.0028).

To confirm the *g*_*z*_ value, we measured high field and high frequency (HF-)EPR on 5%Dy@1-Y at 128 and 257.2 GHz (Fig. 3, S36 and Table S7†). A sharp *g*_*z*_ feature (peak-to-peak line width 4.4 mT) with Dy hyperfine is observed at *g*_*z*_ = 18.80 (128 GHz) or *g*_*z*_ = 19.00 (257.2 GHz). The Dy hyperfine coupling constants of 141 MHz (frozen-solution 1, X-band), 139 MHz (frozen solution 2, X-band) and 140 MHz (5%Dy@1-Y, 257.2 GHz) are comparable to the value of 213 MHz previously reported for Dy@C_81_N.^8^ Pulsed X-band EPR was attempted but no echo was observed for 5%Dy@1-Y, powder 1 or a 10 mM frozen solution of 1.

**Fig. 3:**
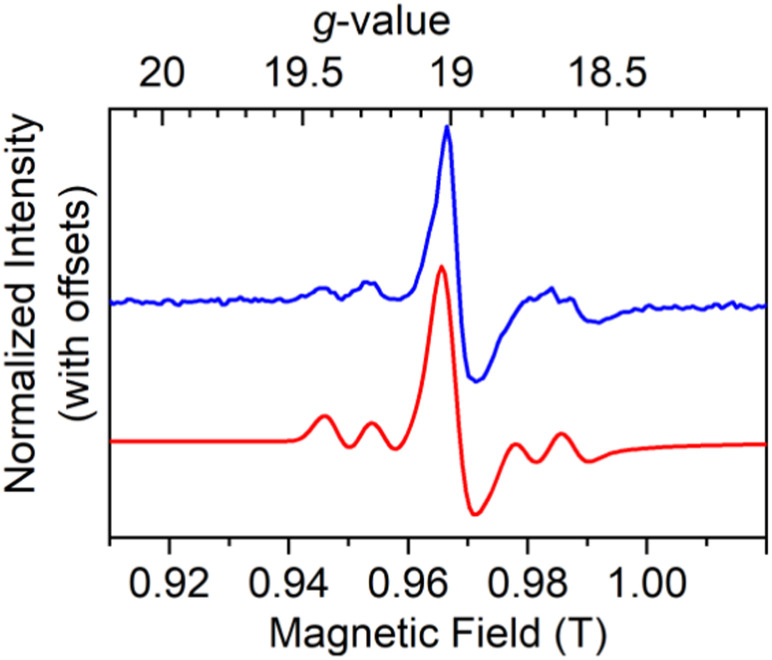
High-field EPR powder spectra of 5%Dy@1-Y at 257.2 GHz showing experimental spectra at 5 K (blue) and simulation (red).

Following CASSCF-SO prediction of significant *m*_*J*_ = ±15/2 contributions to the GS of 1 and 2 and subsequent confirmation with EPR spectroscopy, we performed magnetic measurements to probe their SMM characteristics. Static (direct current, dc) magnetic measurements on immobilised polycrystalline samples of 1 and 2 reveal molar *χ*_m_*T*_300 K_ values of 12.90 (1) and 12.87 cm^3^ K mol^−1^ (2; Fig. S37†) that are slightly lower than expected for Dy(iii) (free ion: ^6^H_15/2_, *χ*_m_*T* = 14.17 cm^3^ K mol^−1^).^14,15,25^ The *χ*_m_*T* products reduce with temperature and start to decline faster below *ca.* 50 K, reaching *χ*_m_*T* = 10.46 (1) and 9.67 cm^3^ K mol^−1^ (2) at 2 K. The magnetisation at 2 K and 7 T saturates at *M*_sat_ = 4.74 (1) and 4.84 *N*_A_*μ*_B_ (2; Fig. S38 and S39†). Magnetic hysteresis curves for 1 and 2 show butterfly or waist-restricted hysteresis at 2 and 4 K (Fig. S38 and S39†).

Alternating current (ac) measurements for 1, 2, 5%Dy@1-Y and 100 and 200 mM 9 : 1 toluene/hexane frozen solutions of 1, all showed peaks in the out of phase susceptibility (*χ*′′). Cole–Cole isotherms of *χ*′′ *vs. χ*′ were fit to a generalised Debye model to extract the magnetic relaxation times and their associated distributions (Fig. S40–S65 and Tables S8–S12†).^26,27^ There is a wide distribution of relaxation rates for 1 and 2, as shown by the *α* parameters: 0.19–0.44 (1), and 0.30–0.50 (2; Fig. S66†); in solution the distributions increase with decreasing concentration (Fig. S67†). The temperature-dependence of the extracted relaxation rates were fit to a combination of Orbach, Raman and/or Quantum Tunnelling of the Magnetisation (QTM) processes (Fig. 4, Table 2 and Fig. S68–S72†). Rates were not weighted by their distributions, as this can lead to erroneous results in the case of extremely large distributions as we observe here.

**Fig. 4:**
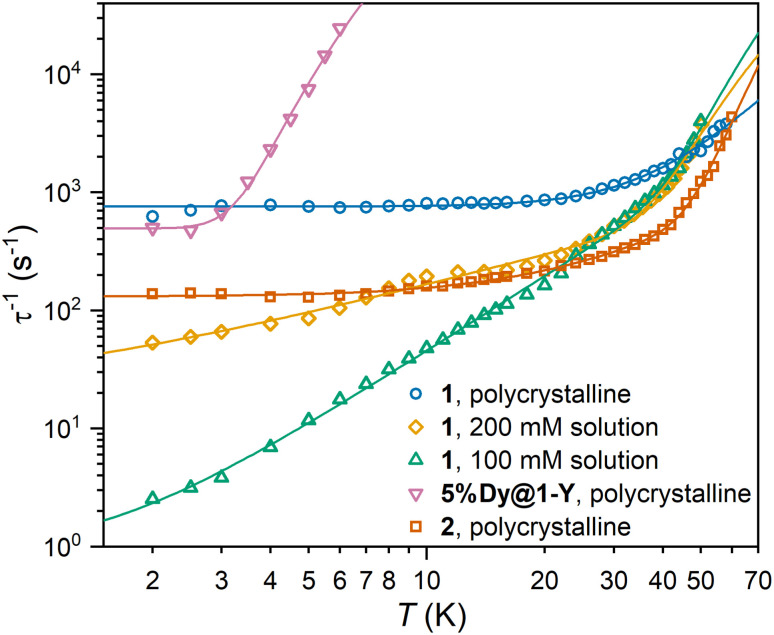
Temperature-dependence of relaxation rates for 1 (blue), 2 (red), 5%Dy@1-Y (pink) and 9 : 1 toluene/hexane frozen solutions of 1 at 200 mM (gold) and 100 mM (green). Solid lines show fit of relaxation profiles without weighting by rate distributions using the parameters in Table 2.

**Table 2:** Relaxation parameters for powder 1, 2 and 5%Dy@1-Y and frozen solutions of 1 obtained from fitting relaxation rates to *τ*^−1^ = *τ*^−1^_0_e^−*U*_eff_/*k*_B_*T*^ + *CT*^*n*^ + *τ*^−1^_QTM_, or *τ*^−1^ = *Pe*^*ω*/*T*^/(*e*^*ω*/*k*_B_*T*^ − 1)^2^ + *τ*^−1^_QTM_ in the case of 5%Dy@1-Y

Compound	State	*U*_eff_ (cm^−1^)	*τ*_0_ (s)	*C* (s^−1^ K^−*n*^)	*n*	*τ*_QTM_ (s)	*P* (s^−1^)	*ω* (cm^−1^)
1	Polycrystalline	—	—	7^+5^_−3_ × 10^−3^	3.17 ± 0.13	(1.32 ± 0.02) × 10^−3^	—	—
1	200 mM solution	210 ± 20	1.0^+0.8^_−0.4_ × 10^−6^	19^+8^_−5_	0.91 ± 0.11	6^+7^_−3_ × 10^−2^	—	—
1	100 mM solution	270 ± 40	2.0^+4^_−1.3_ × 10^−7^	0.34 ± 0.04	2.12 ± 0.05	1.2^+0.4^_−0.3_	—	—
5%Dy@1-Y	Polycrystalline	—	—	—	—	2.02^+0.14^_−0.13_ × 10^−3^	2.9^+0.9^_−0.7_ × 10^6^	20.6 ± 0.9
2	Polycrystalline	360 ± 20	6^+3^_−2_ × 10^−8^	0.40^+0.2^_−0.12_	1.80 ± 0.11	(7.7 ± 0.2) × 10^−3^	—	—

At high temperature, 2 shows Orbach relaxation with an effective barrier of 360 ± 20 cm^−1^, corresponding to the third excited state from CASSCF-SO calculations (Table S4†). For the pure powder sample of 1, no Orbach process is observed in the accessible range, owing to fast Raman and QTM; we propose QTM is more efficient for 1 than 2 due to the more charge dense Cl^−^ anion *versus* the more diffuse Br^−^ anion (Table S5†) disrupting the axial crystal field potential in 1 more than 2. However, upon dilution, the QTM rates are reduced,^28^ and frozen solutions of 1 reveal an Orbach process with *U*_eff_ around 210 ± 20 (200 mM) or 270 ± 40 cm^−1^ (100 mM), suggesting the process proceeds *via* the second excited state (Table S4†). We note the Orbach parameters of the two solutions overlap within 1 ESD, and that the 100 mM parameters are more reliable because the generalised Debye model is a better fit to the data, and the QTM-, Raman- and Orbach-dominated regimes are more distinct (Fig. 4). The *U*_eff_ barriers for solution 1 and powder 2 are moderate, lower than most CN6 *trans* alk-/aryl-oxide complexes (max. 1442 cm^−1^),^29^ but a true Orbach process does occur here, in contrast to many CN6 alk-/aryl-oxide complexes which can be modelled with an Arrhenius-like relaxation processes with low energies (15–36 cm^−1^) well below the first calculated excited state.^15–17,20,21,30^ These do not represent true Orbach processes, but can be attributed to phonon-pair driven Raman processes that appear as Arrhenius processes in the low temperature limit.^31^ Interestingly, 5%Dy@1-Y can be modelled by a phonon-pair driven Raman process with phonons of energy 20.6 ± 0.9 cm^−1^,^32^ which are either only present in 5%Dy@1-Y or are more strongly coupled to the electronic states than in 1, consistent with the different crystal packing.^32,33^ Data for 5%Dy@1-Y are equally well fit to a sum of power law Raman and QTM process (Fig. S72†).

We have characterised four mononuclear six-coordinate heteroleptic Ln(iii) complexes that each contain two aryloxides, one halide and three THF ligands. All complexes exhibit distorted octahedral geometries with Ln–aryloxide bonds that are >0.2 Å shorter than the other Ln–O/X bonds. The uncommon arrangement of *cis*-aryloxides results in a dominant *m*_*J*_ = ±15/2 ground state for Dy(iii) that can be directly observed by EPR spectroscopy as a *g*_*z*_ = 18.9(1) signal for 1 and 18.3(6) for 2. The electronic structure of the Dy(iii) complexes is sufficiently axial that they are zero-field SMMs with *U*_eff_ values of 270 ± 40 cm^−1^ (1 in frozen solution) and 360 ± 20 cm^−1^ (solid 2).

## Data availability

Supplementary information is available in the online version of the paper. Correspondence and requests for materials should be directed to N.F.C. and D.P.M. Crystallographic data for the structures reported in this article have been deposited at the Cambridge Crystallographic Data Centre, under deposition numbers CCDC 2366546–2366549.† Raw research data files supporting this publication are available from Figshare at https://doi.org/10.6084/m9.figshare.28638626. Apart from the data sets mentioned, all other data supporting the findings of this study are available within the article and ESI.†

## Conflicts of interest

There are no conflicts to declare.

## Supplementary Material

DT-054-D5DT00862J-s001

DT-054-D5DT00862J-s002
