# Supplementary material for: Direct characterisation of mJ = ±15/2 ground state in octahedral Dy(iii) single-molecule magnets
Source: Dalton Trans. 2025 Apr 17;54(19):7616–20. doi: 10.1039/d5dt00862j (PMC12020798; doi:10.1039/d5dt00862j)
Supplement: DT-054-D5DT00862J-s001 [file DT-054-D5DT00862J-s001.pdf]

Supplementary Information (SI) for Dalton Transactions.
This journal is © The Royal Society of Chemistry 2025

Direct characterisation of $m_J = \pm$ 15/2 ground state in octahedral Dy(III) single-molecule magnets

Vijay S. Parmar[a], Gemma K. Gransbury[a], Sophie C. Corner[a], Wei-Hao Chou[b,c], Stephen Hill[b,c,d], Richard E. P. Winpenny[a], Nicholas F. Chilton[a,e*] and David P. Mills[a*]

[a] *Department of Chemistry, University of Manchester, Manchester, M13 9PL, UK*
[b] *National High Magnetic Field Laboratory, Florida State University, Tallahassee, FL 32310, USA.*
[c] *Department of Physics, Florida State University, Tallahassee, FL 32306, USA.*
[d] *Department of Chemistry and Biochemistry, Florida State University, Tallahassee, FL 32306, USA.*
[e] *Research School of Chemistry, The Australian National University, Canberra, ACT, 2601, Australia.*

Table of Contents

1. General experimental procedures S2

2. Synthesis S3

3. NMR spectra S6

4. ATR-IR spectra S9

5. Crystallography S12

6. Computational details S27

7. EPR spectroscopy S34

8. Magnetism S44

9. References S82

1. General experimental procedures

All syntheses and manipulations were conducted under argon with the rigorous exclusion of oxygen and water using Schlenk line and glovebox techniques. THF and Diethyl ether were obtained from central solvent purification facility (SPS drying columns) and pentane and hexane were dried by refluxing over Na/K alloy and were stored over potassium mirrors. All solvents were degassed before use. For NMR spectroscopy benzene-d_6 was dried by refluxing over potassium, and was vacuum transferred and degassed by three freeze-pump-thaw cycles before use. Anhydrous $LnCl_3$ (Ln = Y or Dy) were purchased from Alfa Aesar and were used as received. $DyBr_3$[1], HOPh* and MOPh*[2] (M = Na, K; Ph* = 2,6-Ph_2-C_6H_4-Me, 2,6-bis(diphenyl-methyl)-4-methylphenyl) were prepared by literature procedures. ^1H (400 MHz) and $^{13}C\{^1H\}$ (100 MHz and 125 MHz) NMR spectra were obtained on an Avance III 400 MHz or 500 MHz spectrometers at 298 K. These were referenced to the solvent used, or to external TMS (^1H, ^{13}C). ATIR spectra were recorded as microcrystalline powders using a Bruker Tensor 27 ATR-Fourier Transform infrared (ATR-FTIR) spectrometer. Elemental analyses were performed by Mrs Anne Davies and Mr Martin Jennings at The University of Manchester, UK.

2. Synthesis

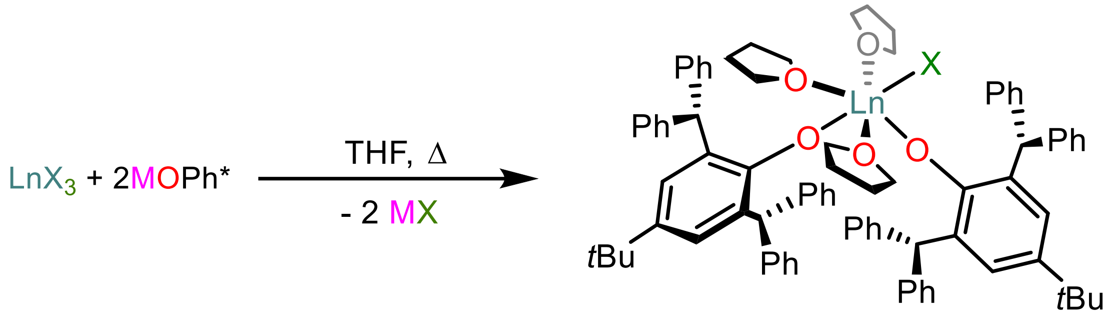

Ln = Y, Dy; M = Na, X = Cl (**1/1-Y**); M = K, X = Br (**2**)

Scheme S1. Synthesis of **1**, **2** and **1-Y**.

[Y(OPh*)$_2$(THF)$_3$Cl] (1-Y): A mixture of YCl_3 (0.392 g; 2 mmol) and NaOPh* (2.018 g; 4 mmol) in a Schlenk flask was cooled to -78 ºC and 30 ml of THF was added. The grey turbid reaction mixture was allowed to warm to room temperature and was heated to reflux for 8 hours with stirring continued overnight. The mixture was allowed to settle and cool to room temperature to give a colourless supernatant and grey precipitate, which was expected to be NaCl. The supernatant was filtered to another Schlenk flask and concentrated in volume to 5-8 mL for crystallization. Colourless crystals were formed at -25 ºC. Yield 56% (1.554 g, 1.12 mmol). The crystals were dried in vacuo to afford a white crystalline solid, which elemental analysis and NMR indicate is partially desolvated **1-Y**, with a molecule of lattice THF remaining in the sample. Anal. Calcd for $C_{84}H_{90}ClYO_5 \cdot C_4H_8O$: C, 76.81; H, 7.18 Found (%): C, 77.68; H, 7.15. ^{1}H NMR (400 MHz, benzene-d$_6$, 298 K, ppm) δ = 1.11 - 1.13 (m, 18 H, –C(C*H*$_3$)$_3$), 1.14-1.20 (br m, 16 H, THF), 3.39 (br m, 16 H, THF), 6.59 (s, 4 H, –C*H*(Ph)$_2$), 6.99 (d, J = 7.3 Hz, 8 H, Ar–*H$_{para}$*), 7.06 - 7.12 (m, 17 H, Ar–*H$_{ortho}$*), 7.20 (s, 4 H, Ar–*H$_{meta}$*), 7.20-7.42 (m, 16 H, Ar–*H$_{meta}$*) (see Figure S1). ^{13}C NMR (101 MHz, benzene-d$_6$, 298 K, ppm) δ = 25.1 (–C$_{THF}$), 31.4 (–C(*C*H$_3$)$_3$), 50.6 (–*C*H(Ph)$_2$), 69.2 (–C$_{THF}$), 127.5 (–*C*H$_{Ar}$), 127.7 (–*C*H$_{Ar}$), 127.9 (–*C*H$_{Ar}$) 128.1 (–*C*H$_{Ar}$)

130.1 (–C_{Ar}) 145.6 (–C_{Ar}) (see Figures S2 – S3). FTIR (ATR, microcrystalline) ũ: 3083 (w), 3058 (w), 3025 (w), 2962 (w), 2900 (w), 2879 (w), 2865 (w), 1599 (w), 1583 (w), 1492 (w), 1469 (w), 1445 (w), 1391 (w), 1361 (w), 1293 (w), 1278 (w), 1260 (w), 1215 (w), 1184 (w), 1155 (w), 1091 (w), 1075 (w), 1015 (w), 956 (w), 915 (w), 892 (w), 863 (w), 847 (w), 799 (w), 767 (w), 744 (w), 699 (w), 676 (w), 641 (w), 635 (w), 623 (w), 617 (w), 604 (w), 588 (w), 571 (w), 557 (w), 536 (w), 512 (w), 501 (w), 479 (w), 473 (w), 460 (w), 442 (w), 432 (w) cm^{-1} (Figures S6 and S10).

[Dy(OPh*)$_2$(THF)$_3$Cl] (1): Complex **1** was synthesised similarly to **1-Y** with $DyCl_3$ (0.268 g; 1 mmol) and NaOPh* (1.009 g; 2 mmol). For crystallisation, the supernatant was filtered to another Schlenk flask and dried in vacuum. 5 mL of diethyl ether was added to the resultant beige powder to dissolve it and 5 mL hexane was layered on top for slow diffusion procedure. Colourless crystals were formed at 0 °C. Yield 48% (0.693 g, 0.48 mmol). The crystals were dried in vacuo to afford a white crystalline solid, which elemental analysis indicate is **1**. Anal. Calcd for $C_{84}H_{90}ClDyO_5{\cdot}C_4H_{10}O$: C, 72.81; H, 6.94. Found: C, 71.06; H, 6.81. Elemental analysis results show lower carbon values than predicted, which we attribute to carbide formation from incomplete combustion. μ_{eff} = 10.24 μ_B mol^{-1}, χT_{298} = 13.10 cm^3 mol^{-1} K (Evans method)[3,4] (Figure S4). FTIR (ATR, microcrystalline) ũ: 3566 (v. w), 3083 (w), 3058 (w), 3025 (w), 2962 (w), 2900 (w), 2879 (w), 2865 (w), 1599 (w), 1583 (w), 1492 (w), 1469 (w), 1445 (w), 1391 (w), 1361 (w), 1293 (w), 1278 (w), 1260 (w), 1215 (w), 1184 (w), 1155 (w), 1091 (w), 1075 (w), 1015 (w), 956 (w), 915 (w), 892 (w), 863 (w), 847 (w), 799 (w), 767 (w), 744 (w), 699 (w), 676 (w), 641 (w), 635 (w), 623 (w), 617 (w), 604 (w), 588 (w), 571 (w), 557 (w), 536 (w), 512 (w), 501 (w), 479 (w), 473 (w), 460 (w), 442 (w), 432 (w), 399 (w) cm^{-1} (Figures S7 and S10).

[Dy(OPh*)$_2$(THF)$_3$Br] (2): Complex **2** was synthesised similarly to **1** with $DyBr_3$ (0.402 g; 1 mmol) and KOPh* (1.04 g; 2 mmol). Colourless crystals were formed at 0 °C from slow diffusion of 5 mL of hexane layered on 5-6 mL diethyl ether solution of the product. Yield 50% (0.714 g, 0.50 mmol). The crystals were dried in vacuo to afford a white crystalline solid, which elemental analysis indicate is desolvated **2**, anal. calcd for $C_{84}H_{90}BrDyO_5$: C, 70.95; H, 6.38. Found: C, 69.53; H, 6.43. Elemental analysis results show lower carbon values than predicted, which we attribute to carbide formation from incomplete combustion. μ_{eff} = 10.16 μ_B mol^{-1}, χT_{298} = 12.90 cm^3 mol^{-1} K (Evans method)[3,4] (Figure S5). FTIR (ATR, microcrystalline) ũ: 3551 (w), 3083 (w), 3058 (w), 3023 (w), 2900 (w), 2865 (w), 1599 (w), 1492 (s), 1472 (w), 1445 (v. w), 1391 (w), 1361 (s, br), 1344 (w), 1291 (m, br), 1276 (m, br), 1248 (m, br), 1184 (w), 1122 (s, br), 1075 (m, br), 1030 (w), 1013 (m, br), 913 (m, br), 892 (w), 861 (s, br), 847 (s, br), 762 (s, br), 738 (m, br), 699 (s), 676 (s), 641 (m, br), 617 (m, br), 604 (s), 586 (s), 573 (s), 536 (s, br), 520 (w), 506 (w), 495 (w), 485 (w), 467 (m, br), 438 (m, br), 409 (s) cm^{-1} (Figures S8 and S10).

5%Dy@[Y(OPh*)$_2$(THF)$_3$Cl] (5%Dy@1-Y). The synthetic procedure and crystallization employed here are similar to that of **1-Y** using $DyCl_3$ (0.013 g; 0.05 mmol) and YCl_3 (0.186 g; 0.95 mmol). For crystallisation, 5 mL pentane was layered on top of the concentrated solution of the product for slow diffusion. The crystals were obtained as colourless block-shaped crystals at -25°C (0.850 g, 0.65 mmol; 65%). Anal. Calcd for $C_{84}H_{90}ClDy_{0.05}Y_{0.95}O_5{\cdot}C_5H_{12}$: C, 77.47; H, 7.45. Found: C, 76.16; H, 7.04. FTIR (ATR, microcrystalline) ũ: 3551 (w), 3081 (w), 3056 (w), 3025 (w), 2996 (w), 2959 (w), 2898 (w), 2867 (w), 1599 (w), 1583 (w), 1492 (w), 1469 (w), 1443 (w), 1391 (w), 1361 (w), 1346 (w), 1293 (w), 1276 (w), 1184 (w), 1155 (w), 1122 (w), 1077 (w), 1028 (w), 1011 (v. w), 956 (w), 915 (w), 892 (w), 863 (w), 845

(w), 765 (w), 742 (w), 699 (w), 676 (w), 639 (w), 631 (w), 619 (w), 604 (w), 588 (w), 573 (w), 536 (w), 504 (w), 491 (w), 473 (w), 462 (w), 405 (w) cm^{-1} (Figures S9 and S10).

3. NMR spectra

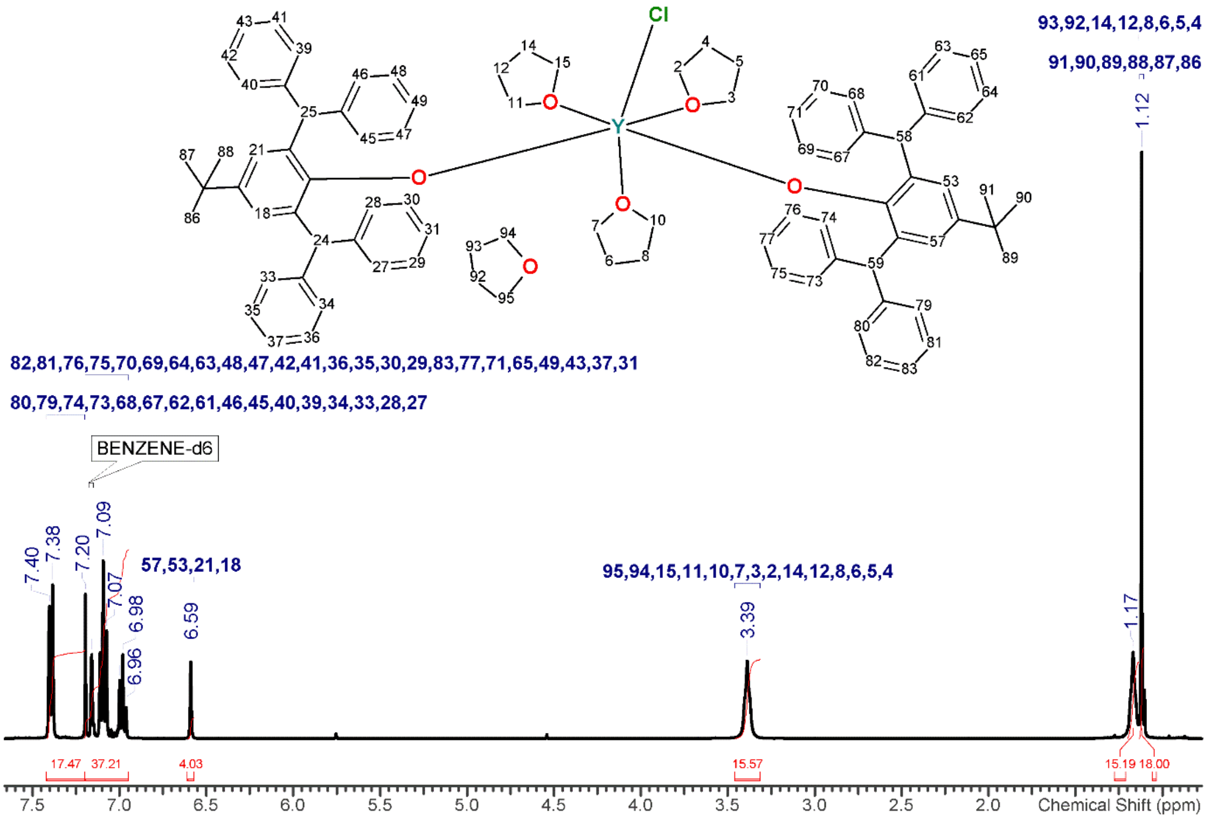

Figure S1. ^{1}H NMR spectrum of **1-Y** [$Y(OPh^*)_2(THF)_3Cl$] in benzene-d_6.

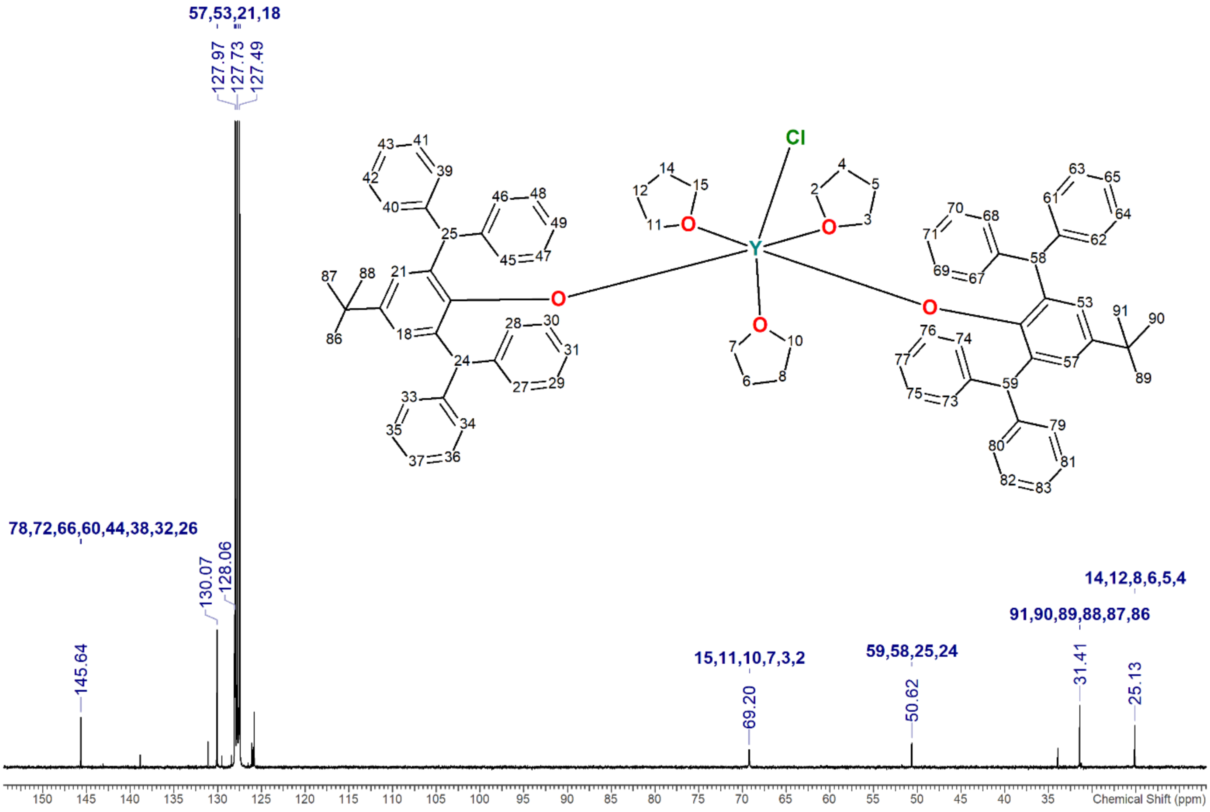

Figure S2. ^{13}C NMR spectrum of **1-Y** [Y(OPh*)$_2$(THF)$_3$Cl] in benzene-d$_6$.

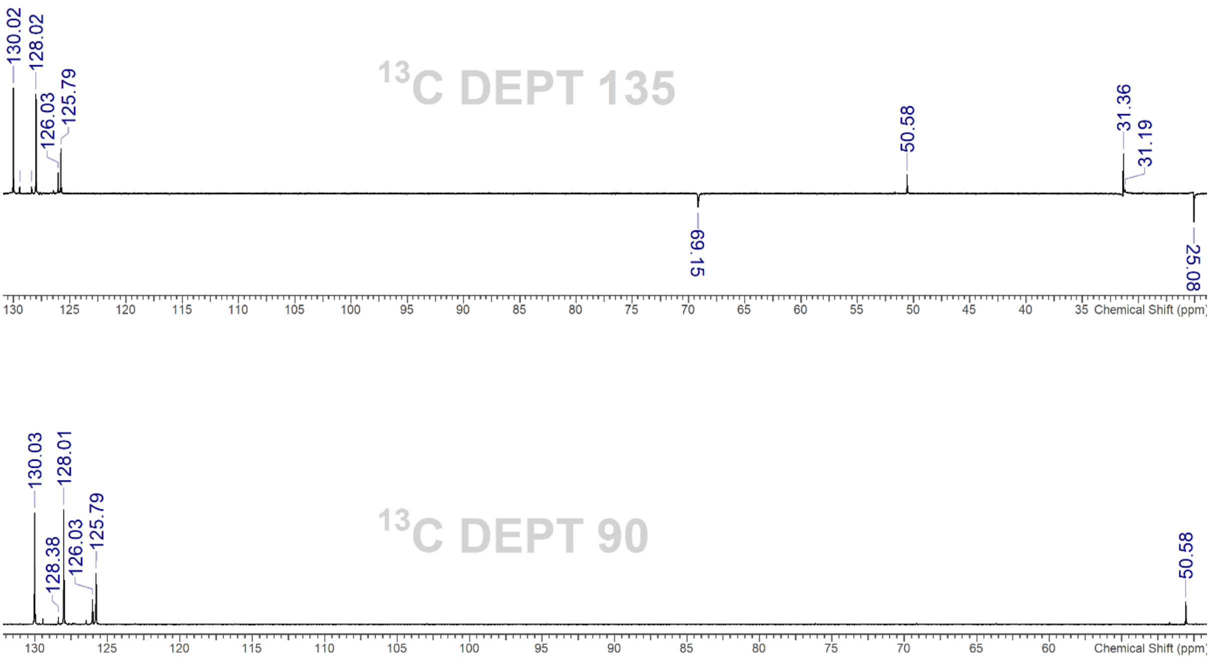

Figure S3. ^{13}C DEPT 135 (top) and DEPT 90 (bottom) NMR spectrum of **1-Y** [Y(OPh*)$_2$(THF)$_3$Cl] in benzene-d$_6$.

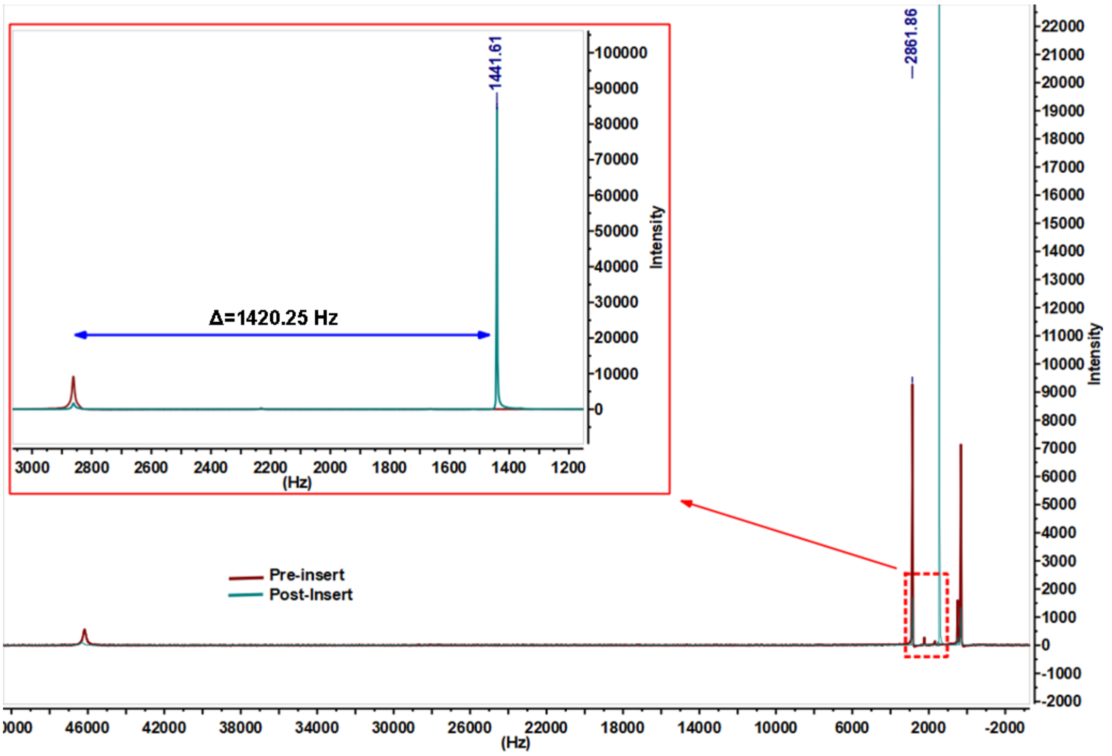

Figure S4. ^{1}H NMR spectrum of **1** in benzene-d6 for Evans NMR method to calculate μ_{eff} values. Red line is pre-insert, cyan line is post-insert of sealed solvent capillary.

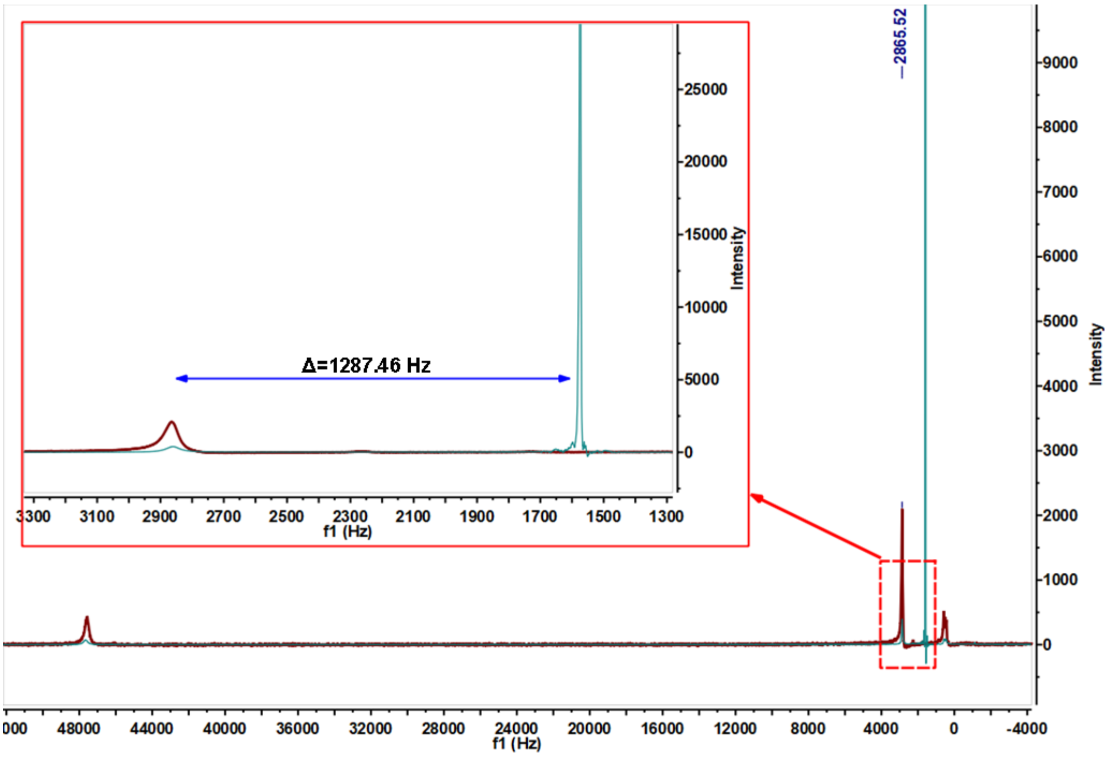

Figure S5. ^{1}H NMR spectrum of **2** in benzene-d6 for Evans NMR method to calculate μ_{eff} values. Red line is pre-insert, cyan line is post-insert of sealed solvent capillary.

4. ATR-IR spectra

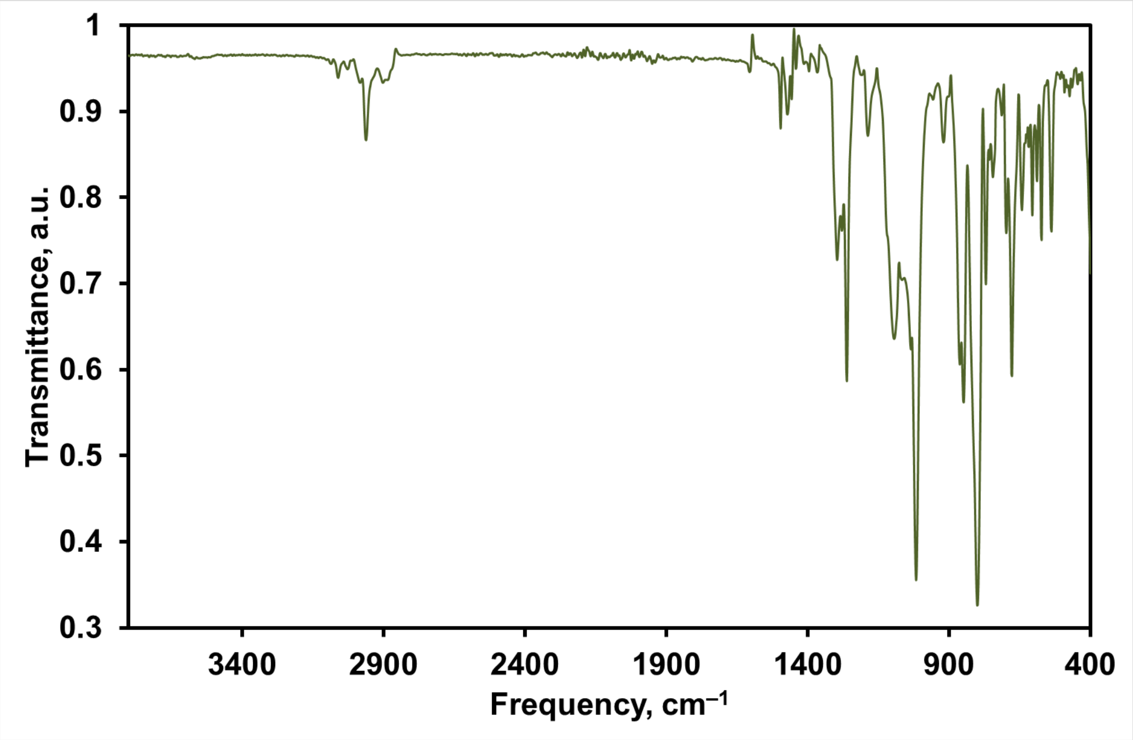

Figure S6. ATIR spectrum of **1-Y**, 400-4000 cm^{-1}.

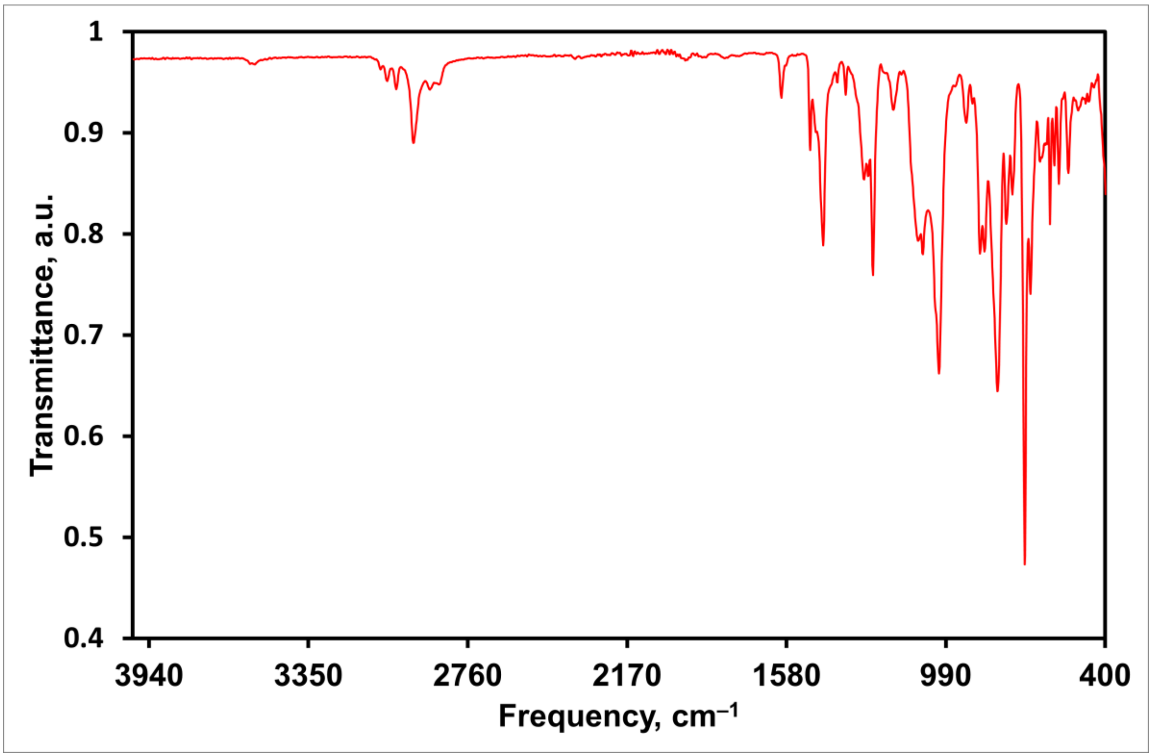

Figure S7. ATIR spectrum of **1**, 400-4000 cm^{-1}.

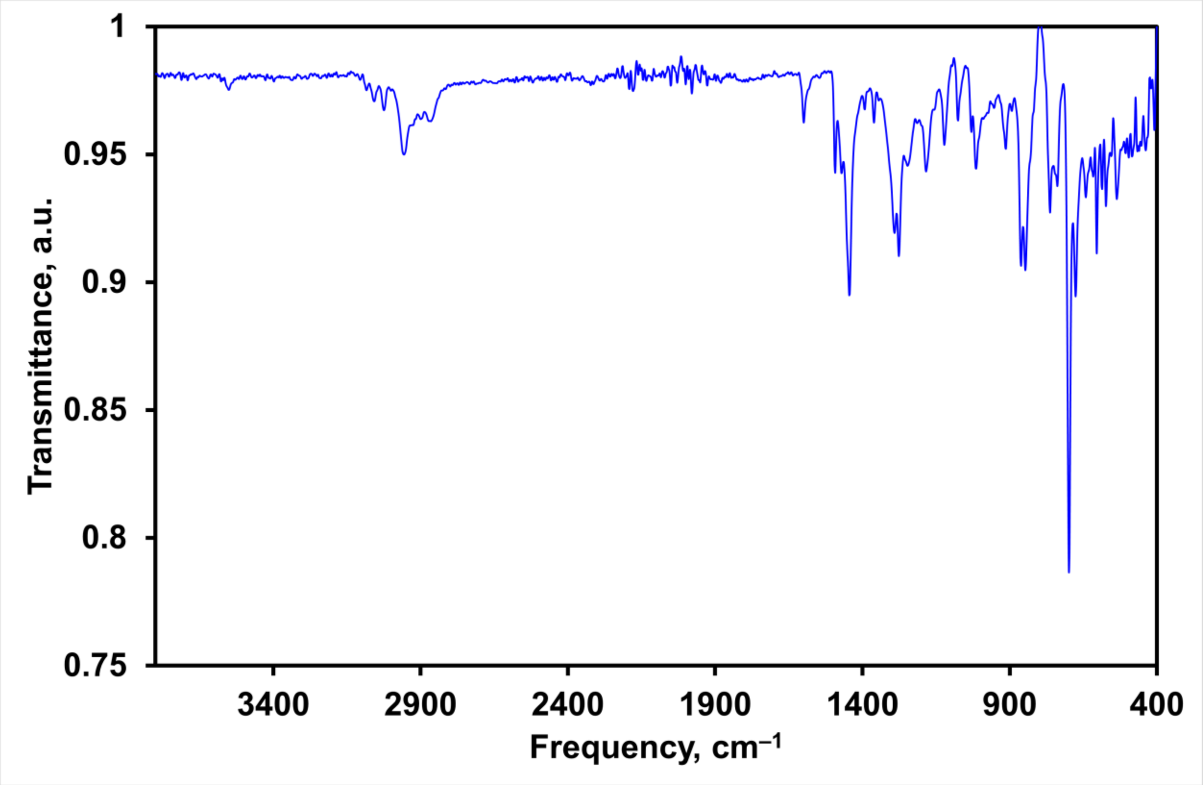

Figure S8. ATIR spectrum of **2**, 400-4000 cm^{-1}.

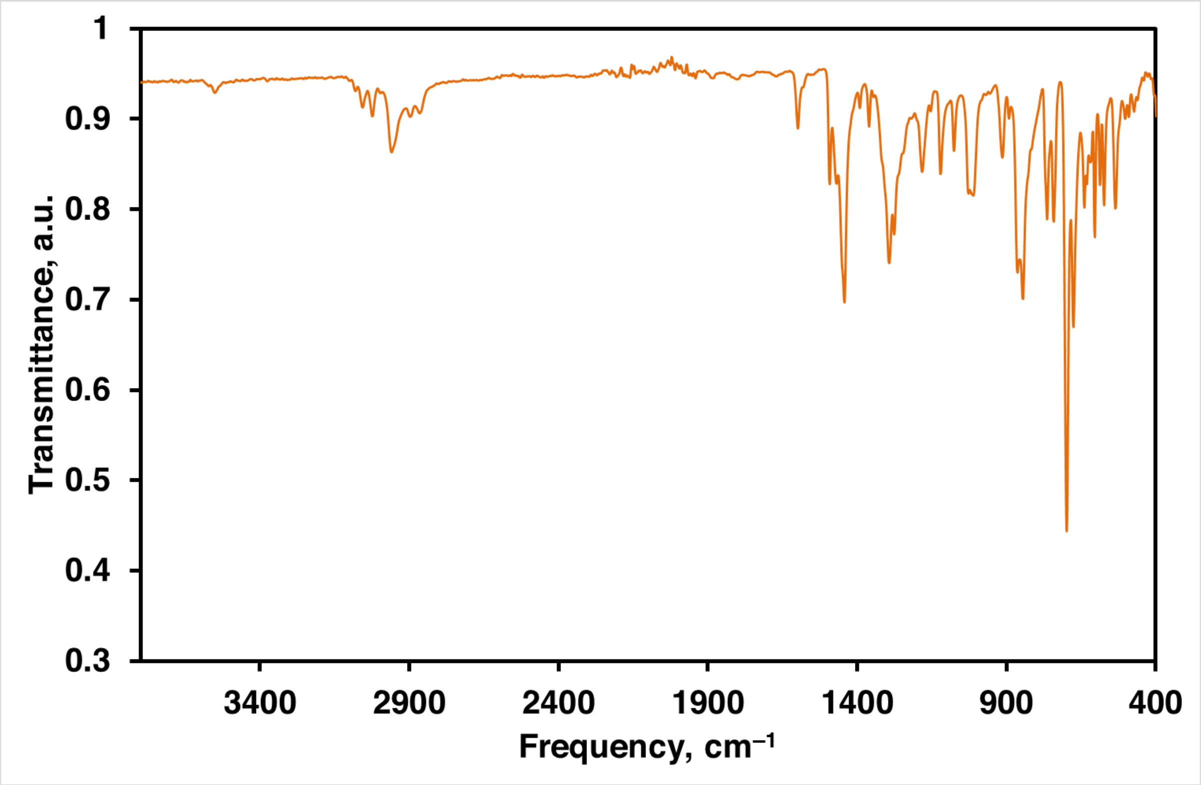

Figure S9. ATIR spectrum of **5%Dy@1-Y**, 400-4000 cm^{-1}.

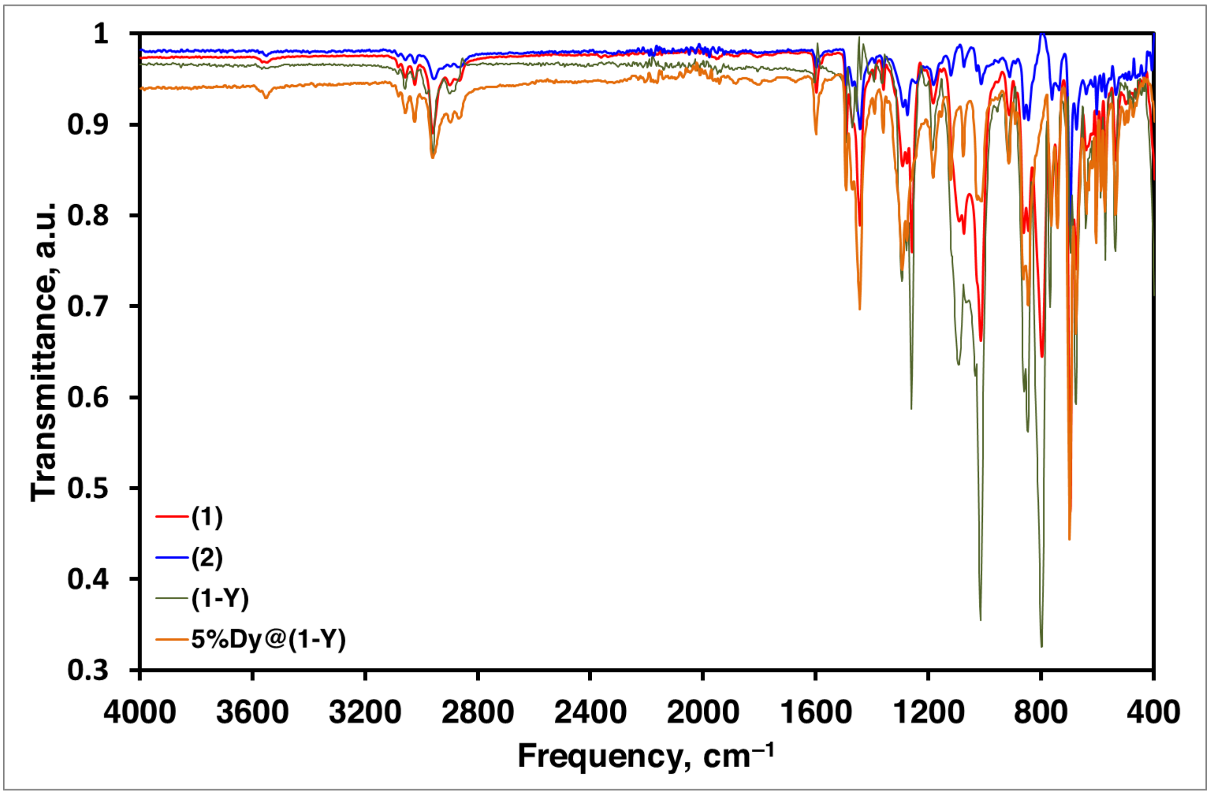

Figure S10. ATIR spectra of **1**, **2**, **1-Y** and **5%Dy@1-Y** 400 - 4000 cm^{-1}.

5. Crystallography

The crystal data for complexes **1**, **2**,**1-Y** and **5%Dy@1-Y** are compiled in Table S1. Crystals of **1** and **2** were examined using an Oxford Diffraction Supernova diffractometer, equipped with CCD area detector and a mirror-monochromated Mo K_{α} radiation (λ = 0.71073 Å). Crystals of **1-Y** and **5%Dy@1-Y** were examined using a Rigaku XtalLAB AFC11 diffractometer with a CCD area detector and a graphite-monochromated Cu Kα (λ = 1.54184 Å). Intensities were integrated from data recorded on 1° frames by ω rotation. Cell parameters were refined from the observed positions of all strong reflections in each data set. A multi-scan absorption correction with a beam profile was applied.[5] The structures were solved using direct methods by SHELXS; the datasets were refined by full-matrix least-squares on all unique F^2 values.[6] CrysAlisPro[5] was used for control and integration, and SHELX[6,7] was employed through OLEX2[8] for structure solution and refinement. OLEX2 was used to employ a solvent mask for **5%Dy@1-Y** to account for disordered lattice pentane which could not be modelled satisfactorily, the solvent accessible volume per cell was 754.0 Å^3 with a total electron count of 156.9 e, corresponding to two molecules of pentane per cell (one per formula unit of **5%Dy@1-Y**). ORTEP-3[9] and POV-Ray[10] were employed for molecular graphics. CCDC 2366546-2366549 contain the supplementary crystal data for this article. These data can be obtained free of charge from the Cambridge Crystallographic Data Centre via www.ccdc.cam.ac.uk/data_request/cif.

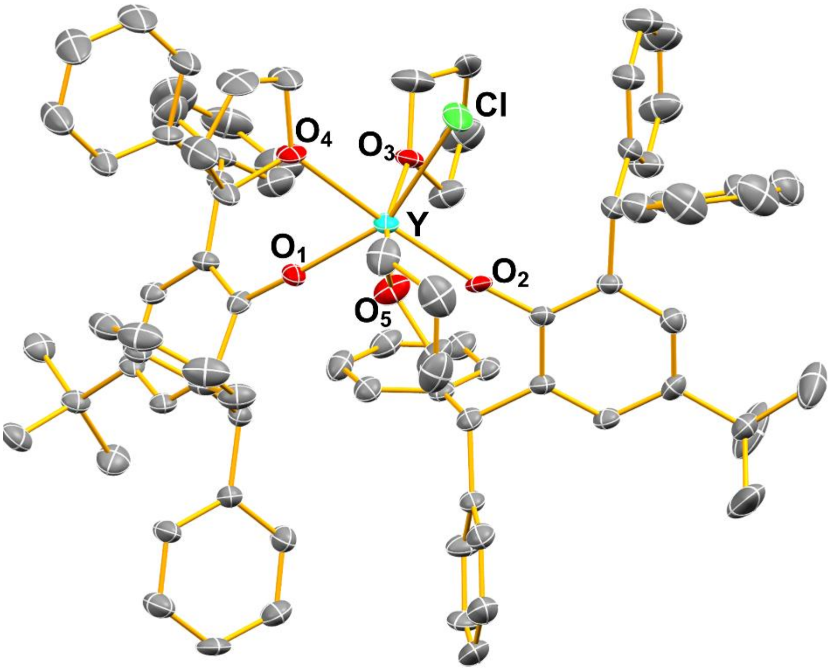

Figure S11. View of the molecular structure of [Y(OPh*)2(THF)3Cl] (**1-Y**) from single crystal XRD at 150 K with thermal ellipsoids drawn at 40% probability level (Y teal, Cl green, O red, C grey). H atoms and lattice solvent are omitted for clarity.

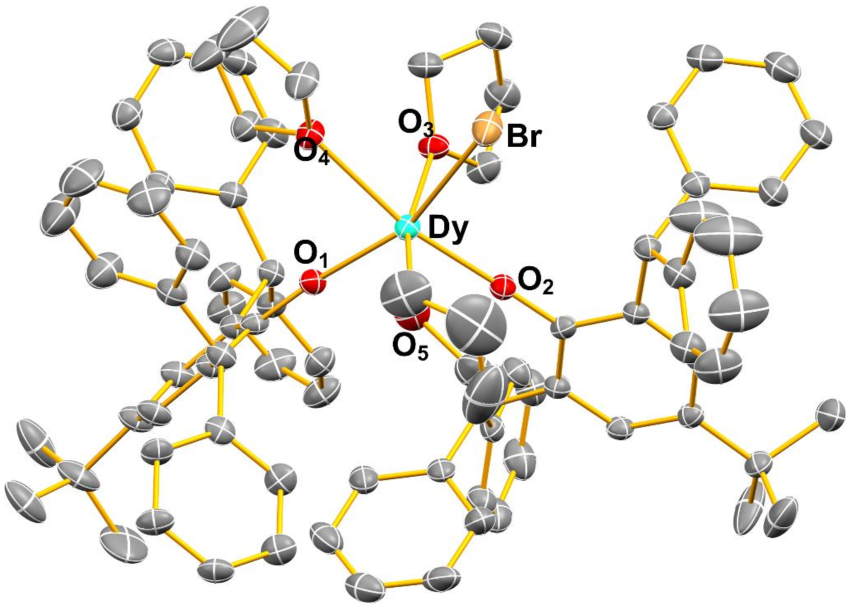

Figure S12. View of the molecular structure of [Dy(OPh*)2(THF)3Br] (**2**) from single crystal XRD at 150 K with thermal ellipsoids drawn at 40% probability level (Dy teal, Br brown, O red, C grey). H atoms and lattice solvent are omitted for clarity.

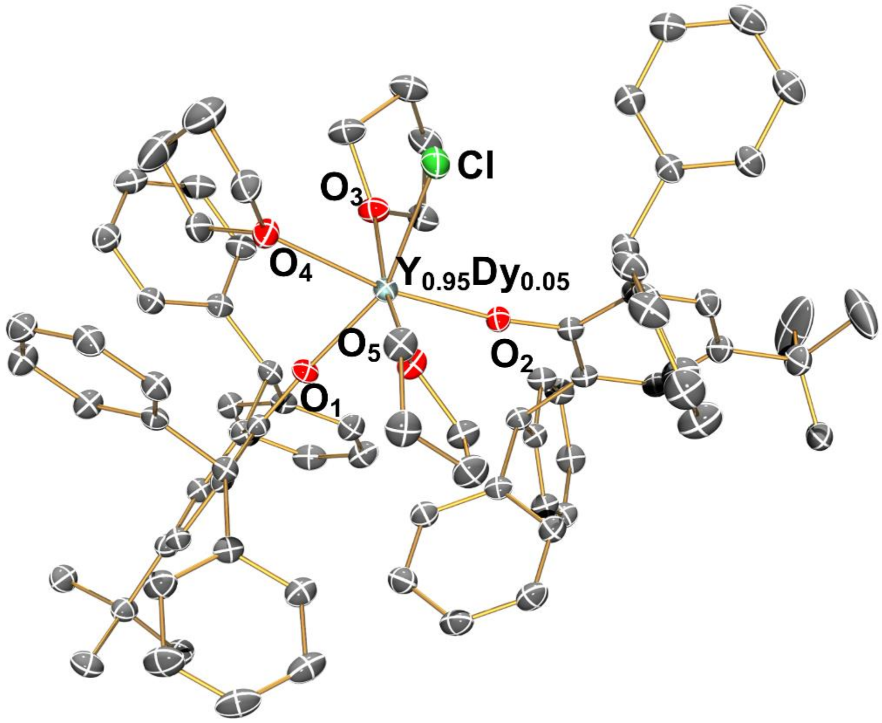

Figure S13. View of the molecular structure of $[Dy_{0.05}Y_{0.95}(OPh^*)_2(THF)_3Cl]$ (**5%Dy@1-Y**) from single crystal XRD at 100 K with thermal ellipsoids drawn at 40% probability level (Dy/Y teal, Cl green, O red, C grey). H atoms and lattice solvent are omitted for clarity.

Table S1. Crystallographic data for **1**, **1-Y**, **5%Dy@1-Y** and **2.**

	**1**	**2**	**1-Y**	**5%Dy@1-Y**
**Formula**	$C_{92}H_{110}ClDyO_7$	$C_{93}H_{111}BrDyO_5$	$C_{96}H_{114}ClO_8Y$	$C_{89}H_{102}ClO_5$ $Dy_{0.05}Y_{0.95}$
**Fw, g mol^{-1}**	1525.74	1551.22	1520.23	1379.74
**Cryst size, mm**	0.227 × 0.066 × 0.043	0.204 × 0.144 × 0.118	0.203 × 0.172 × 0.170	0.147 × 0.121 × 0.055
**Crystal system**	triclinic	triclinic	triclinic	triclinic
**Space group**	P-1	P-1	P-1	P-1
**Collection Temperature (K)**	149.9(4)	150.03(10)	150(2)	100.02(13)
**a, (Å)**	13.0454(5)	12.1093(4)	12.06710(10)	13.6646(3)
**b, (Å)**	16.6596(6)	15.3634(7)	15.4384(2)	18.2180(5)
**c, (Å)**	21.3210(6)	23.6294(9)	23.3235(2)	18.4204(4)
**α, (°)**	112.183(3)	105.495(4)	104.0730(10)	118.073(2)
**β, (°)**	92.607(3)	93.553(3)	91.5630(10)	96.087(2)
**γ, (°)**	107.522(3)	94.744(3)	94.2090(10)	93.434(2)
**V, (Å^3)**	4024.7(3)	4205.5(3)	4198.74(8)	3991.66(18)
**Z**	2	2	2	2
**ρ_{calc} g cm^{-3}**	1.257	1.225	1.202	1.088 + solvent
**μ, mm^{-1}**	1.015	1.412	1.699	1.886
**No. of reflections measured**	33817	36781	66418	45707
**No. of unique reflections, R_{int}**	16438, 0.1191	17189, 0.0593	17120, 0.0251	15922, 0.0255
**No. of reflections with F^2 > 2s(F^2)**	10823	13384	16071	14793
**Transmission coefficient range**	0.642-1.000	0.826-1.000	0.89935-1.00000	0.737-1.000
**R_1 ($I > 2\sigma(I)$)**	0.0684	0.0511	0.0397	0.0323
**wR_2 (all data)**	0.1555	0.1277	0.1102	0.0867
**S^a**	0.963	1.082	1.066	1.045
**Parameters, Restraints**	976, 1096	1071, 2119	1136, 2132	857, 909
**Max./min. difference map, e Å^{-3}**	1.642, -2.103	1.253, -1.117	0.538, -0.672	0.913, -0.556

^{a}Conventional $R = \Sigma||F_o| - |F_c||/\Sigma|F_o|$; $R_w = [\Sigma w(F_o^2 - F_c^2)^2/\Sigma w(F_o^2)^2]^{1/2}$; $S = [\Sigma w(F_o^2 - F_c^2)^2/\text{no. data} - \text{no. params})]^{1/2}$ for all data.

Table S2. Continuous Shape Measures (CShM)[11] calculations for complexes **1**, **2**, **1-Y** and **5%Dy@1-Y**.

**Structure**[a]	HP-6	PPY-6	OC-6	TPR-6	JPPY-6
**1**	32.351	20.927	2.099	10.702	25.348
**2**	32.311	21.913	2.208	10.893	26.488
**1-Y**	33.305	24.800	1.188	13.147	29.304
**5%Dy@1-Y**	32.535	25.030	1.157	13.070	28.847

[a] HP-6 Hexagon (D_{6h})
PPY-6 Pentagonal pyramid (C_{5v})
OC-6 Octahedron (Oh)
TPR-6 Trigonal prism (D_{3h})
JPPY-6 Johnson pentagonal pyramid J2 (C_{5v})

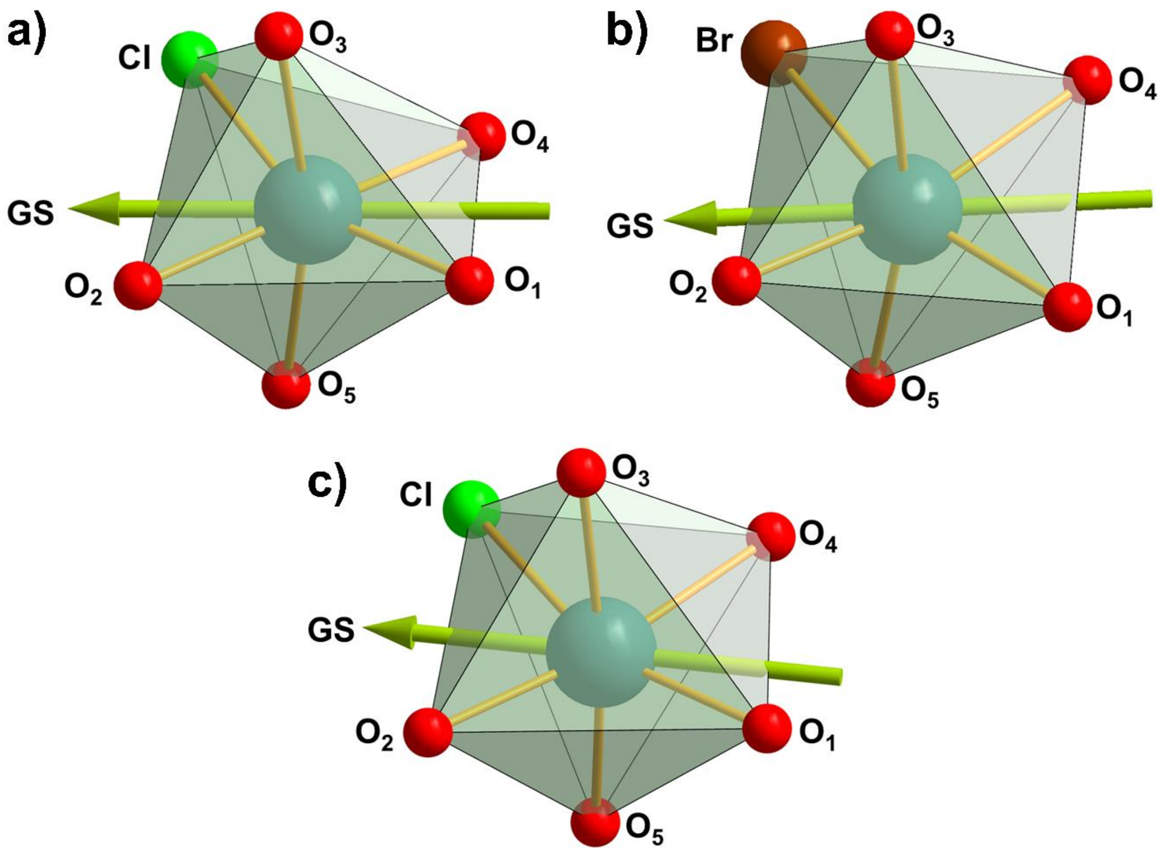

Figure S14. View of the first coordination sphere geometry in (a) **1**, (b) **2** and (c) **5%Dy@1-Y** from single crystal XRD at 150 K (Dy cyan, Cl green, Br dark brown, O red, C grey). Arrows represent CASSCF-SO calculated magnetic axis (g_z vector), GS (green).

Table S3. Selected bond distances (Å) and angles (°). X = Cl (**1**, **1-Y**, **5%Dy@1-Y**), Br (**2**).

	**1**	**2**	**1-Y**	**5%Dy@1-Y**
Ln-X	2.6462(14)	2.8301(5)	2.6455(4)	2.6269(4)
Ln-O1	2.141(3)	2.130(3)	2.1231(11)	2.1341(10)
Ln-O2	2.109(4)	2.106(3)	2.1034(12)	2.1145(10)
Ln-O3	2.375(4)	2.376(3)	2.3332(13)	2.3569(11)
Ln-O4	2.420(4)	2.447(3)	2.4210(13)	2.4063(12)
Ln-O5	2.398(4)	2.393(3)	2.3680(13)	2.3175(11)
O1-Ln-X	155.54(11)	157.83(8)	158.00(4)	157.62(3)
O1-Ln-O3	91.39(14)	96.35(10)	93.23(5)	93.05(4)
O1-Ln-O4	80.84(14)	81.21(10)	80.07(5)	81.18(4)
O1-Ln-O5	98.92(15)	93.84(11)	95.31(5)	93.76(4)
O2-Ln-X	95.78(10)	95.90(7)	96.30(3)	92.54(3)
O2-Ln-O1	108.16(14)	105.86(10)	105.67(5)	109.83(4)
O2-Ln-O3	85.21(14)	84.10(11)	86.72(5)	89.69(4)
O2-Ln-O4	167.35(14)	167.76(10)	174.24(5)	168.98(4)
O2-Ln-O5	84.28(15)	85.04(12)	88.22(5)	86.28(4)
O3-Ln-X	85.60(10)	89.93(7)	86.84(4)	86.21(3)
O3-Ln-O4	103.78(14)	85.20(10)	93.50(5)	89.18(4)
O3-Ln-O5	167.14(14)	166.79(11)	170.99(5)	172.95(4)
O4-Ln-X	76.36(10)	78.15(6)	77.97(3)	76.45(3)
O4-Ln-O5	85.55(15)	104.70(12)	90.79(5)	93.67(4)
O5-Ln-X	88.10(11)	83.71(8)	86.32(4)	88.19(3)

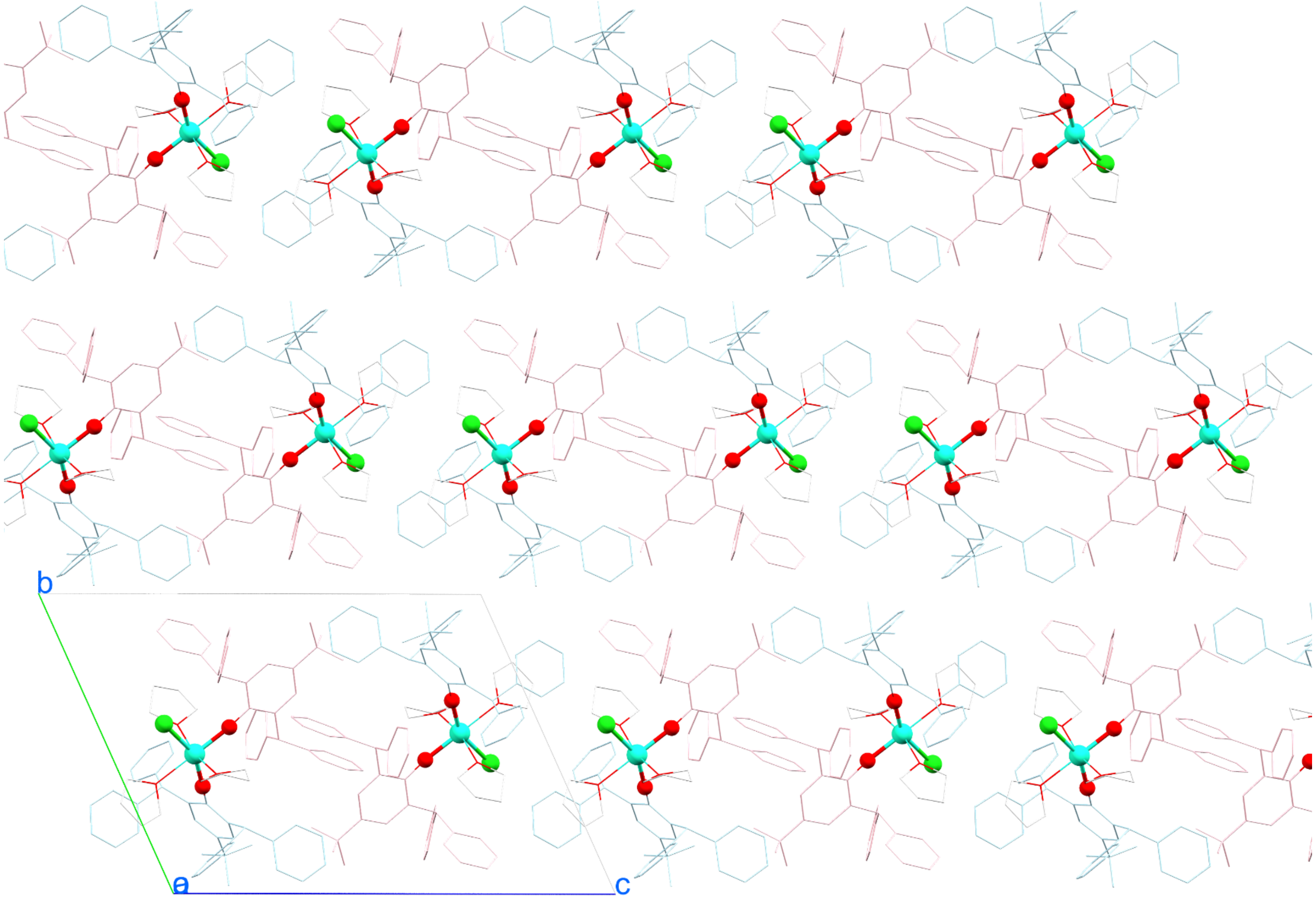

Figure S15. View of the crystal packing in [Dy(OPh*)$_2$(THF)$_3$Cl] **1** along the a-axis (Dy cyan, Cl green, O red, C from THF in grey, C from OPh*$_1$ in blue, C from OPh*$_2$ in red). H atoms and lattice solvent are omitted for clarity.

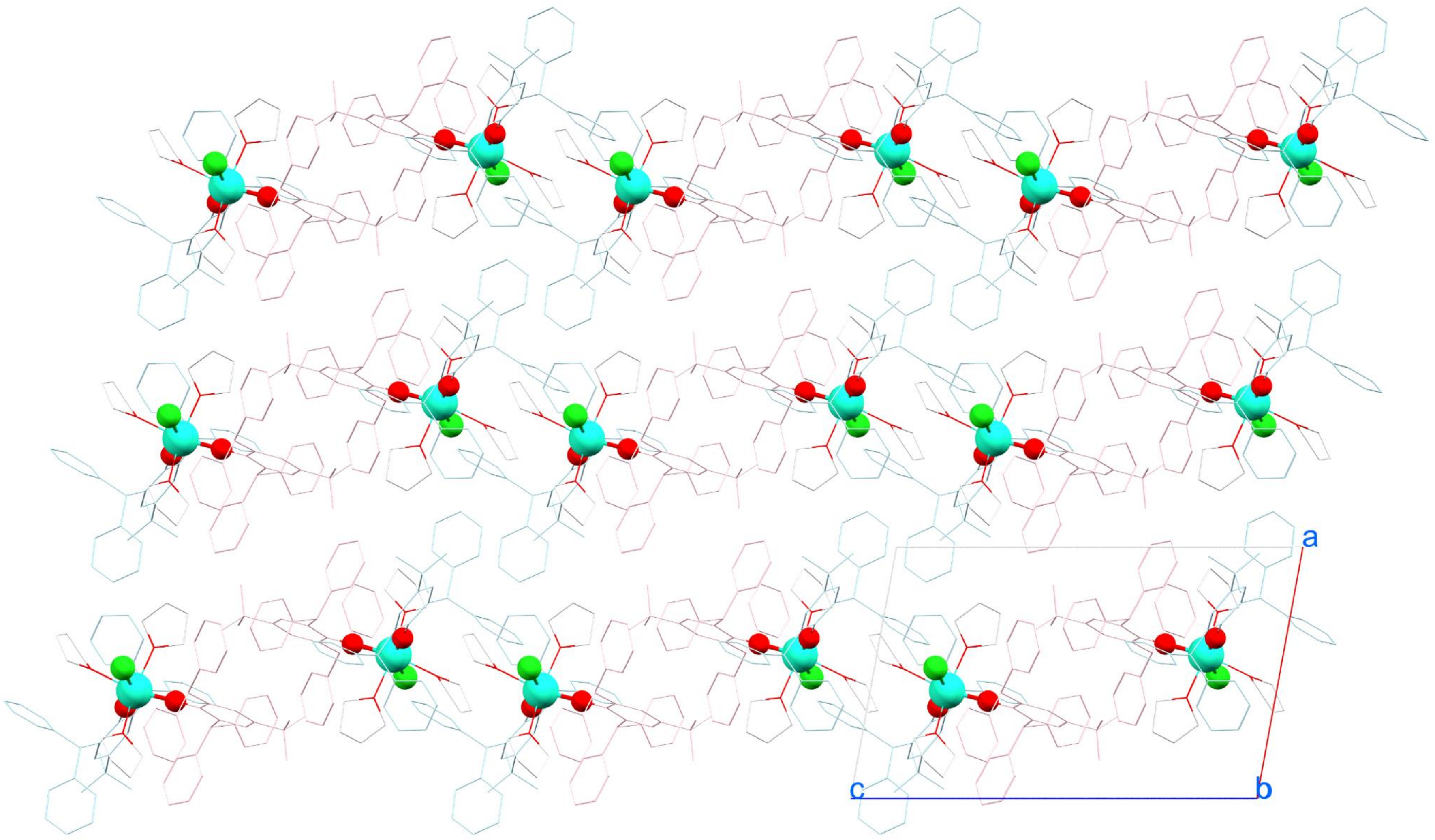

Figure S16. View of the crystal packing in [Dy(OPh*)$_2$(THF)$_3$Cl] **1** along the b-axis (Dy cyan, Cl green, O red, C from THF in grey, C from OPh*$_1$ in blue, C from OPh*$_2$ in red). H atoms and lattice solvent are omitted for clarity.

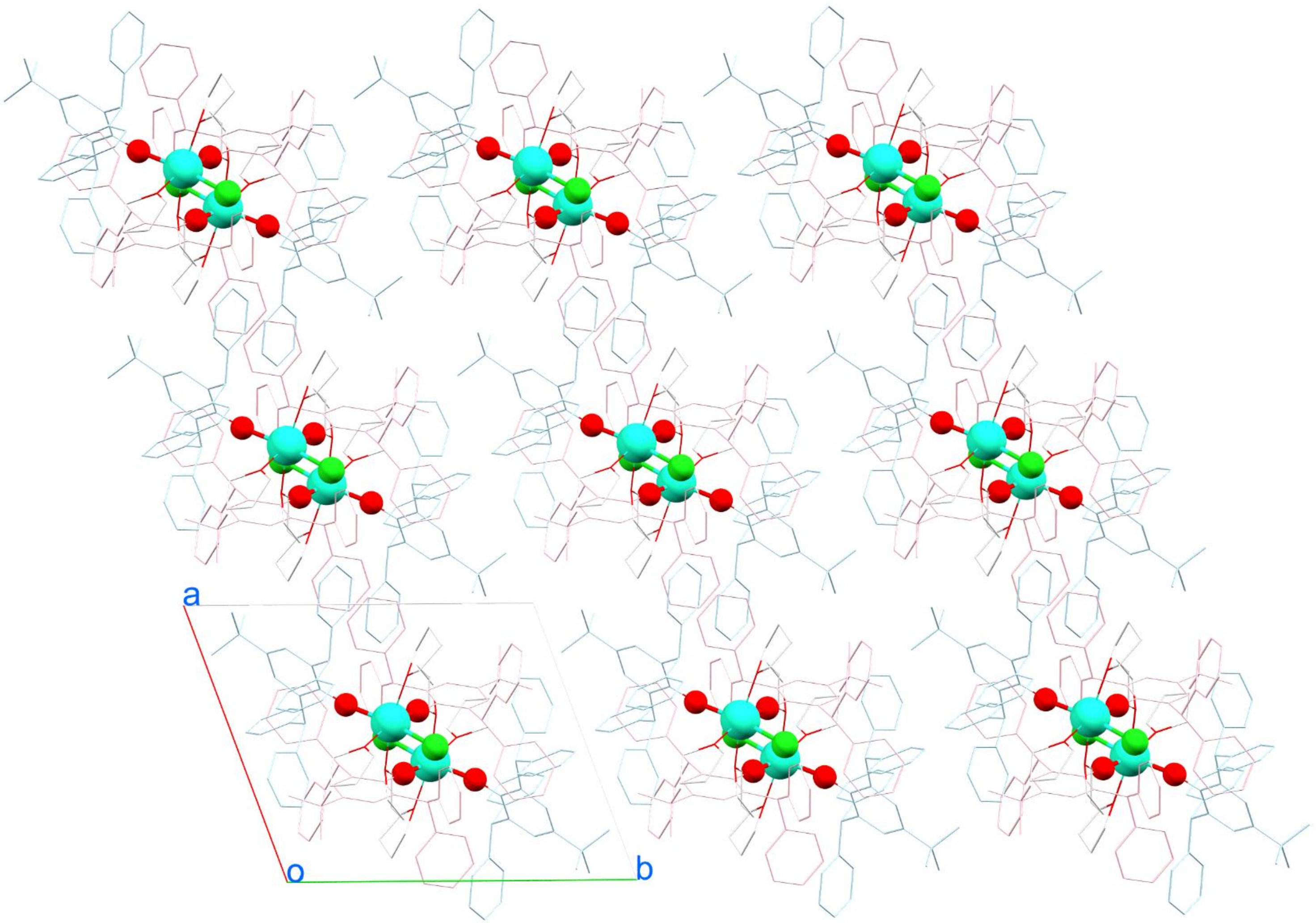

Figure S17. View of the crystal packing in [Dy(OPh*)$_2$(THF)$_3$Cl] **1** along the c-axis (Dy cyan, Cl green, O red, C from THF in grey, C from OPh*$_1$ in blue, C from OPh*$_2$ in red). H atoms and lattice solvent are omitted for clarity.

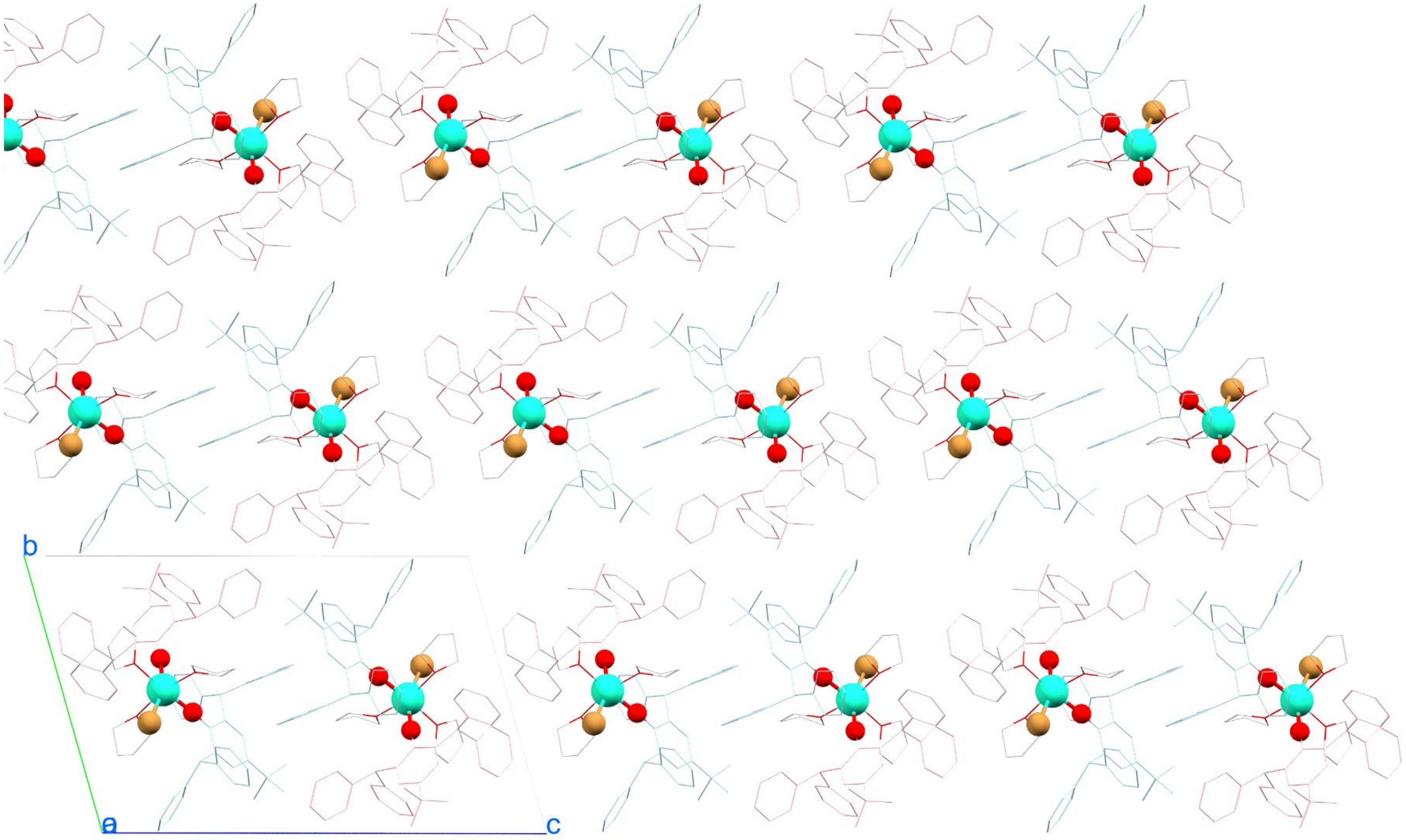

Figure S18. View of the crystal packing in [Dy(OPh*)$_2$(THF)$_3$Br] **2** along the a-axis (Dy cyan, Br brown, O red, C from THF in grey, C from OPh*$_1$ in blue, C from OPh*$_2$ in red). H atoms and lattice solvent are omitted for clarity.

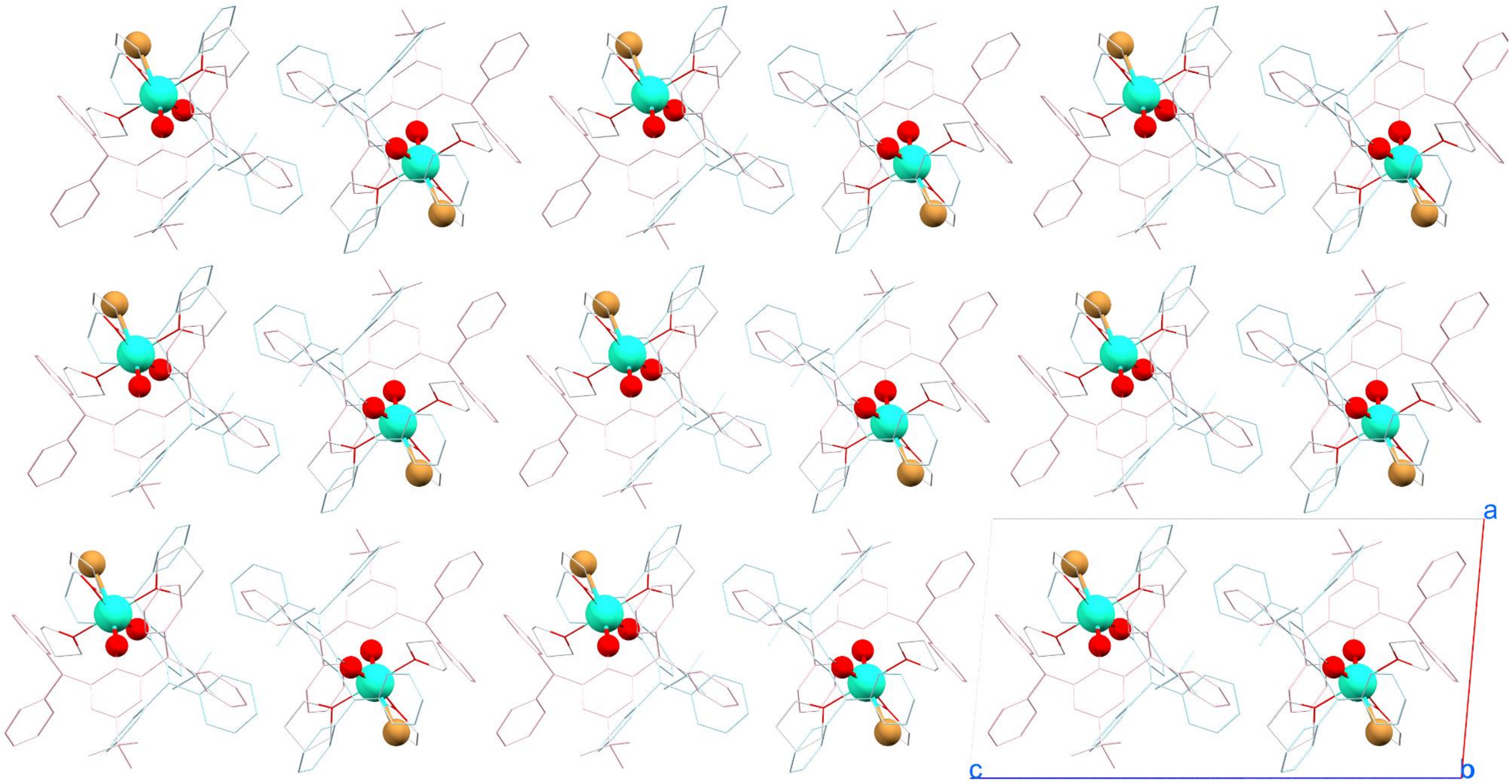

Figure S19. View of the crystal packing in [$Dy(OPh^*)_2(THF)_3Br$] **2** along the b-axis (Dy cyan, Br brown, O red, C from THF in grey, C from OPh^*_1 in blue, C from OPh^*_2 in red). H atoms and lattice solvent are omitted for clarity.

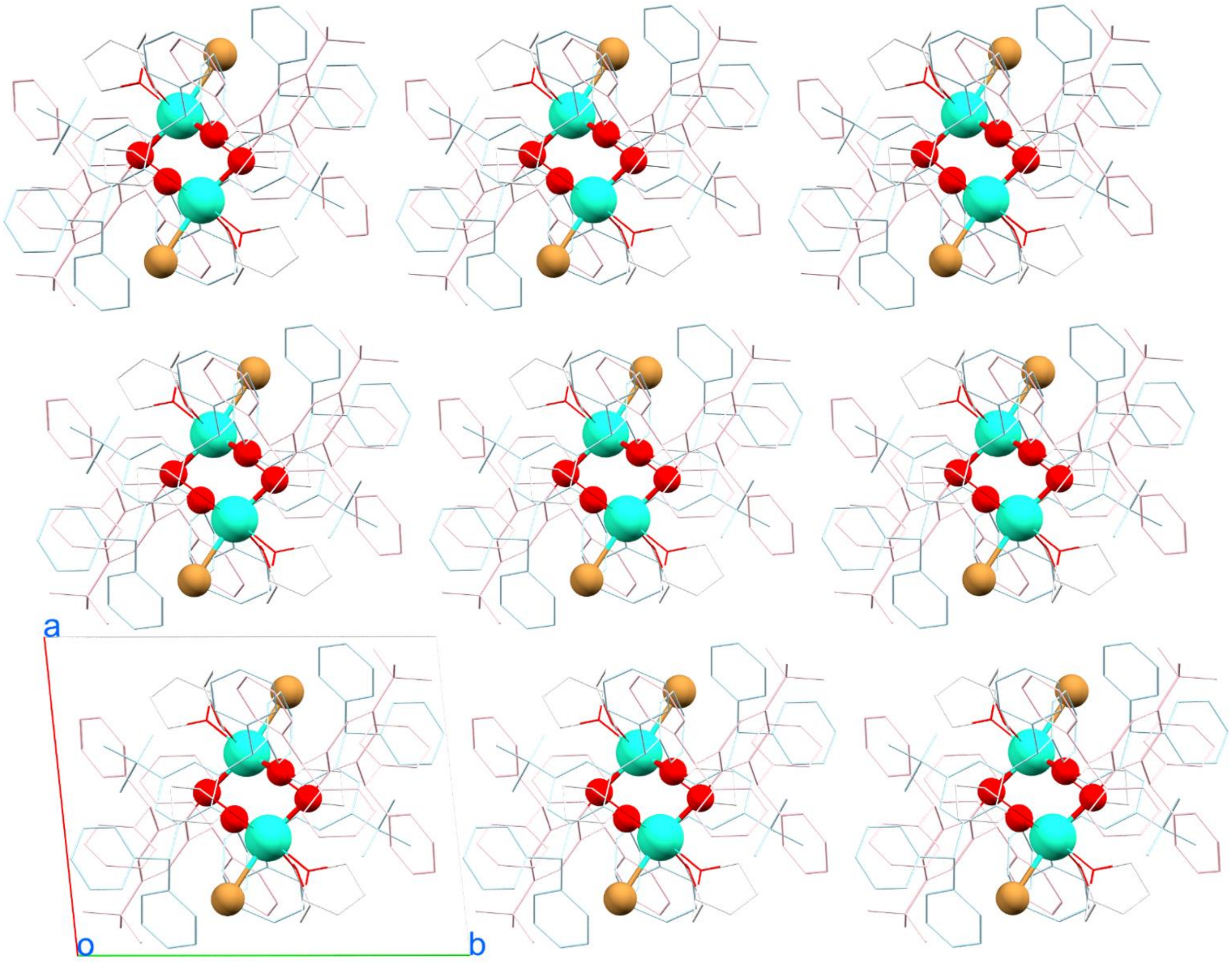

Figure S20. View of the crystal packing in [Dy(OPh*)$_2$(THF)$_3$Br] **2** along the c-axis (Dy cyan, Br brown, O red, C from THF in grey, C from OPh*$_1$ in blue, C from OPh*$_2$ in red). H atoms and lattice solvent are omitted for clarity.

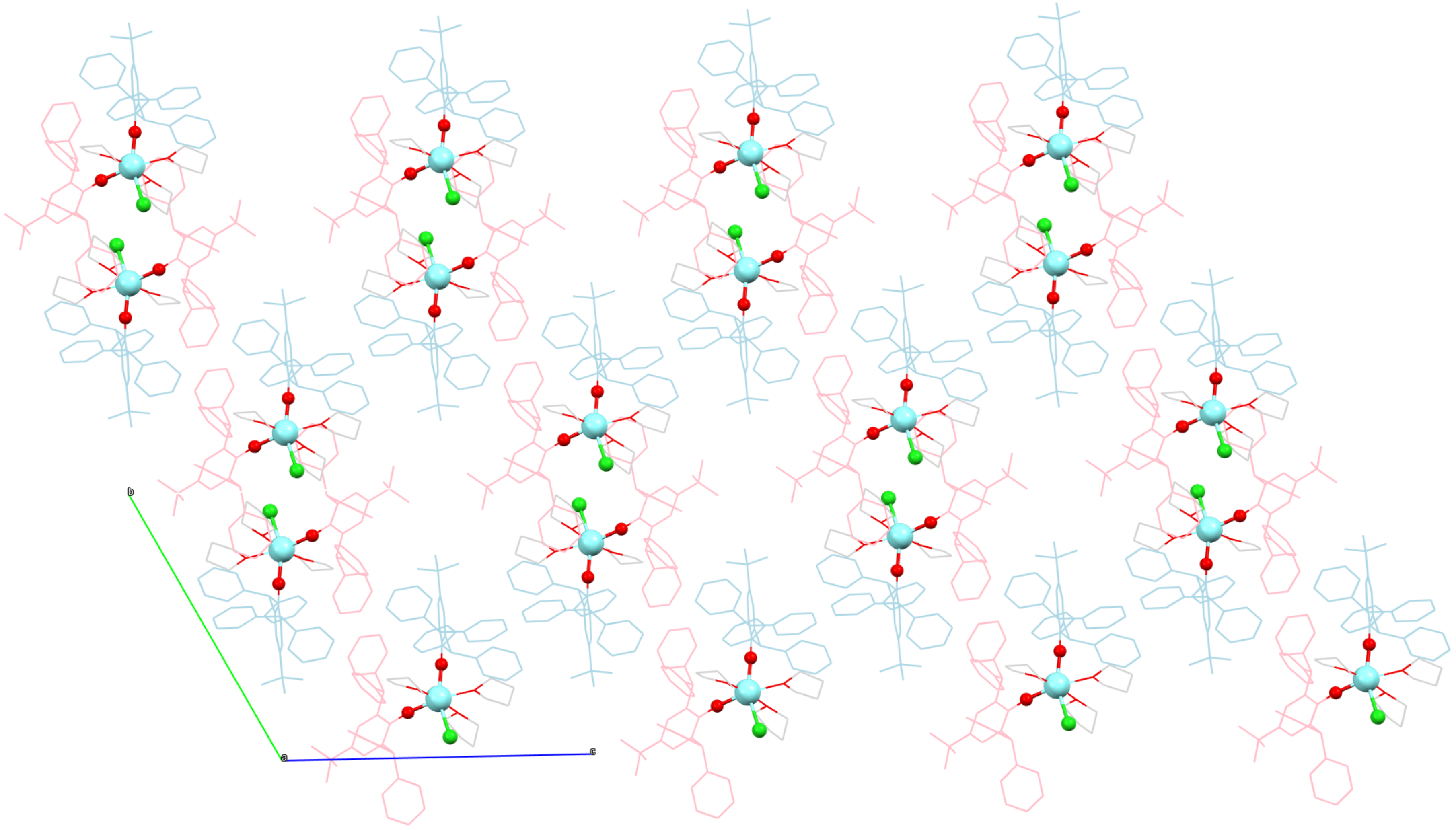

Figure S21. View of the crystal packing in [$Dy_{0.05}Y_{0.95}(OPh^*)_2(THF)_3Cl$] **5%Dy@1-Y** along the a-axis (Dy/Y cyan, Cl green, O red, C from THF in grey, C from OPh^*_1 in blue, C from OPh^*_2 in red). H atoms and lattice solvent are omitted for clarity.

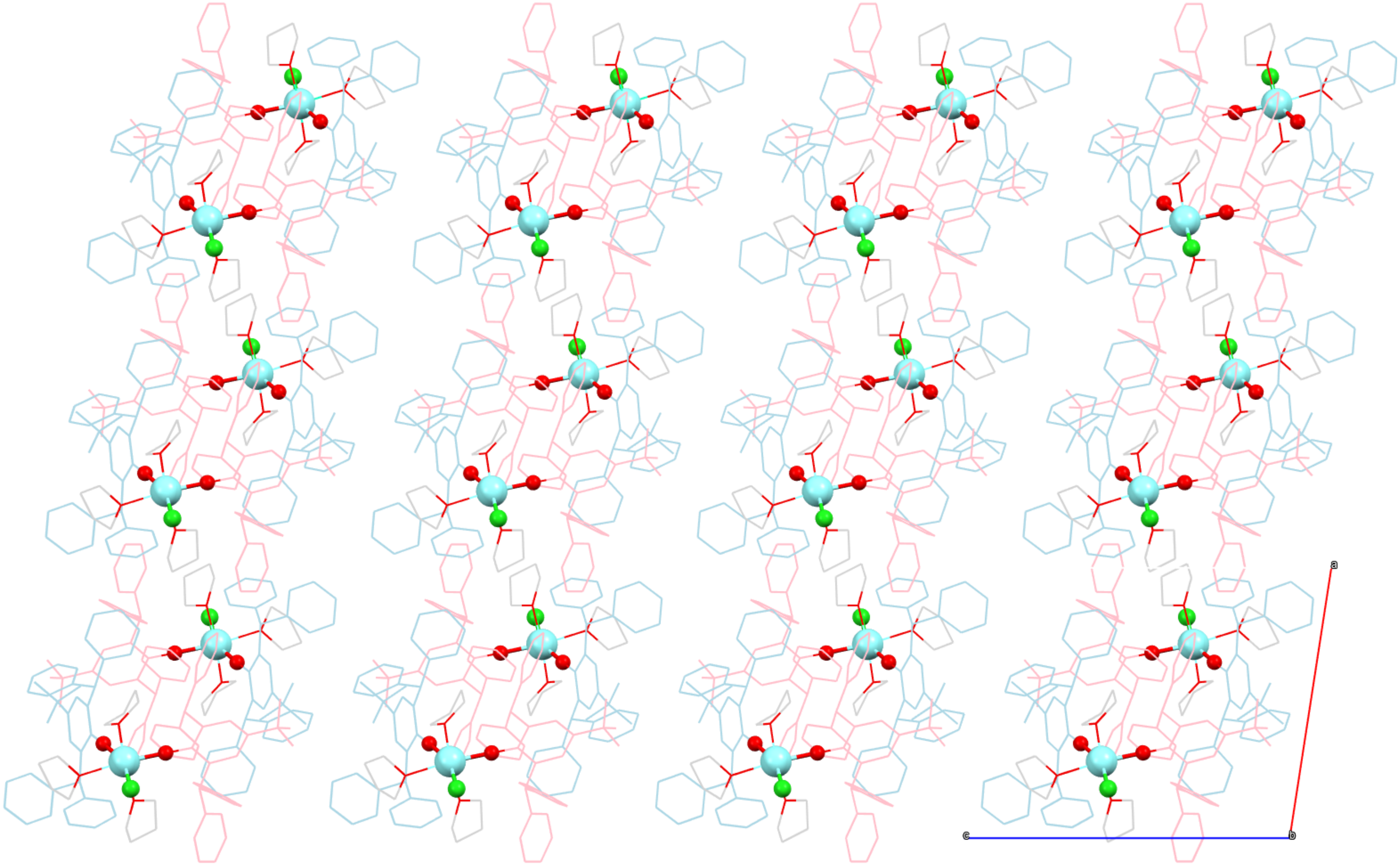

Figure S22. View of the crystal packing in [$Dy_{0.05}Y_{0.95}(OPh^*)_2(THF)_3Cl$] **5%Dy@1-Y** along the b-axis (Dy/Y cyan, Cl green, O red, C from THF in grey, C from OPh^*_1 in blue, C from OPh^*_2 in red). H atoms and lattice solvent are omitted for clarity.

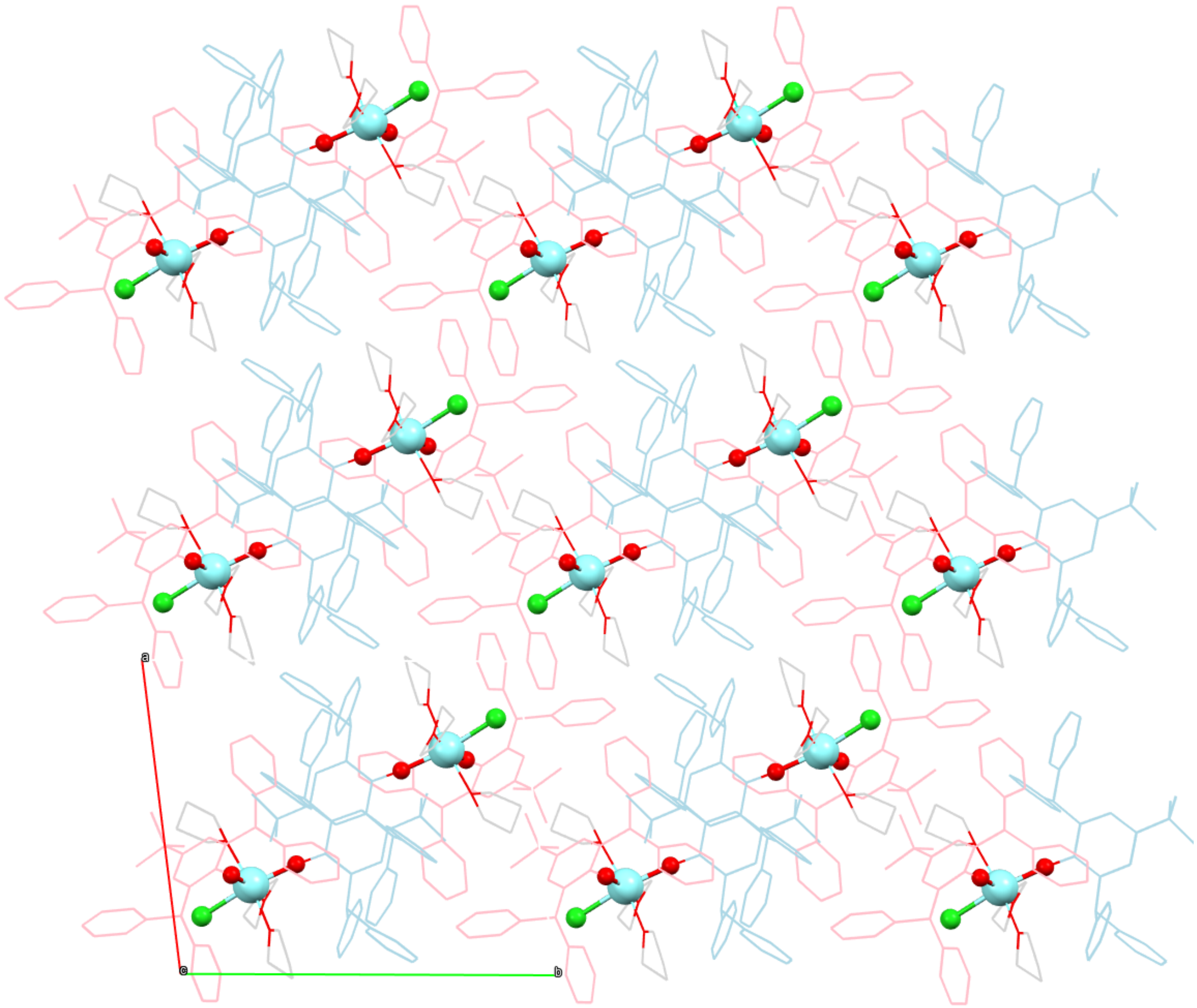

Figure S23. View of the crystal packing in [$Dy_{0.05}Y_{0.95}(OPh^*)_2(THF)_3Cl$] **5%Dy@1-Y** along the c-axis (Dy/Y cyan, Cl green, O red, C from THF in grey, C from OPh^*_1 in blue, C from OPh^*_2 in red). H atoms and lattice solvent are omitted for clarity.

6. Computational details

All first-principles complete active space self-consistent field spin-orbit (CASSCF-SO) calculations were performed on **1**, **2** and **5%Dy@1-Y** with the OpenMolcas[12] software package. The coordinates obtained from the single-crystal XRD study were used without optimisation, taking the major component in cases of disorder. The state-averaged CASSCF-SO/RASSCF/RASSI calculations were performed in the active space (9 electrons in 7 f-orbitals, CAS(9,7)) for the sextets (21 roots), quartet (224 roots) and doublet (490 roots) spin states of the $^6H_{15/2}$ term of Dy(III) ion. The RASSCF, RASSI and SINGLE_ANISO[13] modules were utilised to compute the g-tensors, crystal field parameters, energy and mixing of the m_J levels and interstate tunnelling probabilities. Quantisation was performed along the g_z axis of the effective S = ½ ground state. The basis sets for the *ab initio* calculations were chosen from the ANO-RCC library;[14,15] the Dy atom was treated with VTZP quality, the O, Cl, Br atoms with VDZP and others (C and H atoms) with VDZ quality with scalar relativistic effects accounted using the second-order Douglas-Kroll-Hess transformation.

Table S4. *Ab initio* results for the m_J multiplets of Dy(III) in **1**, **2** and **1** in **5%Dy@1-Y**.

	***Ab initio* Energy (cm^{-1})**	g_x	g_y	g_z	**g_z Angle (°)**	**Wavefunction[a]**
**1**	0	0.24	0.76	18.10	--	72%|±15/2⟩ + 25%|±11/2⟩ + 3%|±7/2⟩
	92.52	0.31	0.86	15.11	7.37	65%|±13/2⟩ + 30%|±9/2⟩ + 3%|±5/2⟩
	273.39	2.71	3.24	12.54	1.21	35%|±11/2⟩ + 31%|±7/2⟩ + 23%|±15/2⟩ + 3%|±3/2⟩ + 2%|∓1/2⟩ + 2%|±13/2⟩ + 2%|∓13/2⟩ + 2%|∓5/2⟩
	406.61	2.56	7.30	8.25	28.68	30%|±5/2⟩ + 29%|±9/2⟩ + 23%|±13/2⟩ + 6%|∓11/2⟩ + 6%|∓3/2⟩ + 2%|±11/2⟩ + 1%|±1/2⟩ + 1%|∓15/2⟩ + 1%|±7/2⟩
	527.21	1.38	4.13	12.61	79.05	29%|±7/2⟩ + 19%|±11/2⟩ + 15%|±3/2⟩ + 13%|∓9/2⟩ + 5%|±9/2⟩ + 3%|∓13/2⟩ + 3%|∓1/2⟩ + 3%|±5/2⟩ + 2%|±15/2⟩ + 2%|∓3/2⟩ + 2%|∓5/2⟩ + 2%|∓11/2⟩ + 1%|±13/2⟩
	585.84	0.31	1.88	16.74	81.76	20%|∓1/2⟩ + 11%|±3/2⟩ + 11%|±7/2⟩ + 10%|±5/2⟩ + 9%|∓5/2⟩ + 8%|±9/2⟩ + 7%|∓3/2⟩ + 7%|±11/2⟩ + 6%|±1/2⟩ + 4%|∓7/2⟩ + 2%|±13/2⟩ + 2%|∓9/2⟩ + 1%|∓11/2⟩
	614.80	0.87	2.59	16.95	83.32	21%|±5/2⟩ + 15%|∓1/2⟩ + 14%|±3/2⟩ + 14%|±1/2⟩ + 13%|∓3/2⟩ + 7%|±9/2⟩ + 6%|±7/2⟩ + 4%|∓7/2⟩ + 3%|∓5/2⟩ + 1%|∓9/2⟩ + 1%|±13/2⟩
	766.35	0.04	0.05	19.57	84.51	20%|±1/2⟩ + 19%|±3/2⟩ + 17%|∓1/2⟩ + 13%|±5/2⟩ + 10%|∓3/2⟩ + 8%|±7/2⟩ + 5%|∓5/2⟩ + 4%|±9/2⟩ + 2%|∓7/2⟩ + 1%|±11/2⟩
**2**	0.00	0.28	0.92	17.99	--	71%|±15/2⟩ + 22%|±11/2⟩ + 4%|±7/2⟩
	85.87	0.19	0.93	15.19	21.38	53%|±13/2⟩ + 30%|±9/2⟩ + 6%|±5/2⟩ + 5%|±7/2⟩ + 2%|±15/2⟩ + 2%|±11/2⟩
	261.92	2.94	3.32	12.42	5.12	26%|±7/2⟩ + 24%|±11/2⟩ + 21%|±15/2⟩ + 14%|±13/2⟩ + 6%|±3/2⟩ + 4%|±5/2⟩ + 2%|∓1/2⟩ + 1%|∓13/2⟩ + 1%|∓5/2⟩
	385.28	2.47	7.52	8.09	51.79	25%|±5/2⟩ + 18%|±11/2⟩ + 15%|±13/2⟩ + 15%|±9/2⟩ + 6%|±3/2⟩ + 4%|∓11/2⟩ + 4%|±1/2⟩ + 4%|∓3/2⟩ + 2%|∓13/2⟩ + 2%|∓5/2⟩ + 1%|±15/2⟩ + 1%|∓1/2⟩ + 1%|∓15/2⟩ + 1%|∓9/2⟩
	493.56	1.55	3.00	13.62	88.87	23%|±9/2⟩ + 11%|∓7/2⟩ + 10%|±3/2⟩ + 9%|±7/2⟩ + 8%|∓11/2⟩ + 7%|±11/2⟩ + 7%|5/2⟩ + 6%|∓9/2⟩ + 6%|±13/2⟩ + 4%|±1/2⟩ + 3%|∓3/2⟩ + 2%|∓1/2⟩ + 2%|∓13/2⟩ + 2%|±15/2⟩
	516.37	0.21	1.03	17.64	84.88	18%|±1/2⟩ + 15%|±3/2⟩ + 9%|±9/2⟩ + 8%|±7/2⟩ + 8%|±5/2⟩ + 8%|∓7/2⟩ + 6%|∓9/2⟩ + 6%|∓3/2⟩ + 5%|±11/2⟩ + 5%|∓1/2⟩ + 4%|∓11/2⟩ + 3%|∓5/2⟩ + 3%|±13/2⟩ + 2%|∓13/2⟩
	579.67	0.91	0.98	18.35	83.08	20%|∓1/2⟩ + 19%|±5/2⟩ + 15%|±1/2⟩ + 14%|±7/2⟩ + 13%|±3/2⟩ + 9%|∓3/2⟩ + 4%|∓5/2⟩ + 2%|∓7/2⟩ + 2%|±11/2⟩ + 1%|∓9/2⟩ + 1%|±9/2⟩
	680.36	0.04	0.11	19.34	77.29	23%|±3/2⟩ + 19%|±5/2⟩ + 18%|±1/2⟩ + 12%|±7/2⟩ + 9%|∓1/2⟩ + 7%|±9/2⟩ + 4%|∓3/2⟩ + 3%|±11/2⟩ + 2%|∓5/2⟩
**1 in 5%Dy@1-Y**	0.00	0.17	0.47	18.53	--	78%|±15/2⟩ + 20%|±11/2⟩ + 2%|±7/2⟩
	121.25	0.52	0.75	15.41	9.73	69%|±13/2⟩ + 25%|±9/2⟩ + 2%|±5/2⟩ + 2%|±7/2⟩
	310.71	2.33	2.73	12.76	4.58	44%|±11/2⟩ + 24%|±7/2⟩ + 19%|±15/2⟩ + 3%|±13/2⟩ + 2%|±3/2⟩ + 2%|±5/2⟩ + 2%|∓1/2⟩ + 1%|∓13/2⟩ + 1%|∓5/2⟩
	448.48	2.72	7.31	8.60	10.41	37%|±9/2⟩ + 20%|±13/2⟩ + 18%|±5/2⟩ + 6%|∓3/2⟩ + 5%|±11/2⟩ + 5%|∓11/2⟩ + 4%|±3/2⟩ + 2%|∓5/2⟩ + 1%|±1/2⟩
	531.01	0.80	2.18	18.14	88.67	22%|±7/2⟩ + 21%|∓1/2⟩ + 20%|±3/2⟩ + 18%|∓5/2⟩ + 8%|±11/2⟩ + 5%|∓9/2⟩ + 1%|∓13/2⟩
	548.83	1.87	4.76	12.70	86.80	24%|±7/2⟩ + 15%|±9/2⟩ +14%|±11/2⟩ + 11%|∓5/2⟩ + 10%|∓9/2⟩ + 9%|∓7/2⟩ +

						6%|±5/2⟩ + 3%|±3/2⟩ + 3%|∓11/2⟩ + 2%|∓13/2⟩ + 2%|±15/2⟩ + 2%|±13/2⟩ + 1%|∓3/2⟩
	698.83	0.31	0.35	17.82	88.19	26%|∓3/2⟩ + 22%|±5/2⟩ + 17%|∓1/2⟩ + 10%|±1/2⟩ + 8%|±3/2⟩ + 8%|±7/2⟩ + 4%|∓5/2⟩ + 3%|∓9/2⟩ + 1%|∓7/2⟩
	761.40	0.05	0.12	18.65	87.15	27%|±1/2⟩ + 22%|±3/2⟩ + 18%|∓1/2⟩ + 8%|±5/2⟩ + 6%|∓3/2⟩ + 6%|∓5/2⟩ + 5%|∓7/2⟩ + 3%|±7/2⟩ + 2%|±9/2⟩ + 1%|∓9/2⟩

[a] Contributions below 1% have been excluded for brevity

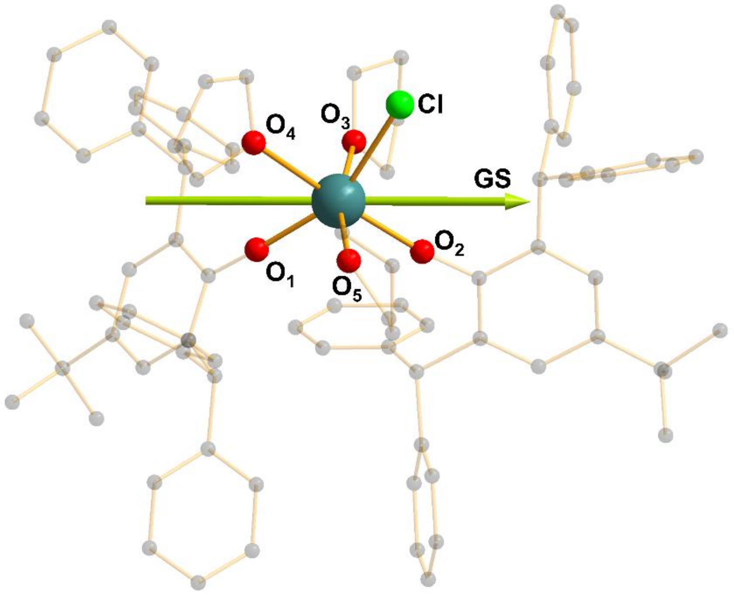

Figure S24. View of the molecular structure of $[Dy(OPh^*)_2(THF)_3Cl]$ (**1**) with the CASSCF-SO calculated magnetic axis of the ground state (g_z vector) shown as a green arrow (Dy cyan, Cl green, O red, C grey). H atoms are omitted for clarity.

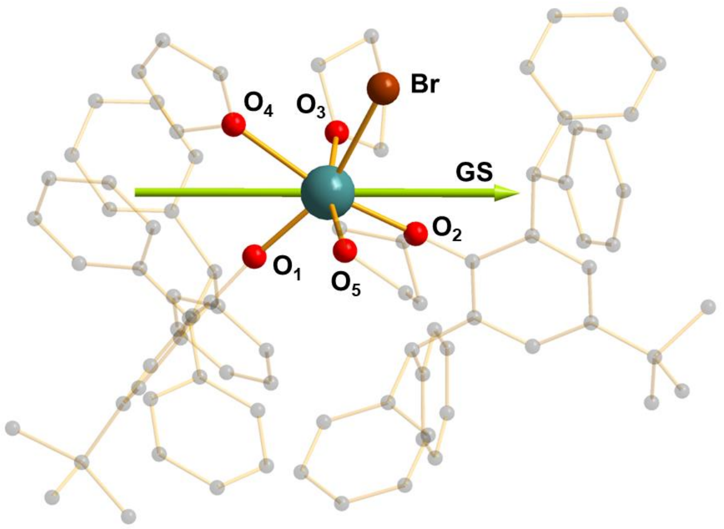

Figure S25. View of the molecular structure of [Dy(OPh*)$_2$(THF)$_3$Br] **2** with the CASSCF-SO calculated magnetic axis of the ground state (g_z vector) shown as a green arrow (Dy cyan, Br brown, O red, C grey). H atoms are omitted for clarity.

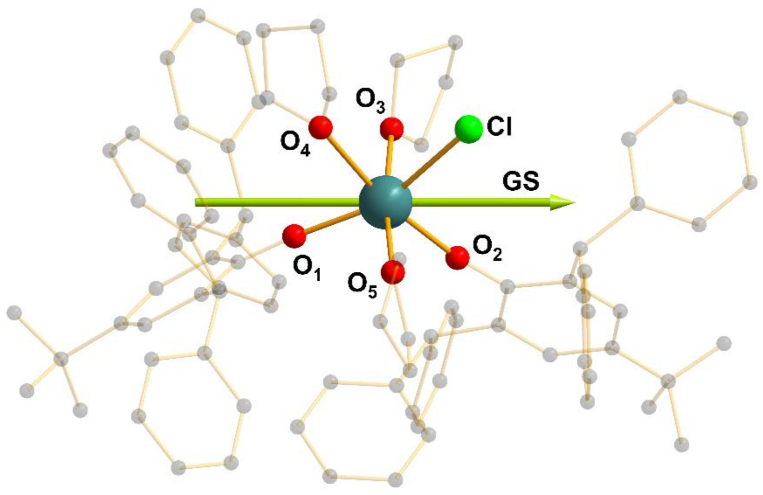

Figure S26. View of the molecular structure of [Dy(OPh*)$_2$(THF)$_3$Cl] in **5%Dy@1-Y** with the CASSCF-SO calculated magnetic axis of the ground state (g_z vector) shown as a green arrow (Dy cyan, Cl green, O red, C grey). H atoms are omitted for clarity.

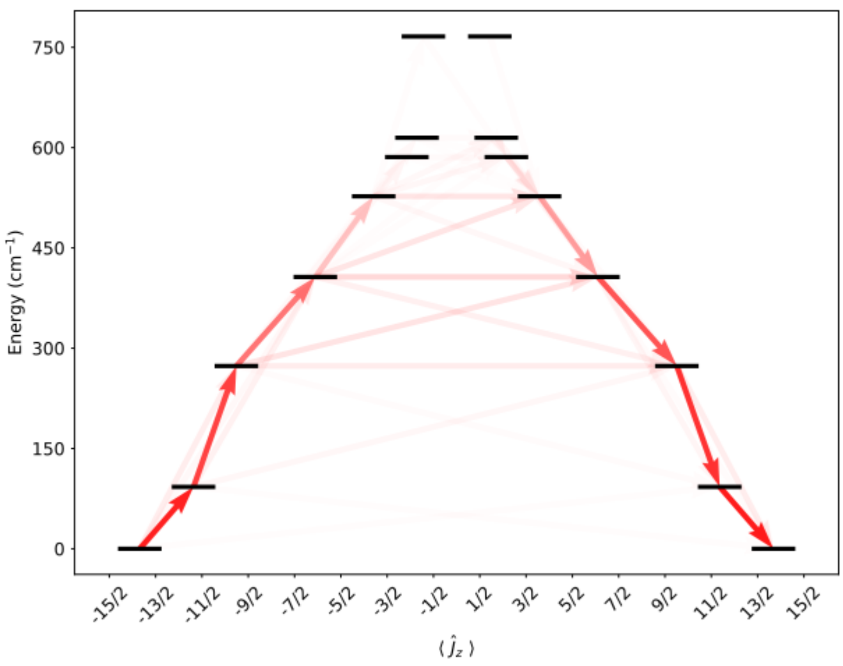

Figure S27. CASSCF-SO-calculated energy diagram of the ground-state multiplet for **1**. The arrows depict the transition propensities from each level.

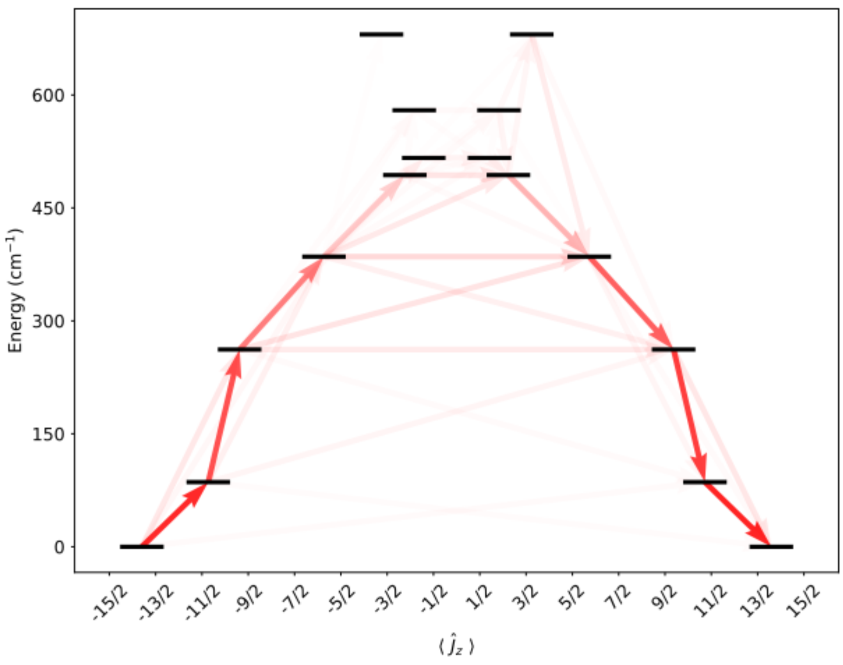

Figure S28. CASSCF-SO-calculated energy diagram of the ground-state multiplet for **2**. The arrows depict the transition propensities from each level.

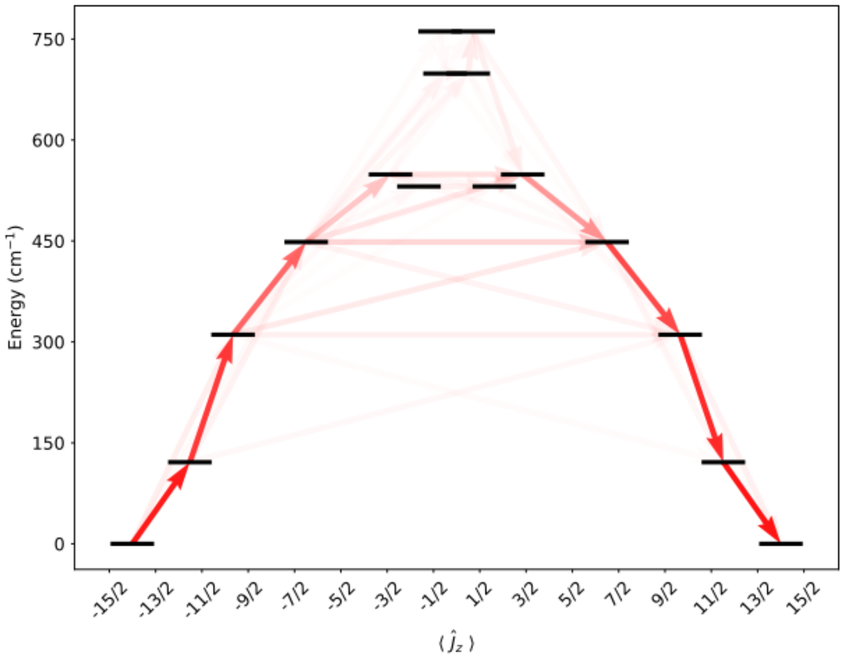

Figure S29. CASSCF-SO-calculated energy diagram of the ground-state multiplet for **1** in **5%Dy@1-Y**. The arrows depict the transition propensities from each level.

Table S5. CASSCF-SO computed LoProp and Mulliken charges per atom for **1** and **1** in **5%Dy@1-Y** (X = Cl) and **2** (X = Br).

		**Dy**	**X**	**O1**	**O2**	**O3**	**O4**	**O5**
	**1**	2.5149	-0.8595	-0.9620	-0.9648	-0.5553	-0.5590	-0.5540
**LoProp**	**2**	2.5025	-0.8501	-0.9573	-0.9611	-0.5524	-0.5617	-0.5795
	**1** in **5%Dy@1-Y**	2.5152	-0.8607	-0.9534	-0.9591	-0.5578	-0.5476	-0.5621
	**1**	1.9342	-0.7764	-1.1336	-1.1408	-0.9363	-0.9203	-0.9227
**Mulliken**	**2**	1.9114	-0.7537	-1.1393	-1.1416	-0.9274	-0.9295	-0.9293
	**1** in **5%Dy@1-Y**	1.9364	-0.7790	-1.1352	-1.1375	-0.9197	-0.9074	-0.9417

Table S6. CASSCF-SO Crystal Field Parameters (B_k^q) including operator equivalent factors for **1**, **2** and **5%Dy@1-Y**, with quantisation along the easy axis of the effective *S* = ½ ground state.

k	*q*	**1**	**2**	**5%Dy@1–Y**
2	–2	1.2884278699453	–0.73190763704109	0.39599838761591
2	–1	–0.96503753280784	–2.0121977475423	0.84824074770483
2	0	–3.2514443207264	–2.7466205166386	–3.4540526062397
2	1	0.17051759833708	0.69395873243651	0.13571663491623
2	2	2.8722219002037	2.5951228756262	1.7166061774300
4	–4	0.67639953946914E–2	–0.38643596587793E–2	–0.79191914989367E–3
4	–3	–0.85615225908704E–2	0.10611619312770E–1	–0.38441310504975E–1
4	–2	–0.75677813809332E–3	–0.11246350921337E–2	–0.16990901781967E–2
4	–1	0.12391577345170E–1	0.32222121095294E–1	–0.18069184295491E–1
4	0	0.17974817208477E–2	0.28921810387512E–3	0.11479452625858E–2
4	1	0.44276730546949E–2	–0.52362575980555E–2	0.14170328580948E–2
4	2	0.47257810784921E–1	0.43457684788721E–1	0.47645947149575E–1
4	3	0.11362549571901E–1	–0.51700548930357E–2	0.11939871141055E–2
4	4	–0.35622765120828E–1	–0.34561383598830E–1	–0.44279793271259E–1
6	–6	–0.11835590736735E–3	0.44347216751424E–4	–0.43052679490228E–4
6	–5	0.34789376727778E–3	0.58854040249588E–3	–0.15889207683259E–3
6	–4	0.64298835012237E–4	–0.61449276309436E–4	0.15258954160399E–5
6	–3	0.12145870434944E–3	0.16212235081174E–3	–0.67772727815115E–4
6	–2	–0.12552146051358E–4	0.23386259323272E–4	–0.83299970327219E–5
6	–1	0.99829770699642E–5	–0.35540949092315E–4	0.67126854582035E–4
6	0	–0.11483836135871E–4	–0.12384452407851E–4	–0.11559385965363E–4
6	1	–0.46931751273338E–4	0.15310320350689E–4	0.16976837320837E–5
6	2	0.17534850884897E–4	0.44492432822237E–5	0.60685793444206E–4
6	3	0.82245871441882E–5	0.59320113310949E–4	0.53561877993846E–4
6	4	0.10322387157941E–3	0.24084030618719E–4	0.10420671493506E–3
6	5	0.27588133575647E–3	–0.23128626451605E–3	0.70021842591204E–4
6	6	0.23393455429089E–3	0.20976498763587E–3	0.31332456163576E–3

7. EPR spectroscopy

X-Band EPR spectra were collected on an EMX Plus Bruker Spectrometer at 9.38 GHz, fitted with Stinger helium gas cryosystem or liquid helium cryosystem (**5%Dy@1-Y**). Spectra were recorded at 5.0 K for frozen solutions of **1** and **2**, 6.9 K for powders of **1** and **2** and 17 K for the powder of **5%Dy@1-Y**. Crystalline samples of **1**, **2** and **5%Dy@1-Y** were finely ground and transferred to 4 mm quartz tubes in a glovebox, where they were covered in powdered eicosane and gently heated to restrain the sample before being sealed under vacuum. Initial spectra collected without restraining the sample showed immediate alignment with the field. Solutions of **1** and **2** in 9:1 toluene/hexane (10 mM) were prepared in a glovebox, transferred to 4 mm quartz tubes, frozen in liquid nitrogen and sealed under vacuum. The solution samples were stored in liquid nitrogen until measurement. Powder spectra were collected at 15 dB attenuation with 1 mT modulation amplitude. After collecting the first spectrum, the powder sample rotated by approximately 90 degrees to check for polycrystalline effects. Frozen solution spectra were collected at 15 dB attenuation with 0.5 mT modulation amplitude. The field was corrected using a strong pitch sample (g = 2.0028).

Spectral simulations were performed using the pepper function in EasySpin 6.0.0-dev.30 to give the parameters given in Table S7.[16] An S = ½ model was used with anisotropic g-values. In the powder spectra the g_x feature was not observed ($g < 0.4$) and was fixed to the CASSCF value for the purpose of simulation. In the frozen solution spectra, the g_x and g_y features were not clearly observed ($g < 0.4$ or broadened into the baseline) and were fixed to 0.15 for the purpose of simulation. Minor polycrystallinity of the **5%Dy@1-Y** was observed, as indicated by differences

in the spectra of two rotations of the powder sample, resulting in higher uncertainty in modelling the g_y feature. Spectral lines are broadened as a result of local distributions in the internal field due to electron-nuclear hyperfine interactions and electron spin-spin dipolar interactions (particularly in undiluted samples). Broadening was modelled by anisotropic *g*-strains, which should be considered a phenomenological parameter accounting for the aforementioned effects, the sharp HF-EPR spectra indicate the true *g*-strains are minimal. In the frozen solution spectra and powder spectrum of **5%Dy@1-Y**, hyperfine coupling to Dy nuclei (18.9% ^{161}Dy with I = 5/2, 24.9% ^{163}Dy with I = 5/2) could be resolved on the g_z feature. In the simulations of these spectra, an A_z hyperfine coupling to Dy was added to the model ($A_x = A_y = 0$). To convert from the S = ½ coupling value to the full ion J = 15/2 picture, A_z values were divided by 15.

High-field/frequency EPR (HF-EPR) measurements were performed on an unconstrained powder sample of **5%Dy@1-Y** at frequencies of 128.0 GHz and 257.2 GHz using the homodyne broadband transmission spectrometer of the Electron Magnetic Resonance facility at the US National High Magnetic Field Laboratory. This is a home-built instrument in which microwaves are propagated to and from the sample in cylindrical lightpipes.[17] High-frequencies are generated in the 24 to 660 GHz range via a tunable phase-locked solid-state source with a base frequency of 13.1 ± 1 GHz, followed by a solid-state harmonic multiplier chain (both Virginia Diodes Inc., Charlottesville, VA). A 15 T superconducting magnet system equipped with a variable-flow helium cryostat (both Oxford Instruments plc, U.K.) enabled measurements in the 5 to 300 K range. The field-modulated EPR intensity ($\mathrm{d}I/\mathrm{d}B$) was collected via a phase-sensitive homodyne detection scheme using a helium-cooled InSb bolometer (QMC Ltd., Cardiff, UK) and a lock-in amplifier.

Spectral simulations were performed on the resultant spectra using the EasySpin software package.[16]

HF-EPR spectra were recorded at 128.0 GHz (Fig. S36) and 257.2 GHz (Fig. 3, main article). Clear evidence for partial alignment of crystallites in the loose powder can be seen from the asymmetric lineshape of the stronger central transition which, assuming a dominant easy-axis magnetoanisotropy, should otherwise exhibit an antisymmetric profile with equal and opposite deviations above and below the baseline. As noted below, this can be corrected for in the simulations and has the added advantage that it significantly increases the sensitivity of the measurements to the parallel components of the spectra. The stronger central transition can be attributed to the non-magnetic Dy nucler isotopes with ~56% natural abundance. Meanwhile, the weaker features either side of the central transition can be attributed to the two I = 5/2 isotopes, ^{161}Dy and ^{163}Dy, with ~44% natural abundance.

The simulations were generated by adding spectra both with and without hyperfine interactions in the expected ratio of natural abundances for the magnetic and non-magnetic isotopes. Even though EasySpin can account for differences in hyperfine coupling for different isotopes, we assumed identical axial hyperfine coupling constants (A_z) for the two I = 5/2 species, as their individual splitting patterns were not resolved at high-fields (neither in the experiments nor the simulations assuming inequivalent hyperfine coupling strengths), presumably due to increased g-strain. The employed 4.4 mT peak-to-peak linewidth used in the simulations results in resolution of four of the expected $2I + 1 = 6$ hyperfine resonances, i.e., the two outermost on either side of the central transition; the two innermost resonances are not resolved from the central resonance. Finally, a mixture of pure absorption and

first derivative intensity was employed to capture the asymmetric lineshape caused by alignment of the powder. The simulations reproduce most aspects of the experimental spectra, including the relative intensities of the central and satellite hyperfine features. All four resolved hyperfine peaks are just about discernable above the noise in the 128 GHz spectra, whereas the two hyperfine features on the high-feld side of the central transition have merged into a single broad peak in the 257.2 GHz spectra, again presumably due to increased *g*-strain. The spin-Hamiltonian parameters deduced from the simulations are given in Table S7.

Table S7. EPR simulation experimental and simulation parameters and CASSCF *g*-values

	Freq. / GHz	***T* / K**	g_z	g_y	g_x	***g-strain**$_z$*	***g-strain**$_y$*	**p-p linewidth / mT**	**A_z (S = ½) / MHz**	**A (*J* = 15/2) / MHz**
**1** powder	9.379332 (rot 1)	6.9	18.6	0.83	-	19.5	0.64	-	-	-
	9.380314 (rot 2)									
**1** 10 mM frozen solution	9.382057	5.0	18.45	-	-	1.8	-	-	2120	141.3
**1** CASSCF	-	-	18.12	0.76	0.24	-	-	-	-	-
**5%Dy@1-Y** powder	9.376244 (rot 1)	17	19.44	0.503	-	2.2	0.062	-	2220	148
	9.372826 (rot 2)									
**5%Dy@1-Y** powder	128	5	18.80	-	-	-	-	4.4	2100[a]	140[a]
**5%Dy@1-Y** powder	257.2	5	19.00	-	-	-	-	4.4	2100	140
**2** powder	9.385538 (rot 1)	6.9	18.9	0.82	-	17.5	0.528	-	-	-
	9.388882 (rot 2)									
**2** 10 mM frozen solution	9.383355	5.0	17.60	-	-	3.3	-	-	2080	138.7
**2** CASSCF	-	-	17.95	0.95	0.28	-	-	-	-	-

[a] Fixed from 257.2 GHz spectrum

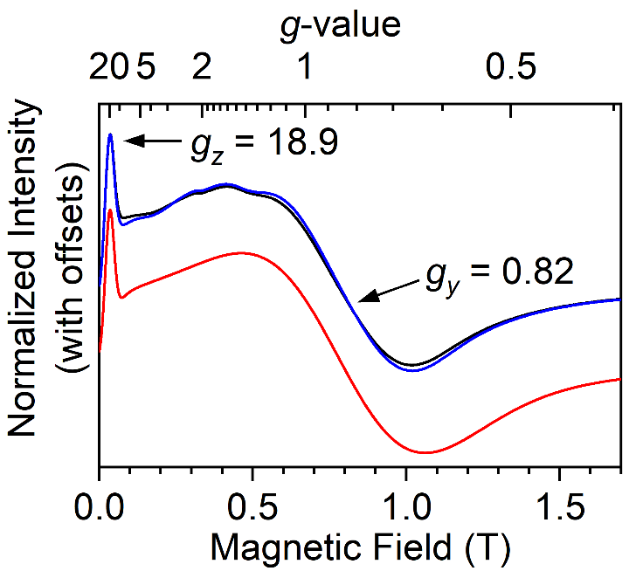

Figure S30. Continuous wave X-band (ν = 9.385538 and 9.388882 GHz for **2**) EPR spectra of a powder of **2**. Colour code: experimental rotation 1 (black), experimental rotation 2 (blue) and simulation (red) with parameters given in Table S7.

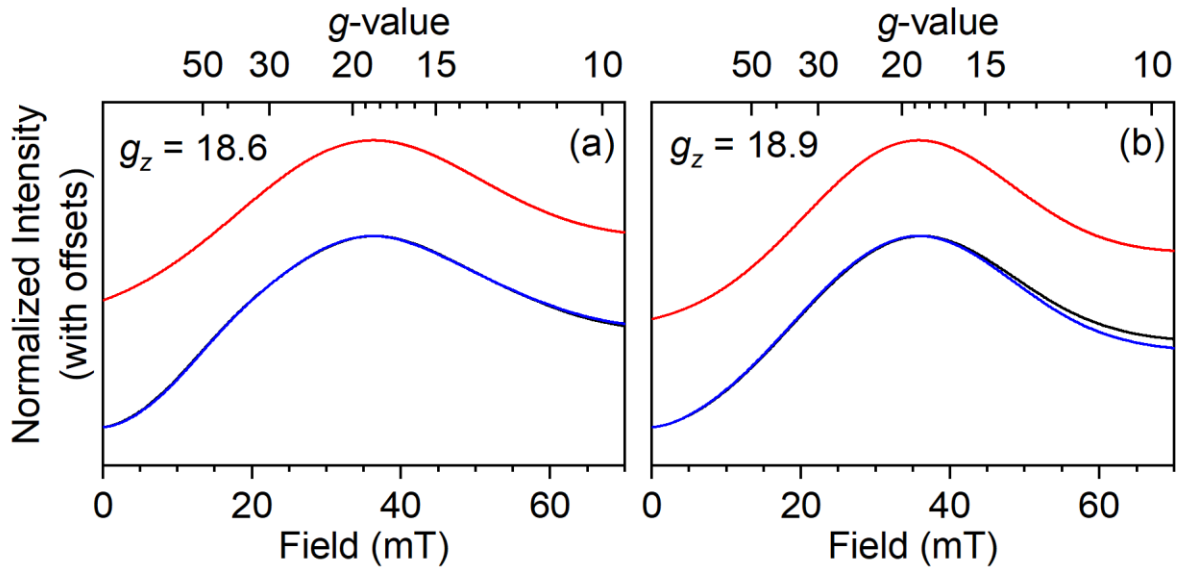

Figure S31. Continuous wave X-band (ν = 9.379332 and 9.380314 GHz for **1**, 9.385538 and 9.388882 GHz for **2**) EPR spectra at low field of powders of (a) **1** and (b) **2**. Colour code: experimental rotation 1 (black), experimental rotation 2 (blue) and simulation (red) with parameters given in Table S7.

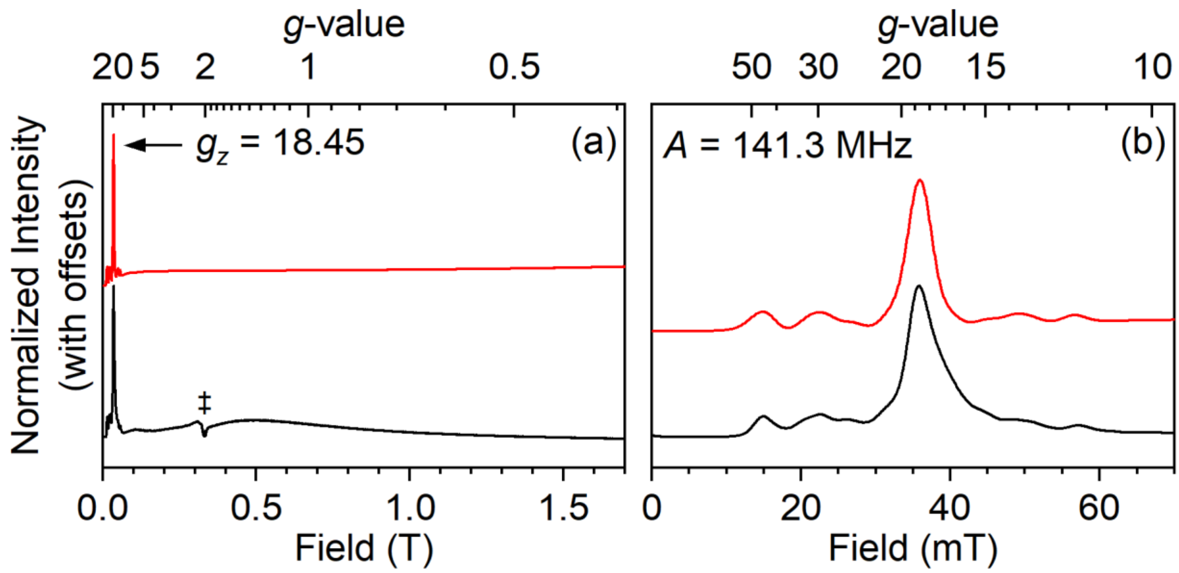

Figure S32. Continuous wave X-band (ν = 9.382057 GHz) EPR spectra of 10 mM frozen solutions in 9:1 toluene/hexane of **1** showing (a) full field range and (b) low field region. Colour code: experimental spectrum (black), simulation (red) with parameters given in Table S7. The cavity background signal (‡) is prominent because of the weak signal of the dilute solution.

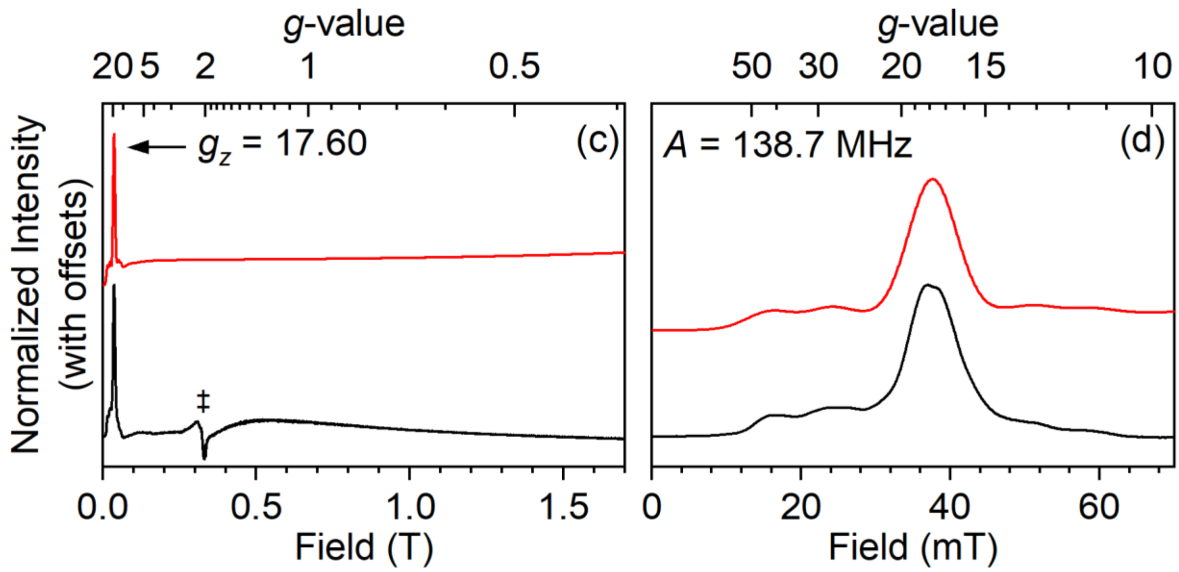

Figure S33. Continuous wave X-band (ν = 9.383355 GHz) EPR spectra of 10 mM frozen solutions in 9:1 toluene/hexane of **2** showing (a) full field range and (b) low field region. Colour code: experimental spectrum (black), simulation (red) with

parameters given in Table S7. The cavity background signal (‡) is prominent because of the weak signal of the dilute solution.

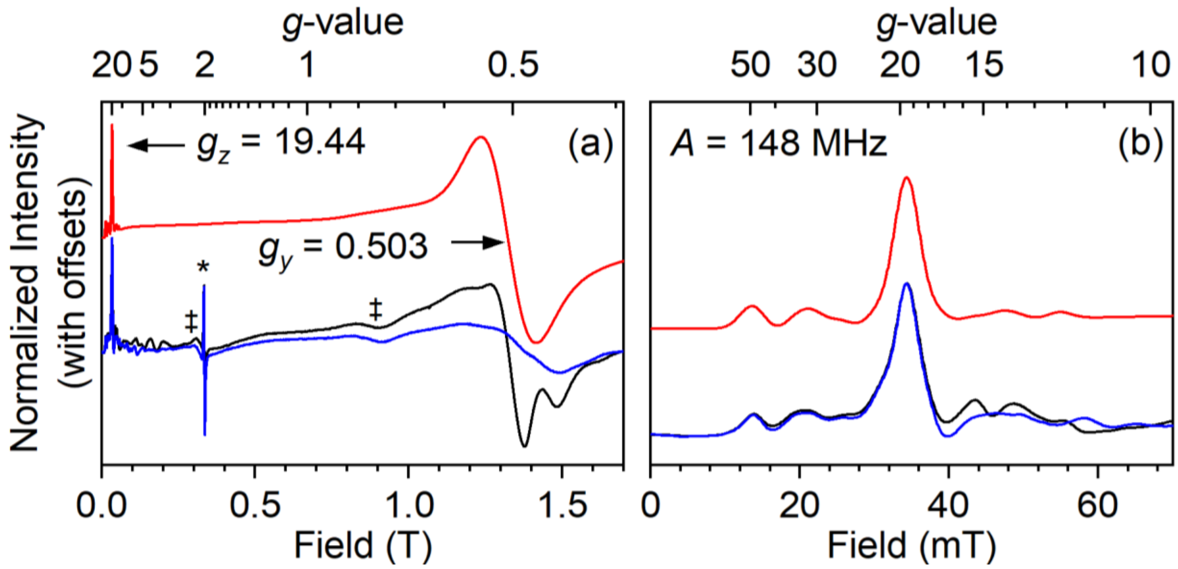

Figure S34. Continuous wave X-band (ν = 9.376244 and 9.372826 GHz) EPR spectra of **5%Dy@1-Y** powder (a) and showing the low field region (b). Colour code: experimental rotation 1 (black), experimental rotation 2 (blue) and simulation (red) with parameters given in Table S7. Cavity background signals (‡) and a small radical impurity (*) are prominent because of the weak signal of the diluted compound.

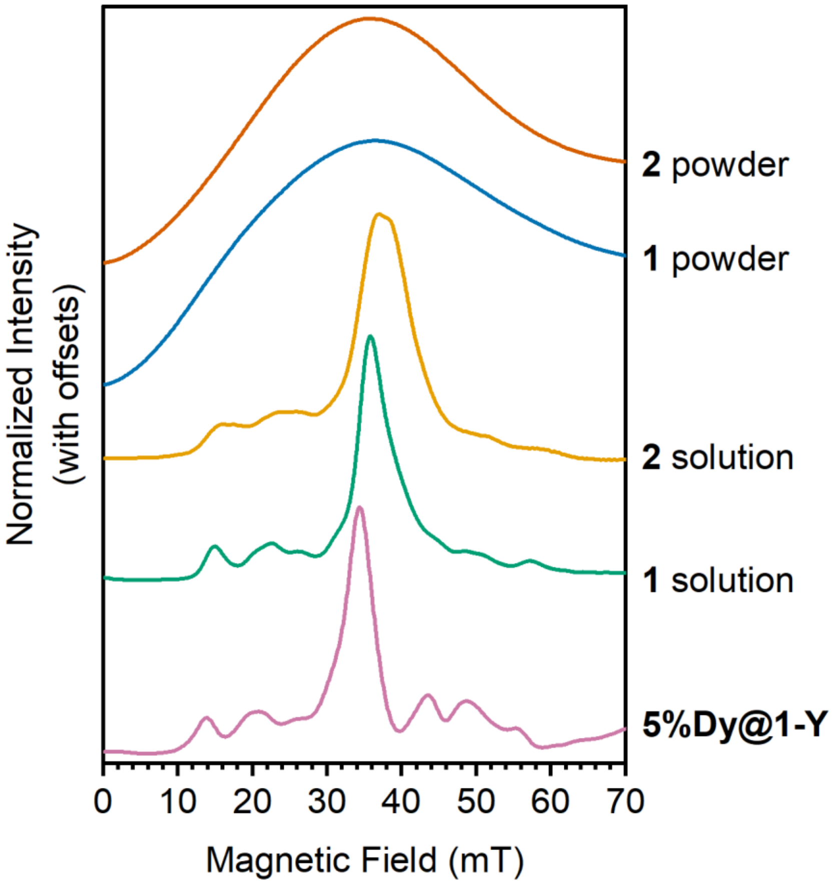

Figure S35. Continuous wave X-band (v = 9.37–9.39 GHz) EPR low field feature for powder **1** (blue), powder **2** (red), 10 mM 9:1 toluene/hexane solution of **1** (green), 10 mM 9:1 toluene/hexane solution of **2** (gold) and powder **5%Dy@1-Y** (pink).

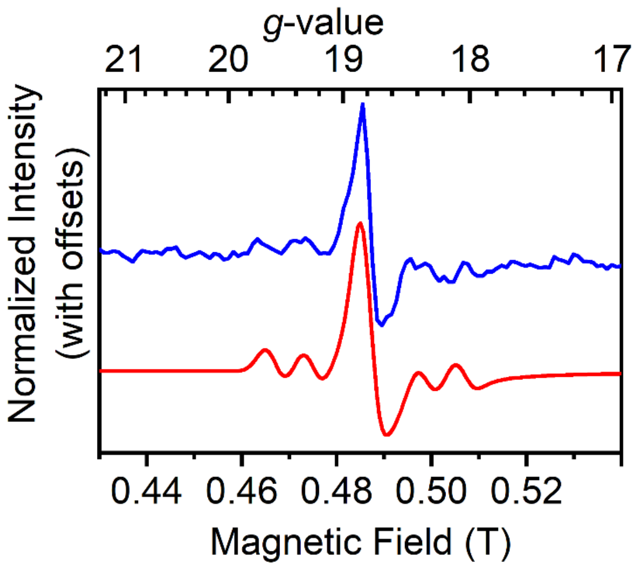

Figure S36. High-field powder EPR spectra of **5%Dy@1-Y** at 128 GHz showing experimental spectra at 5 K (blue) and simulation (red).

8. Magnetism

Magnetic measurements were performed on a Quantum Design MPMS-XL7 (**1** magnetisation, **2** dc measurements) or MPMS3 (**5%Dy@1-Y,** solution samples, **1** and **2** ac measurements, **1** susceptibility) superconducting quantum interference device (SQUID) magnetometer. Crystalline samples of **1** (37.0 mg for magnetisation, 40.0 mg for other measurements), **2** (38.0 mg) or **5%Dy@1-Y** (70.0 mg) were crushed with a mortar and pestle under an inert atmosphere, and then loaded into a borosilicate glass NMR tube along with 25.2, 30.0, 22.0 or 30.8 mg, respectively, of powdered eicosane. The eicosane was melted by heating the tube gently with a low-power heat gun in order to immobilize the crystallites. For solution samples, 29.05 or 14.5 mg of **1** were loaded into a borosilicate glass NMR tube and then dissolved in 100 μL of dry, degassed 9:1 toluene:hexane to form 200 and 100 mM solutions of **1**. The solutions were flash-frozen in liquid N_2 and all tubes were evacuated and flame-sealed to a length of ca. 5 cm. The NMR tube was then mounted in the centre of a drinking straw using friction by wrapping it with Kapton tape, and the straw was then fixed to the end of the sample rod. Solution samples were again frozen in liquid N_2 prior to loading in the SQUID, and were rapidly cooled in zero field after loading. The measurements were corrected for the diamagnetism of the straw, borosilicate tube and eicosane using calibrated blanks, and the intrinsic diamagnetism of the sample was calculated as $-0.5\times Mw(g\ mol^{-1})\times 10^{-6}\ cm^3\ mol^{-1}$. MPMS3 susceptibility measurements were corrected for the shape of the sample using the MPMS3 Sample Geometry Simulator. Data on **5%Dy@1-Y** was calculated per mol of Dy, using a molecular weight with one molecule of pentane. Data for **2** was calculated for the desolvated sample, without hexane. Data for **1** was calculated with one diethyl ether molecule. AC data and relaxation rates were fitted in CC-FIT2.[18,19]

DC hysteresis measurements were collected by magnetising the samples at 7 T and sweeping the field through zero towards -7 T, and then sweeping the field back through zero towards 7 T, at a fixed temperature. The average field sweep rate was 16(2) Oe s^{-1} for |H| < 0.6 T, 28(5) Oe s^{-1} for 0.6 < |H| < 1 T, 45(3) Oe s^{-1} for 1 < |H| < 2 T, and 84(3) Oe s^{-1} for 2 < |H| < 7 T, giving an overall average sweep rate of 52(3) Oe s^{-1}.

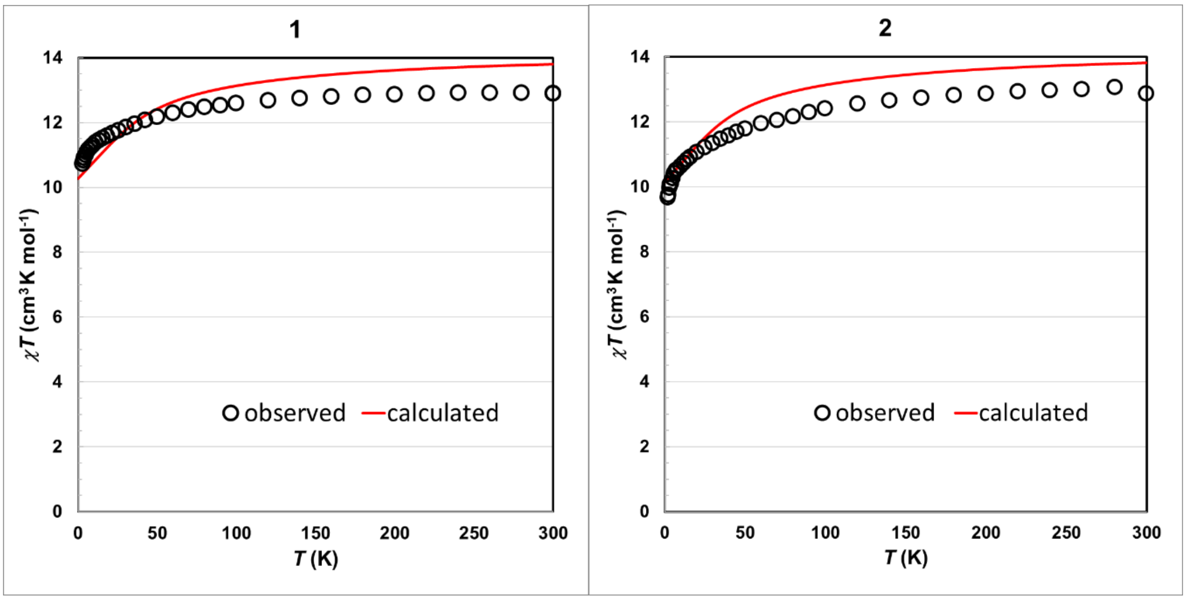

Figure S37. Experimental (black circles) and the CASSCF-SO calculated (red line) temperature dependence of the χT product between 0 and 300 K **1** (left) and **2** (right). χ is defined as magnetic susceptibility equal to M/H per mole. The experimental χT product is observed at 0.1 T field. The χT products at 300, 100 and 2 K are 12.90, 12.60, 10.46 cm^3 K mol^{-1} for **1**, and 12.87, 12.41 and 9.67 cm^3 K mol^{-1} for **2**.

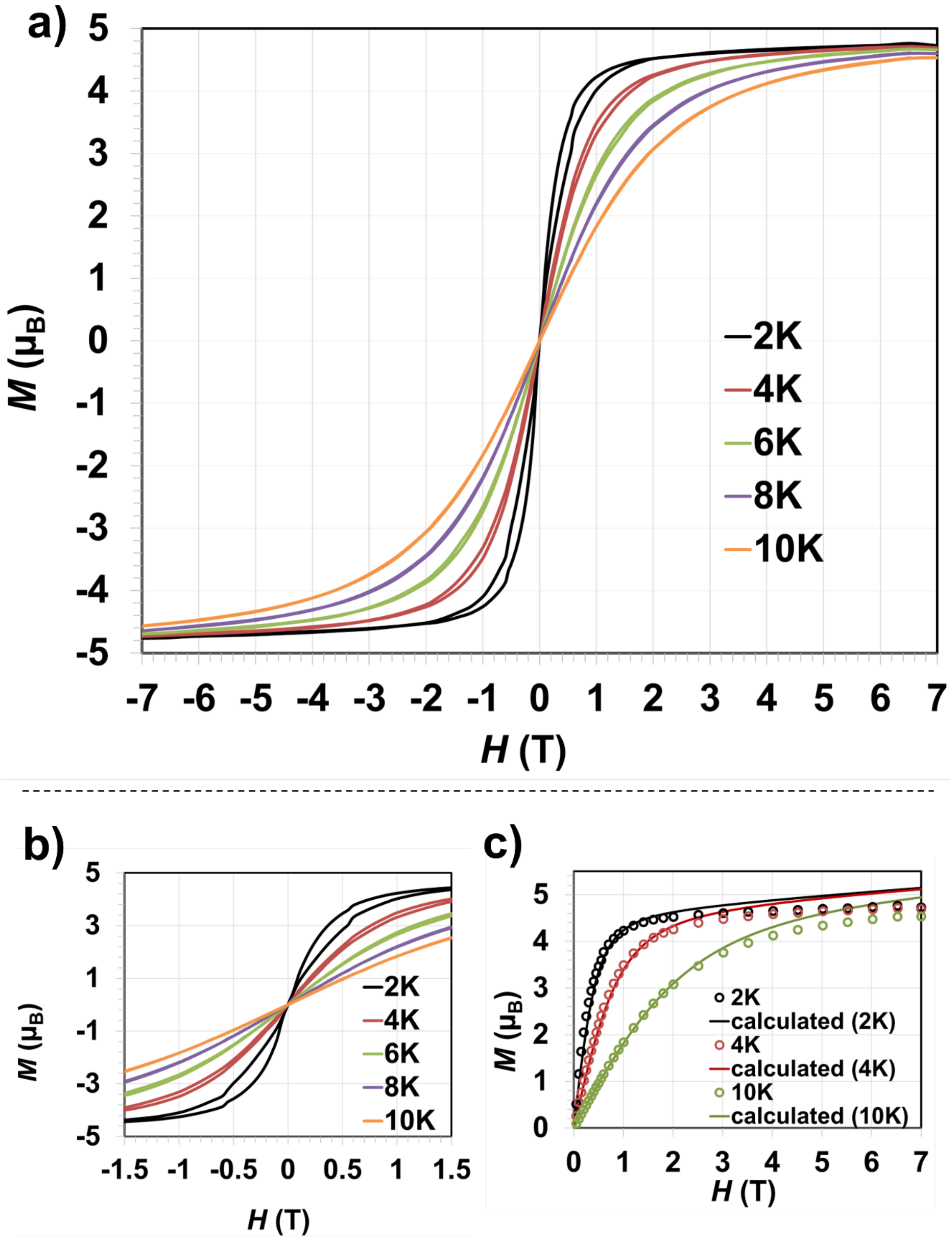

Figure S38. Field dependence of the magnetisation for **1**. **a)** M vs H data between 2 and 10 K have been measured at 17 mT/min to obtain magnetisation hysteresis curves between -7 and 7 T. **b)** a zoom of the hysteresis curves between -1.5 and 1.5 T. **c)** M vs H data between 2-10 K and 0-7 T.

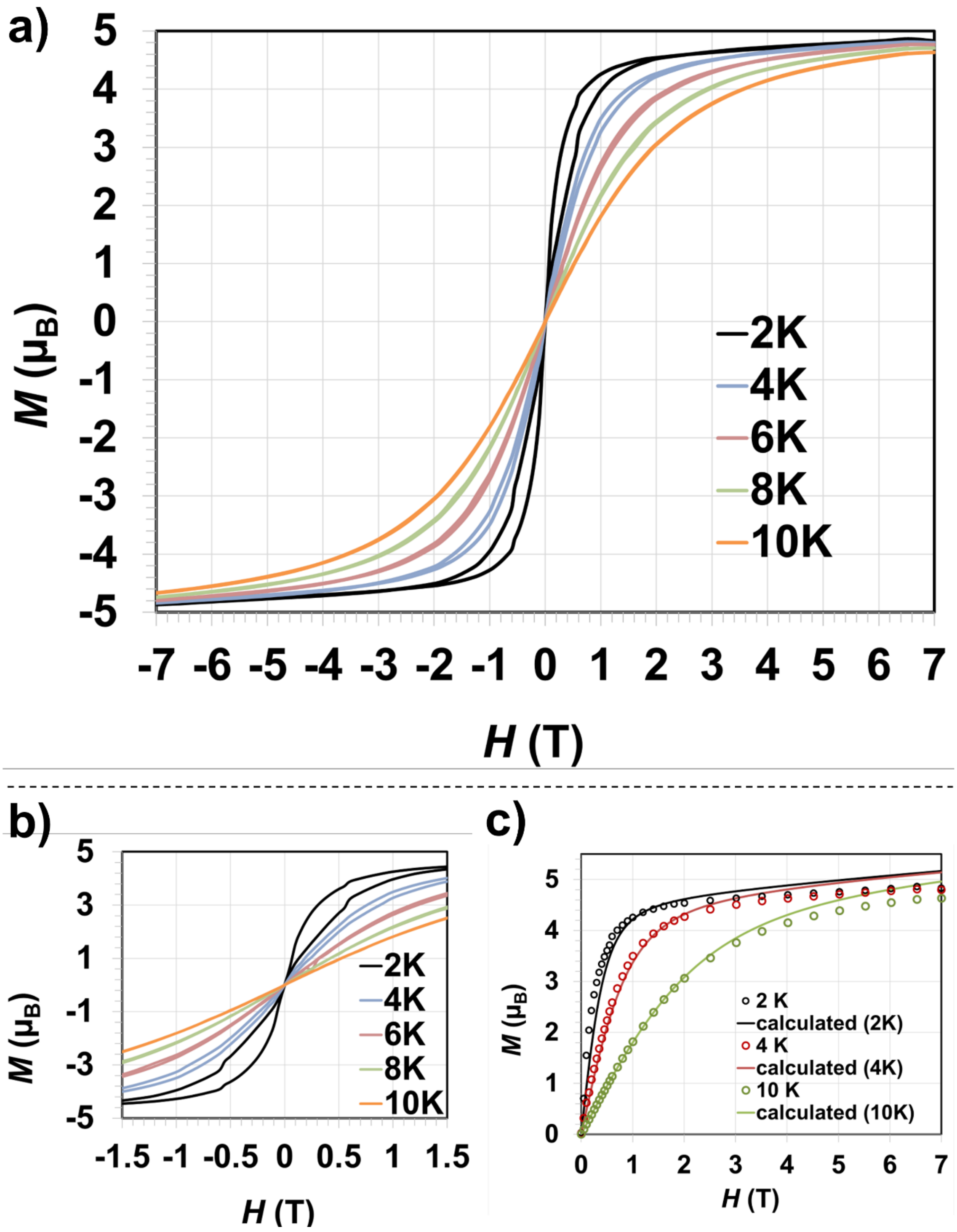

Figure S39. Field dependence of the magnetisation for **2**. **a)** M vs H data between 2 and 10 K have been measured at 17 mT/min to obtain magnetisation hysteresis curves between -7 and 7 T. **b)** a zoom of the hysteresis curves between -1.5 and 1.5 T. **c)** M vs H data between 2-10 K and 0-7 T.

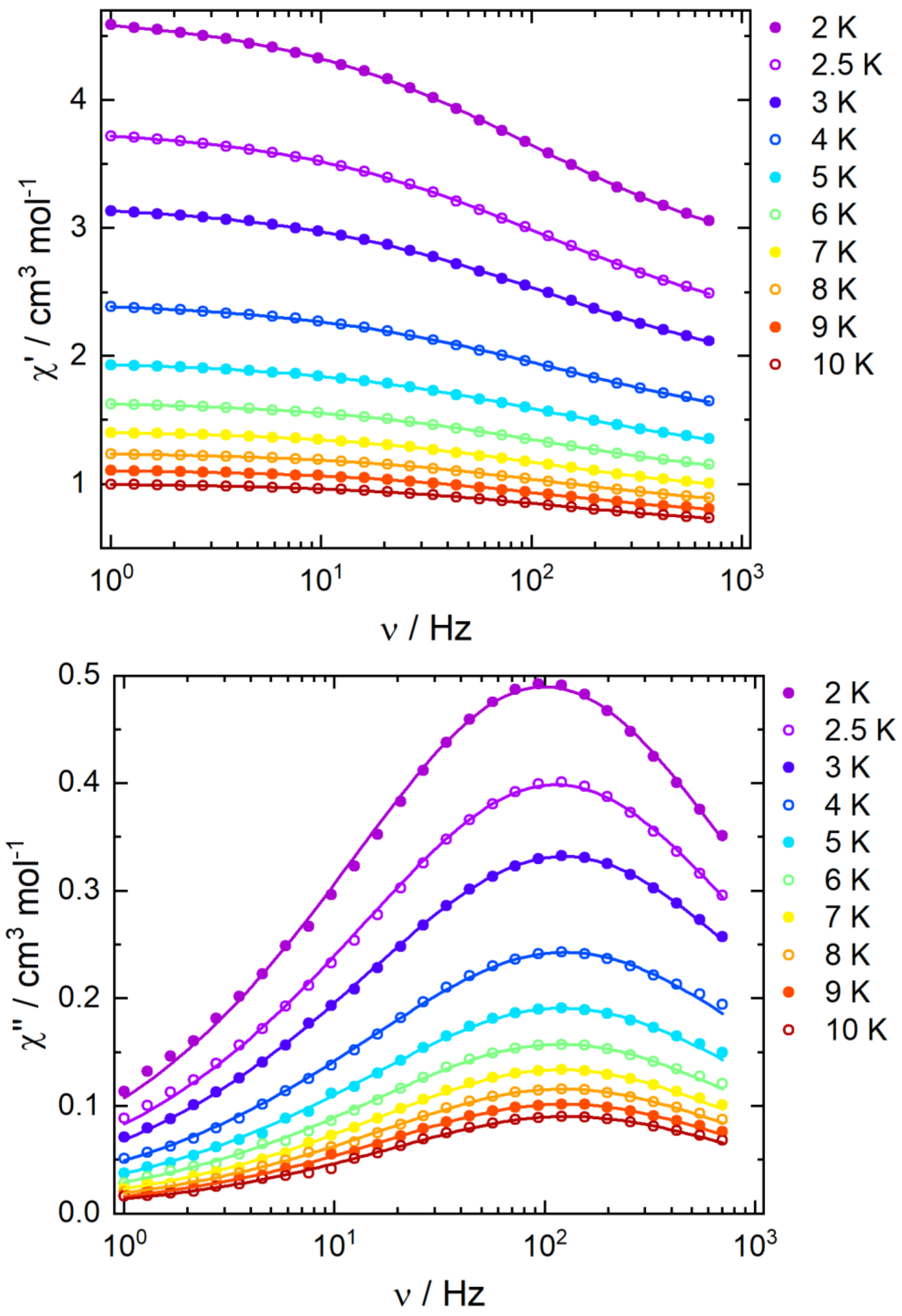

Figure S40. AC frequency dependences of the real (χ', top) and imaginary (χ'', bottom) parts of the AC susceptibility for **1**, between 2 and 10 K for ac frequencies between 1 and 700 Hz (in a zero-dc field). Solid lines are the generalised Debye fit of the ac data used to extract the temperature dependence of the relaxation time.

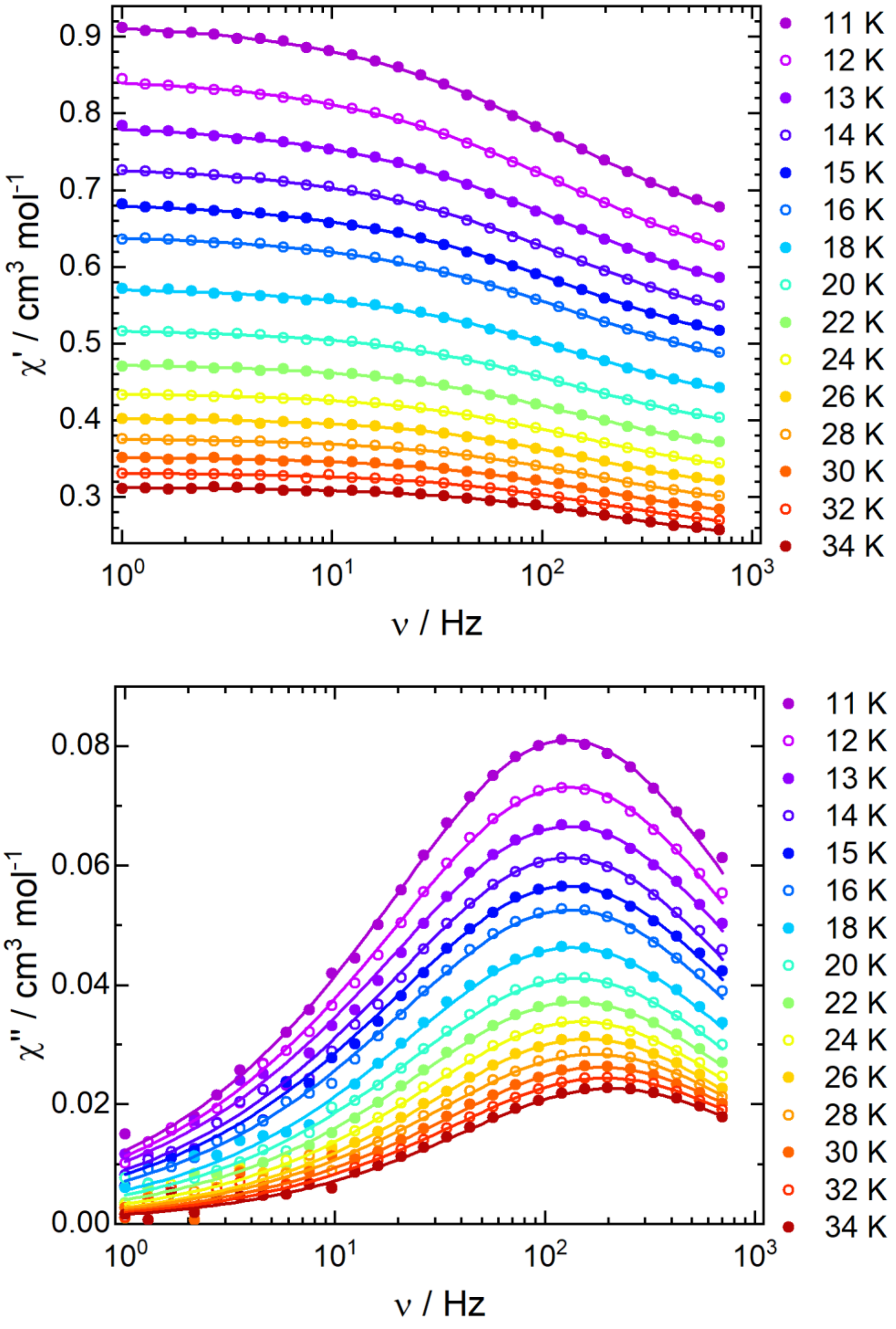

Figure S41. AC frequency dependences of the real (χ', top) and imaginary (χ'', bottom) parts of the AC susceptibility for **1**, between 11 and 34 K for ac frequencies between 1 and 700 Hz (in a zero-dc field). Solid lines are the generalised Debye fit of the ac data used to extract the temperature dependence of the relaxation time.

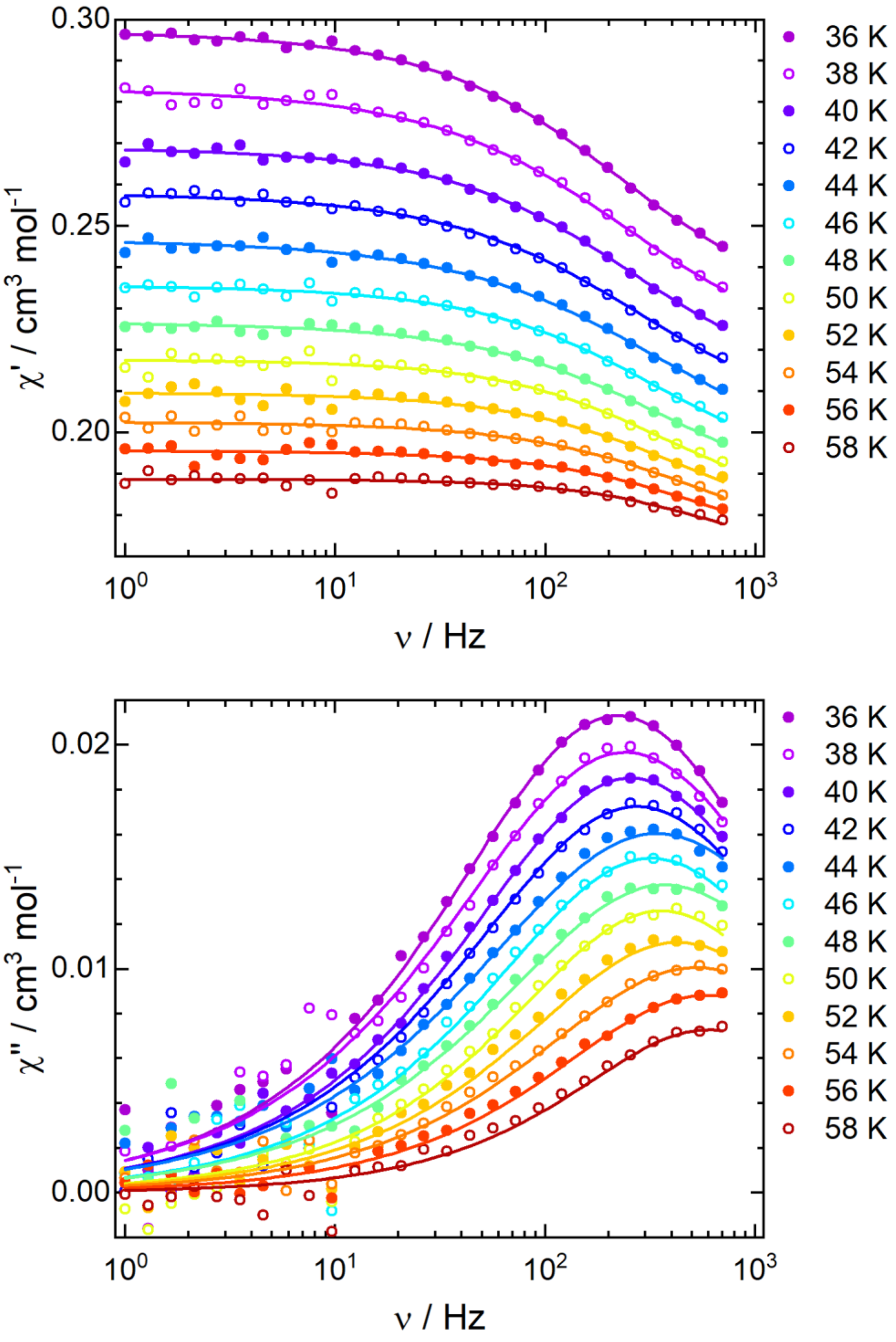

Figure S42. AC frequency dependences of the real (χ', top) and imaginary (χ'', bottom) parts of the AC susceptibility for **1**, between 36 and 58 K for ac frequencies between 1 and 700 Hz (in a zero-dc field). Solid lines are the generalised Debye fit of the ac data used to extract the temperature dependence of the relaxation time.

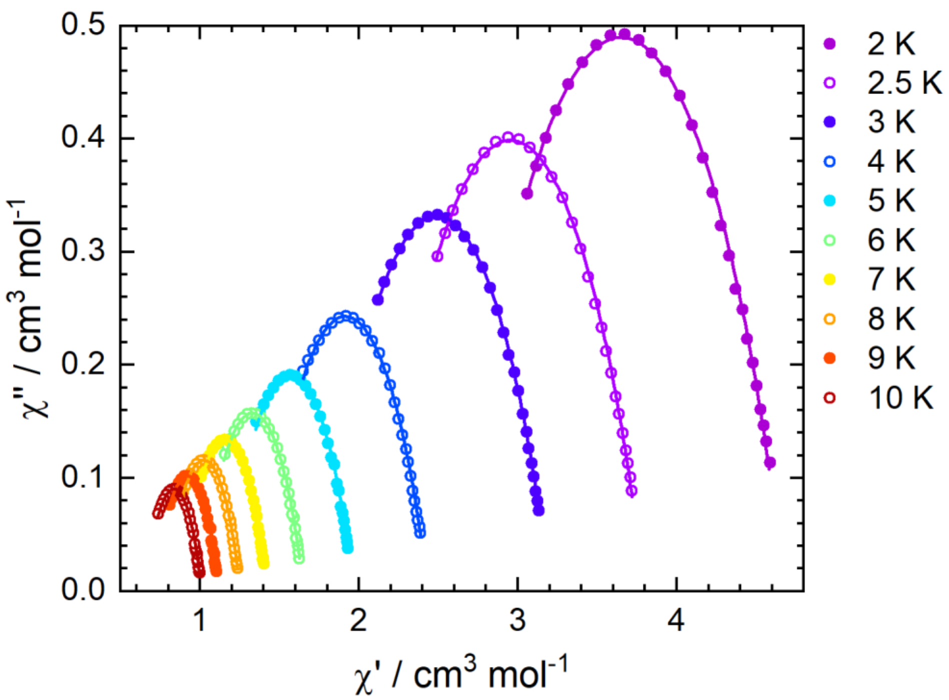

Figure S43. Cole-Cole plots measured in the temperature range of 2-10 K for **1**.

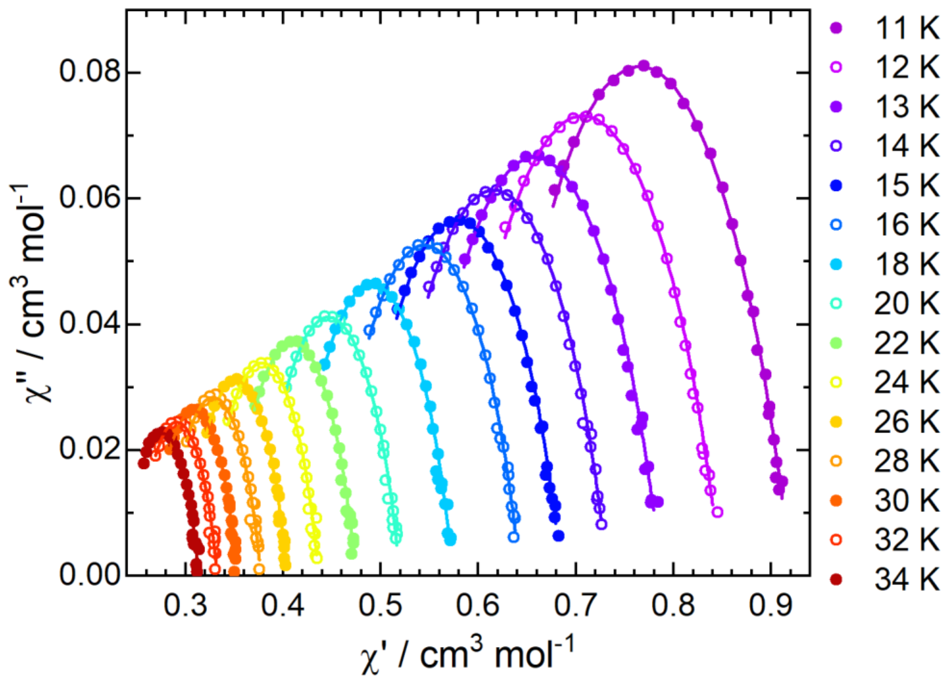

Figure S44. Cole-Cole plots measured in the temperature range of 11-34 K for **1**.

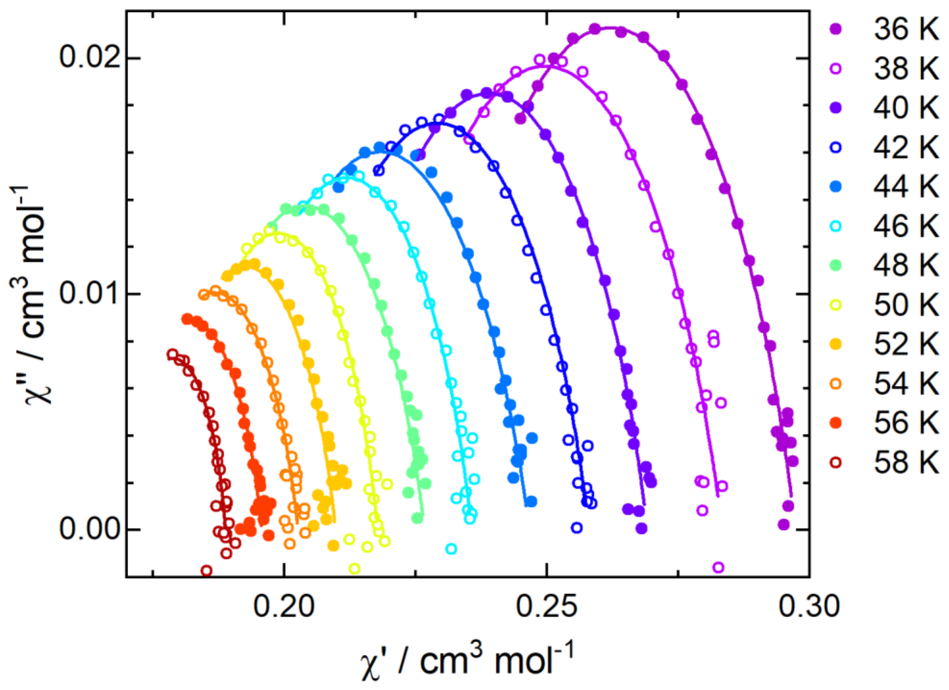

Figure S45. Cole-Cole plots measured in the temperature range of 36-58 K for **1**.

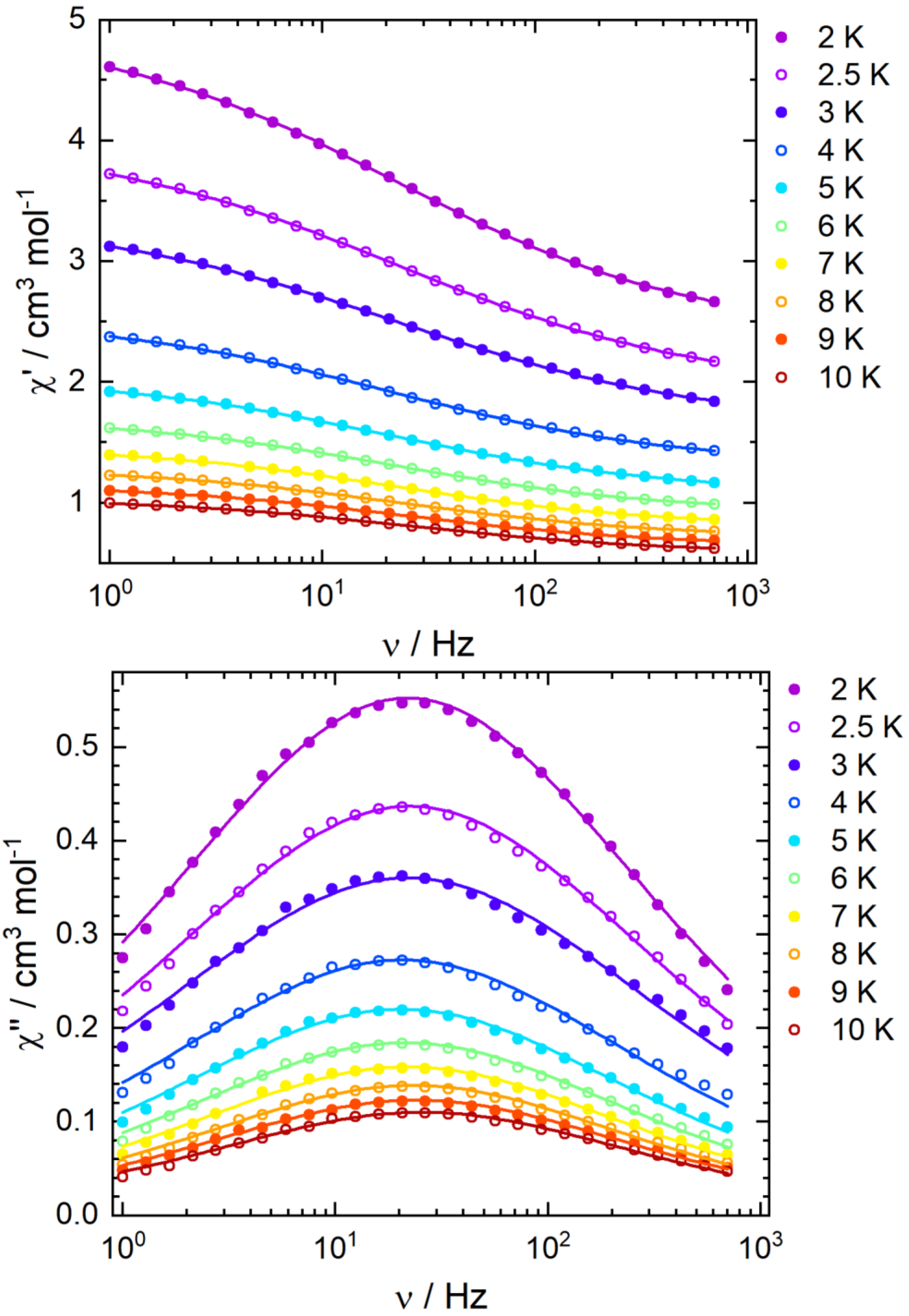

Figure S46. AC frequency dependences of the real (χ', top) and imaginary (χ'', bottom) parts of the ac susceptibility for **2**, between 2 and 10 K for ac frequencies between 1 and 700 Hz (in a zero-dc field). Solid lines are the generalised Debye fit of the ac data used to extract the temperature dependence of the relaxation time.

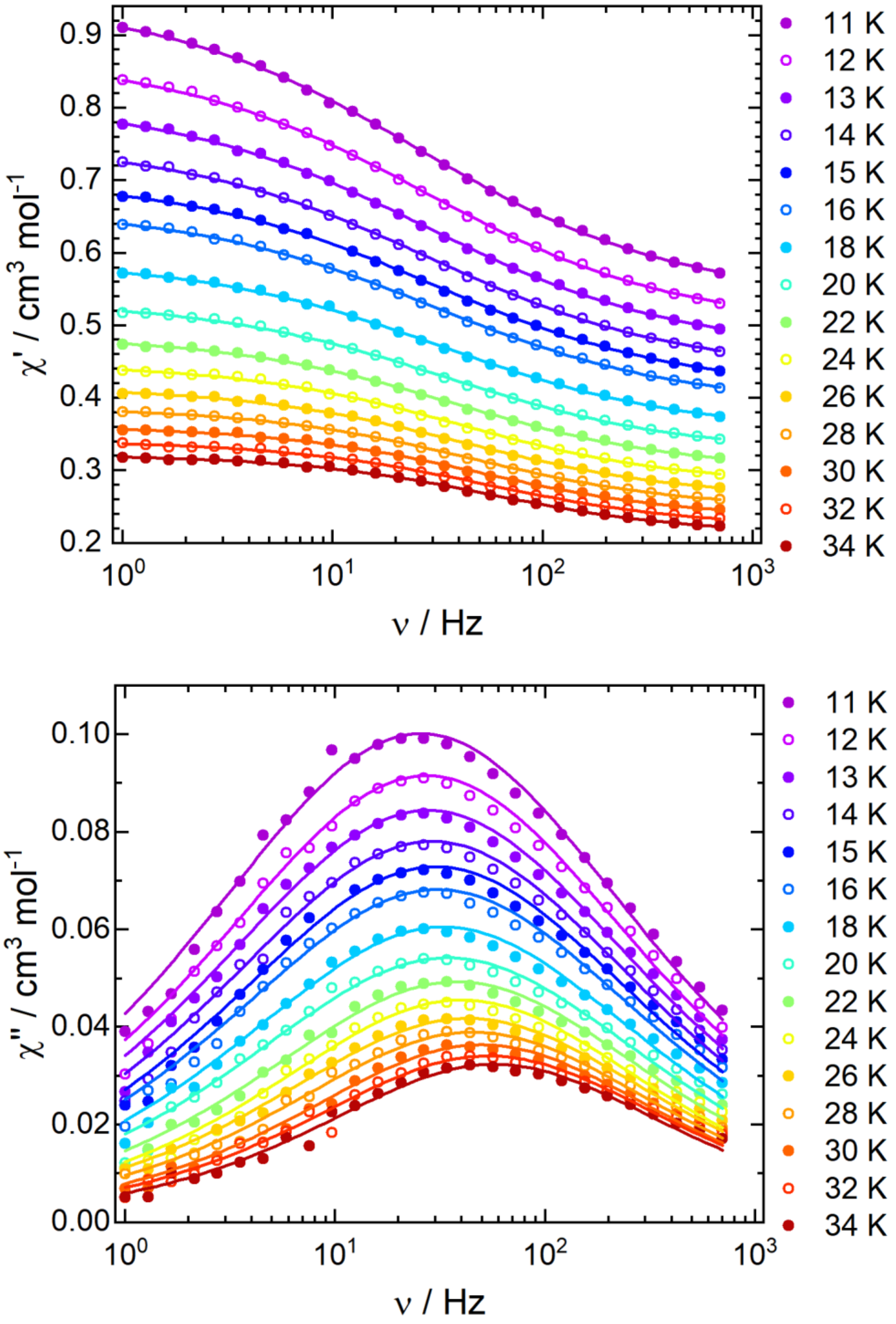

Figure S47. AC frequency dependences of the real (χ', top) and imaginary (χ'', bottom) parts of the ac susceptibility for **2**, between 11 and 34 K for ac frequencies between 1 and 700 Hz (in a zero-dc field). Solid lines are the generalised Debye fit of the ac data used to extract the temperature dependence of the relaxation time.

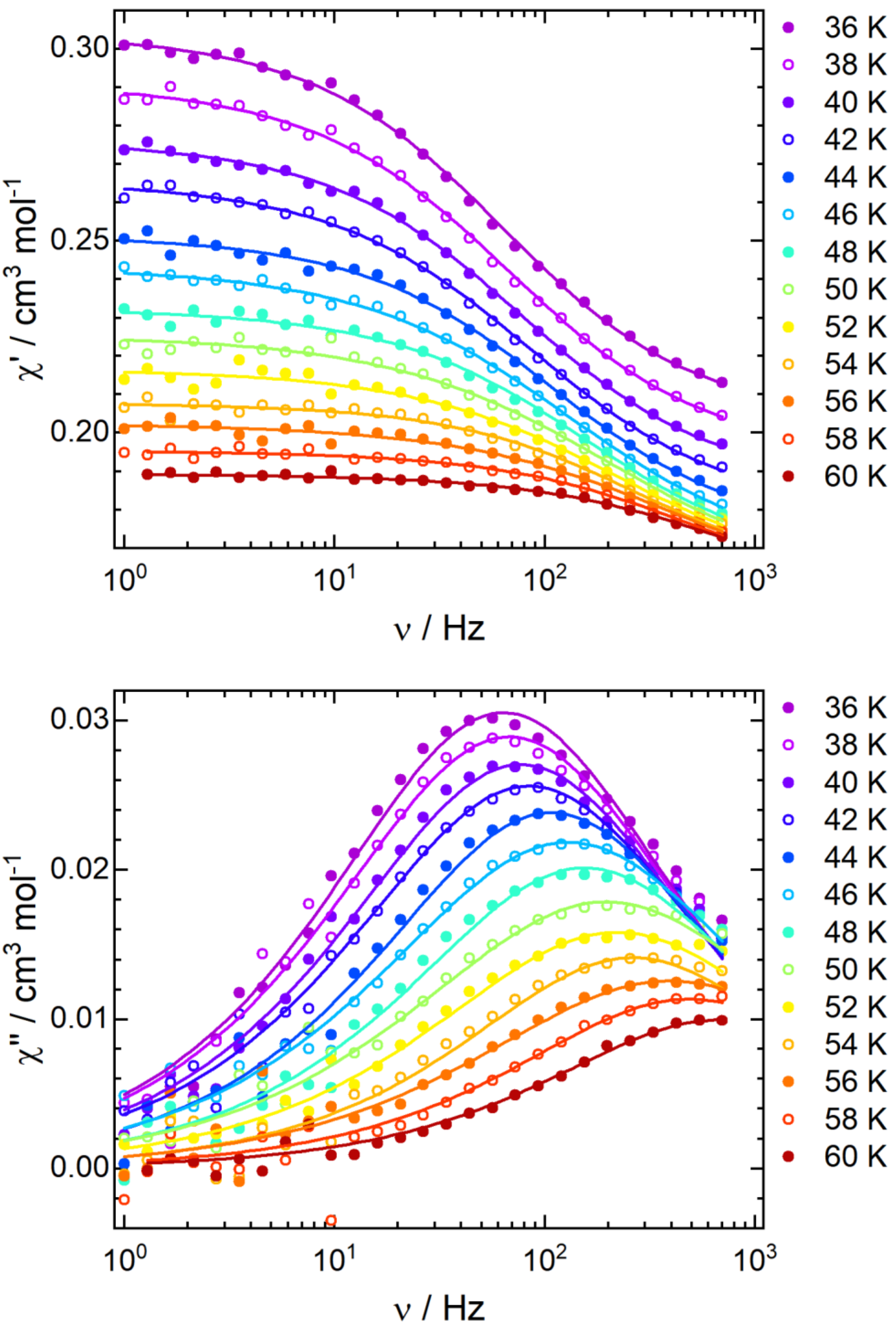

Figure S48. AC frequency dependences of the real (χ', top) and imaginary (χ'', bottom) parts of the ac susceptibility for **2**, between 36 and 60 K for ac frequencies between 1 and 700 Hz (in a zero-dc field). Solid lines are the generalised Debye fit of the ac data used to extract the temperature dependence of the relaxation time.

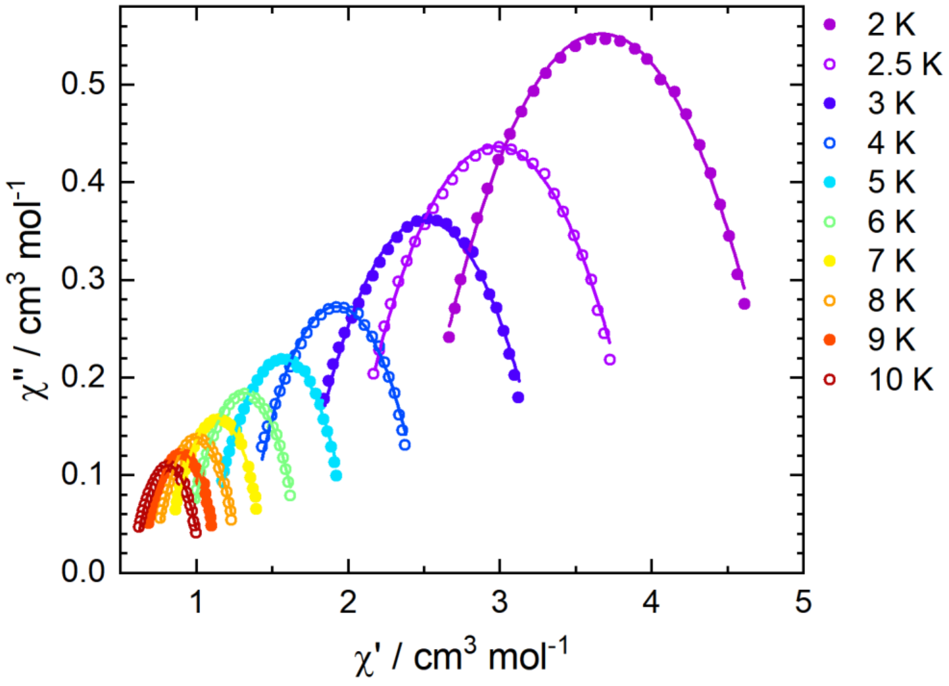

Figure S49. Cole-Cole plots measured in the temperature range of 2-10 K for **2**.

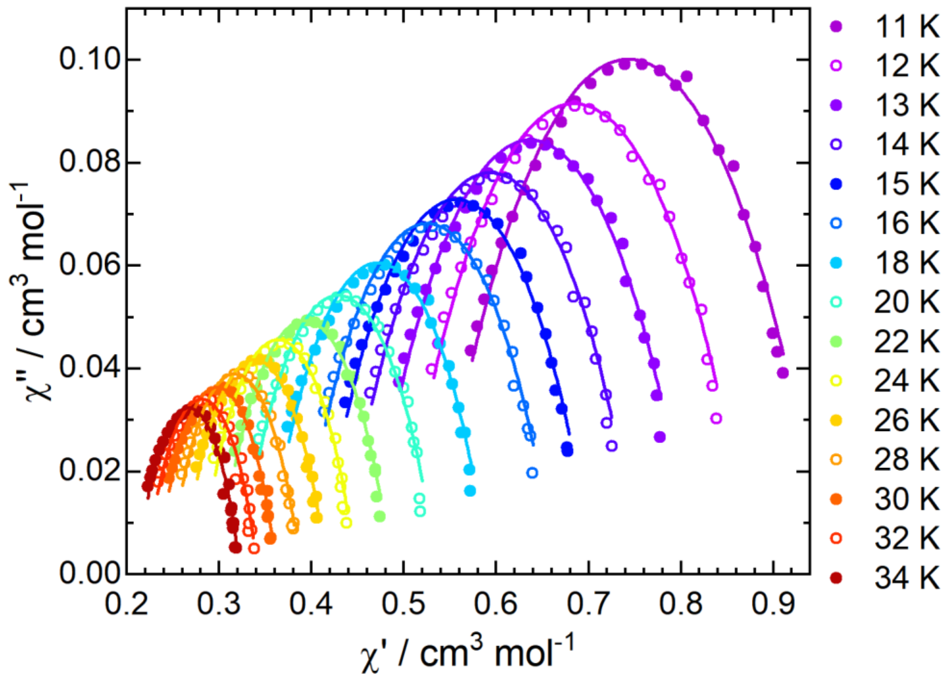

Figure S50. Cole-Cole plots measured in the temperature range of 11-34 K for **2**.

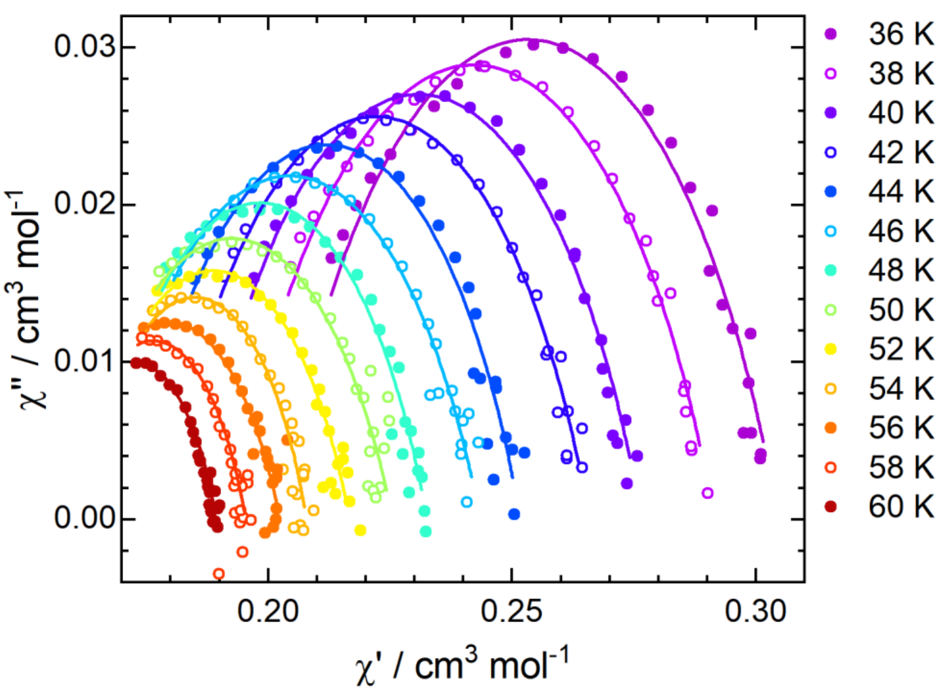

Figure S51. Cole-Cole plots measured in the temperature range of 36-60 K for **2**.

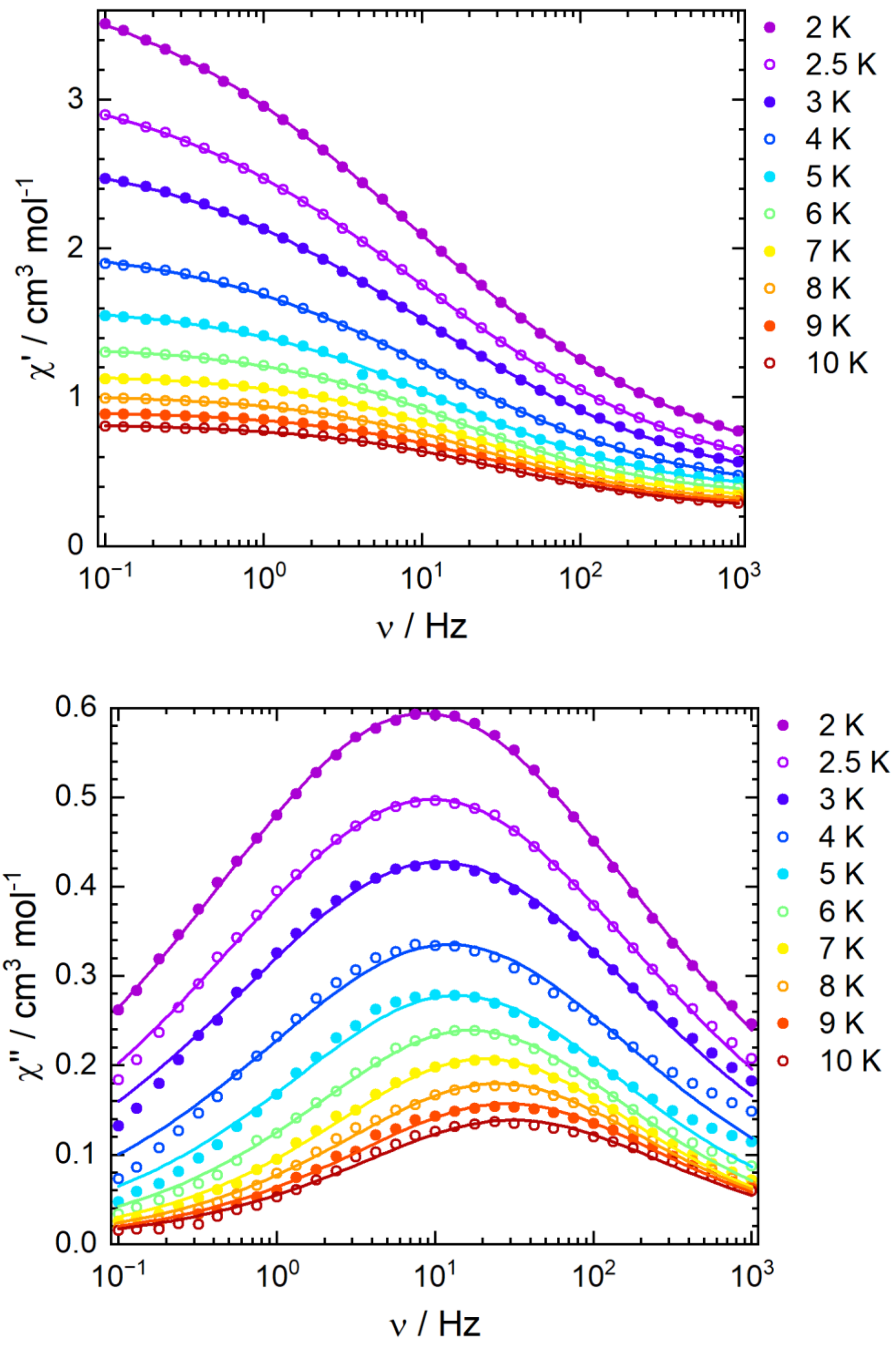

Figure S52. AC frequency dependences of the real (χ', top) and imaginary (χ'', bottom) parts of the AC susceptibility for the 200 mM frozen solution of **1** in 9:1 toluene:hexane, between 2 and 10 K for ac frequencies between 0.1 and 1000 Hz (in a zero-dc field). Solid lines are the generalised Debye fit of the ac data used to extract the temperature dependence of the relaxation time.

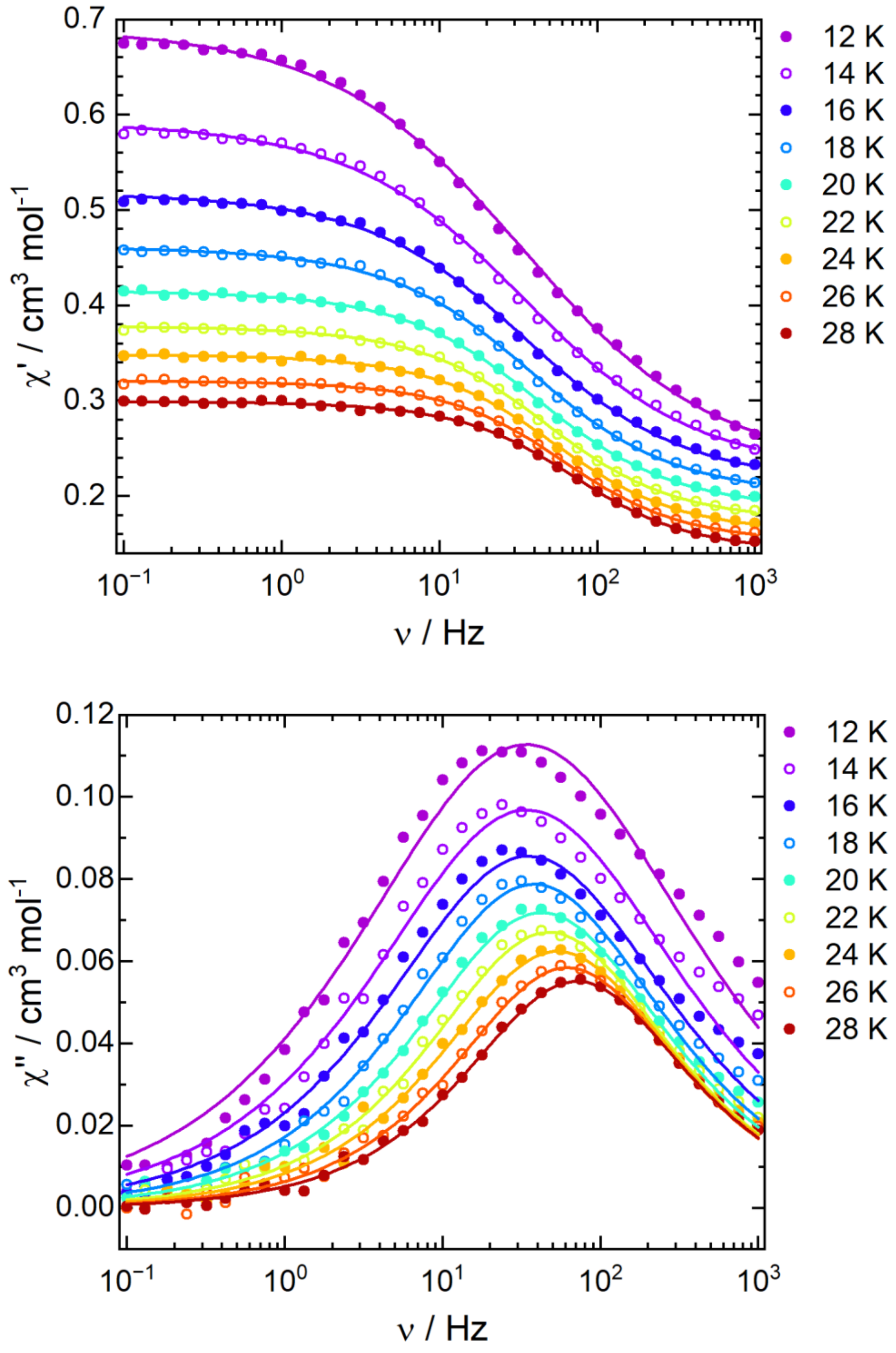

Figure S53. AC frequency dependences of the real (χ', top) and imaginary (χ'', bottom) parts of the AC susceptibility for the 200 mM frozen solution of **1** in 9:1 toluene:hexane, between 12 and 28 K for ac frequencies between 0.1 and 1000 Hz (in a zero-dc field). Solid lines are the generalised Debye fit of the ac data used to extract the temperature dependence of the relaxation time.

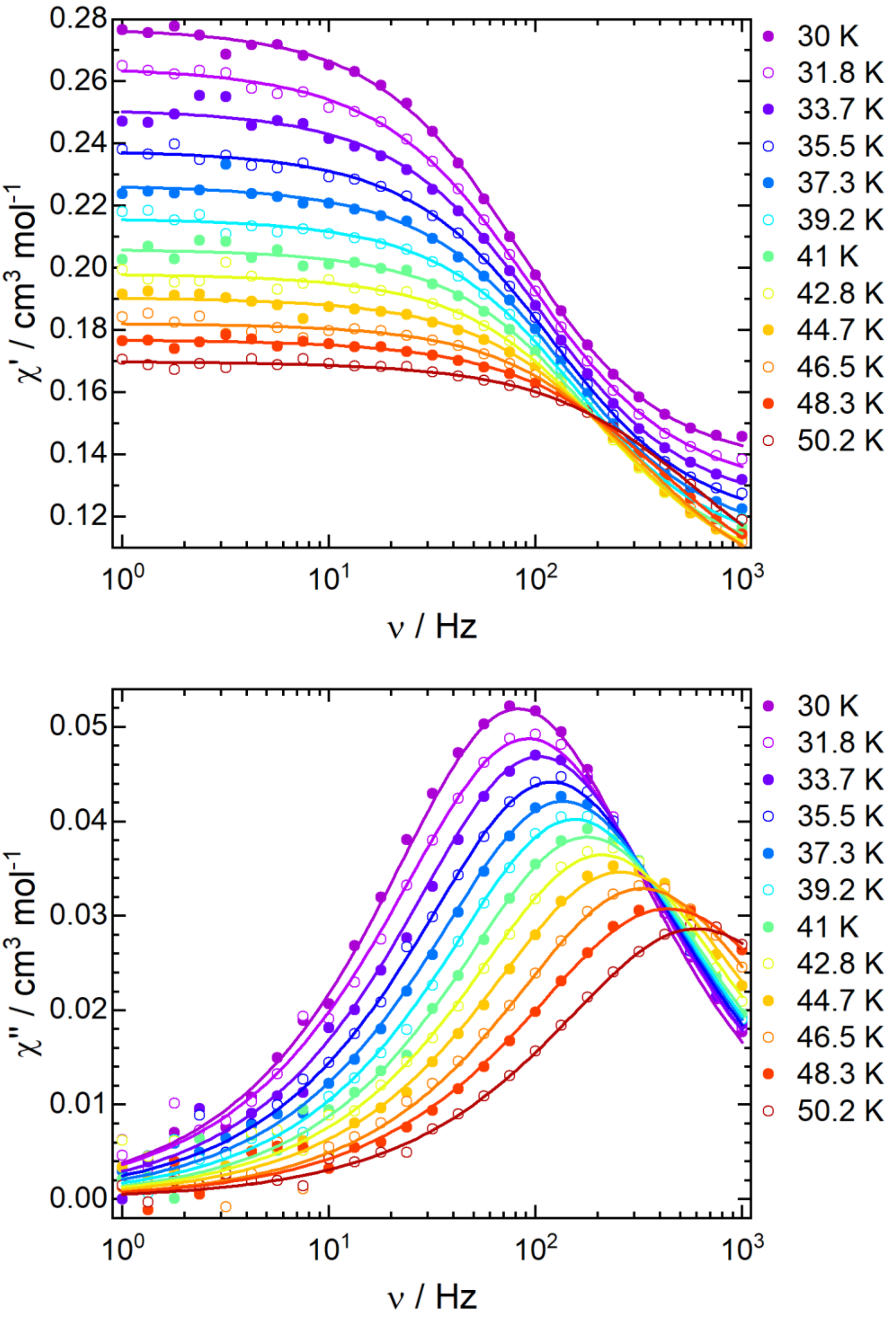

Figure S54. AC frequency dependences of the real (χ', top) and imaginary (χ'', bottom) parts of the AC susceptibility for the 200 mM frozen solution of **1** in 9:1 toluene:hexane, between 30 and 50 K for ac frequencies between 1 and 1000 Hz (in a zero-dc field). Solid lines are the generalised Debye fit of the ac data used to extract the temperature dependence of the relaxation time.

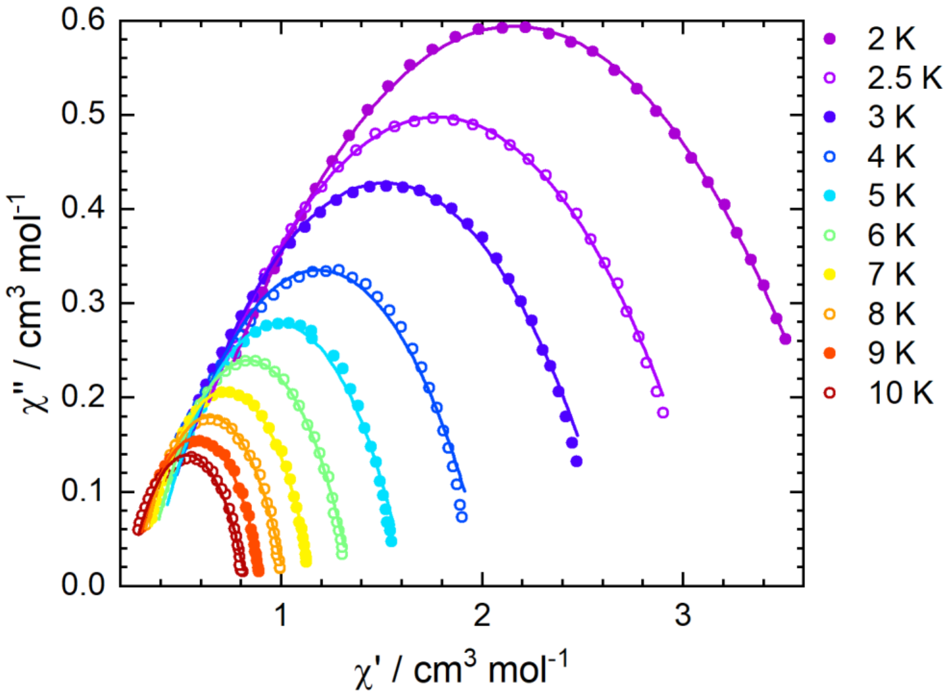

Figure S55. Cole-Cole plots measured in the temperature range of 2-10 K for the 200 mM frozen solution of **1** in 9:1 toluene:hexane.

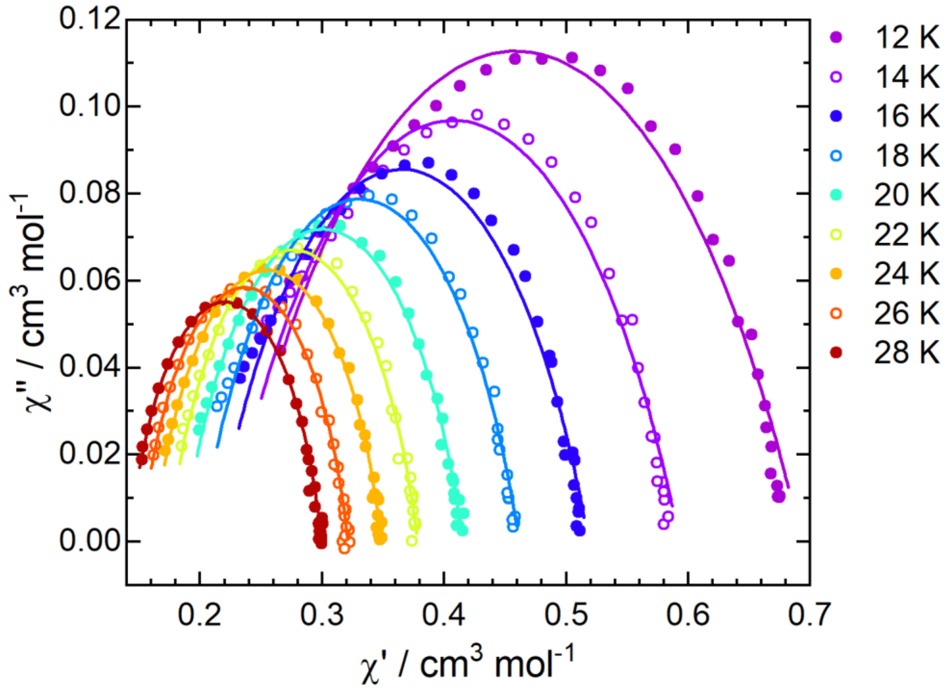

Figure S56. Cole-Cole plots measured in the temperature range of 12-28 K for the 200 mM frozen solution of **1** in 9:1 toluene:hexane.

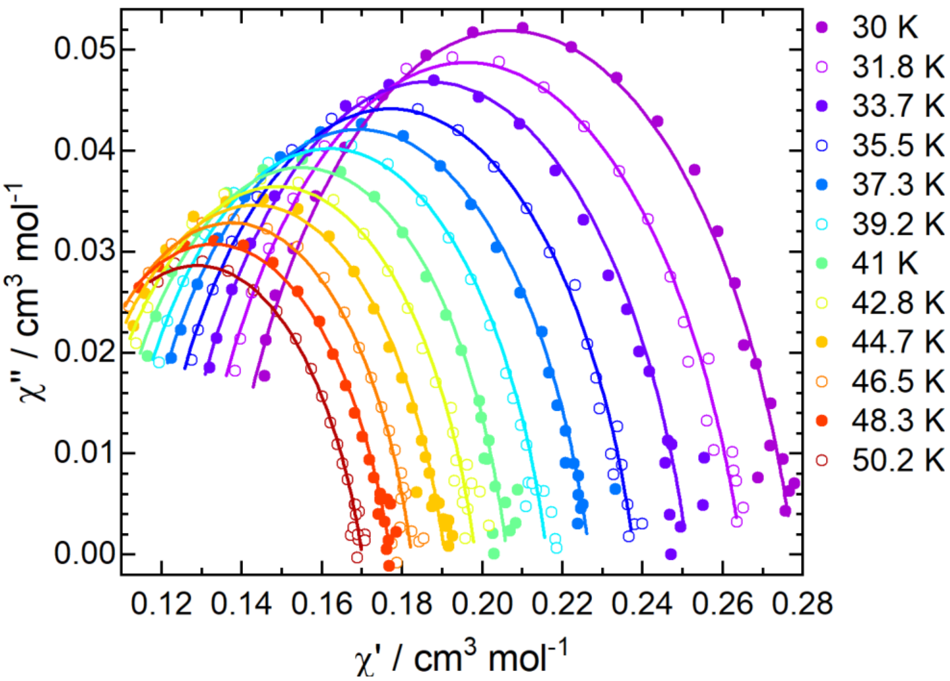

Figure S57. Cole-Cole plots measured in the temperature range of 30-50 K for the 200 mM frozen solution of **1** in 9:1 toluene:hexane.

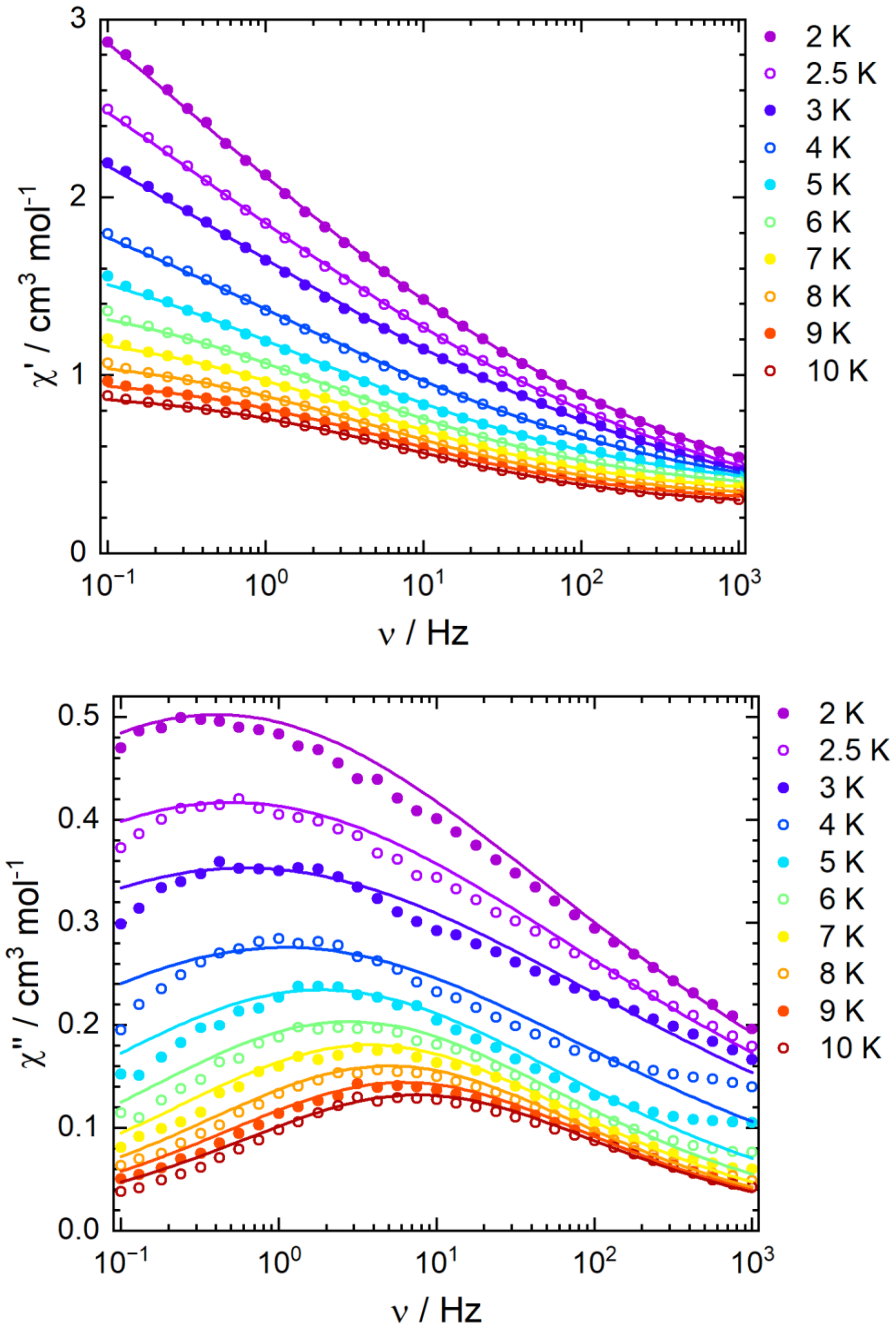

Figure S58. AC frequency dependences of the real (χ', top) and imaginary (χ'', bottom) parts of the AC susceptibility for the 100 mM frozen solution of **1** in 9:1 toluene:hexane, between 2 and 10 K for ac frequencies between 0.1 and 1000 Hz (in a zero-dc field). Solid lines are the generalised Debye fit of the ac data used to extract the temperature dependence of the relaxation time.

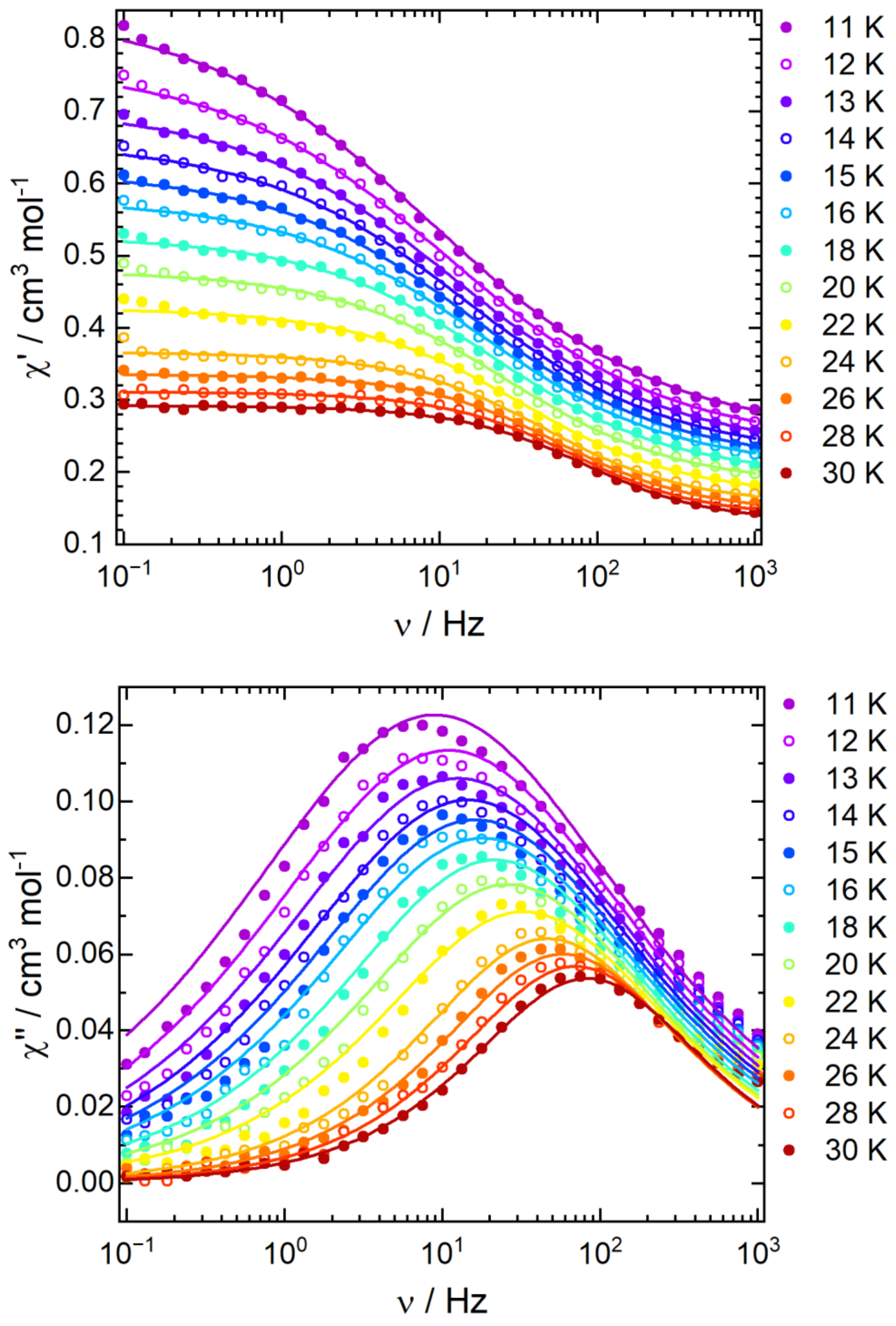

Figure S59. AC frequency dependences of the real (χ', top) and imaginary (χ'', bottom) parts of the AC susceptibility for the 100 mM frozen solution of **1** in 9:1 toluene:hexane, between 11 and 30 K for ac frequencies between 0.1 and 1000 Hz (in a zero-dc field). Solid lines are the generalised Debye fit of the ac data used to extract the temperature dependence of the relaxation time.

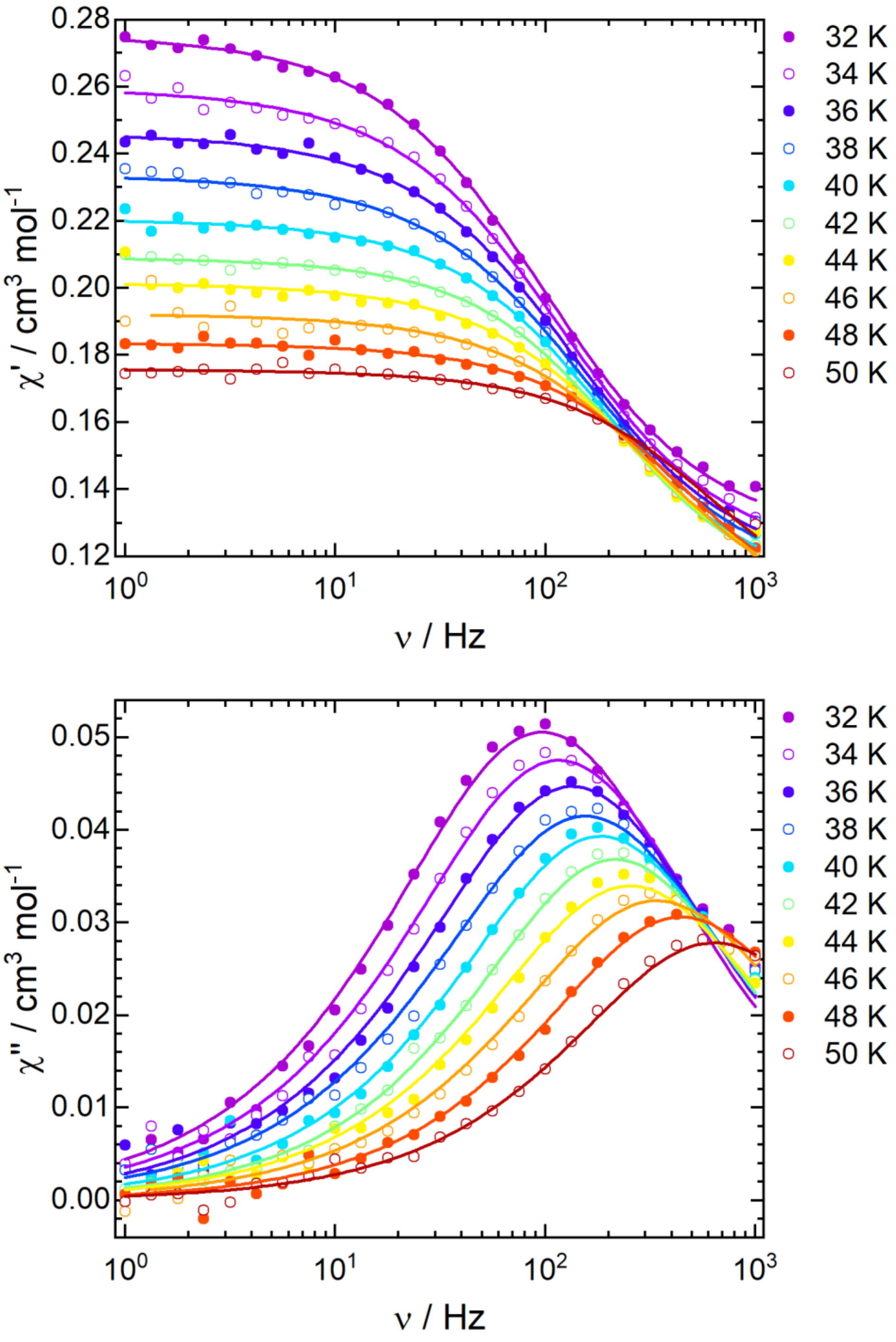

Figure S60. AC frequency dependences of the real (χ', top) and imaginary (χ'', bottom) parts of the AC susceptibility for the 100 mM frozen solution of **1** in 9:1 toluene:hexane, between 32 and 50 K for ac frequencies between 1 and 1000 Hz (in a zero-dc field). Solid lines are the generalised Debye fit of the ac data used to extract the temperature dependence of the relaxation time.

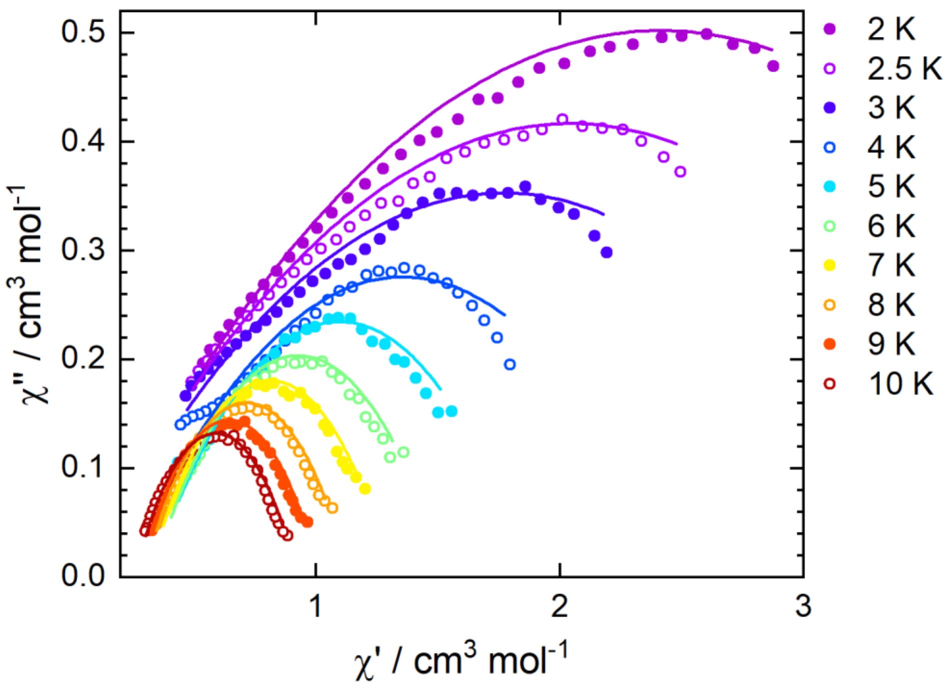

Figure S61. Cole-Cole plots measured in the temperature range of 2-10 K for the 100 mM frozen solution of **1** in 9:1 toluene:hexane.

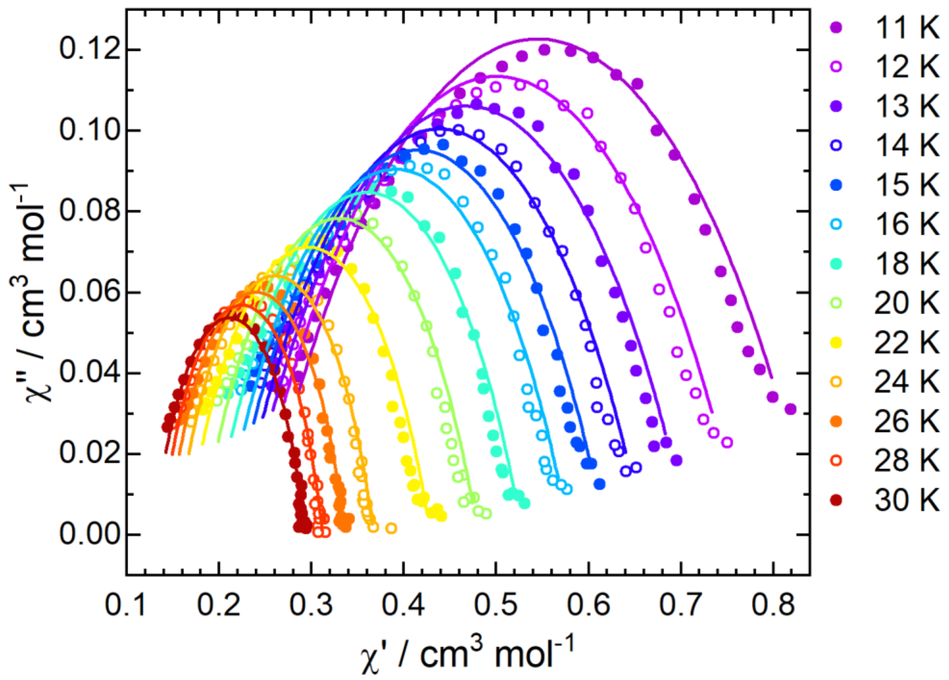

Figure S62. Cole-Cole plots measured in the temperature range of 11-30 K for the 100 mM frozen solution of **1** in 9:1 toluene:hexane.

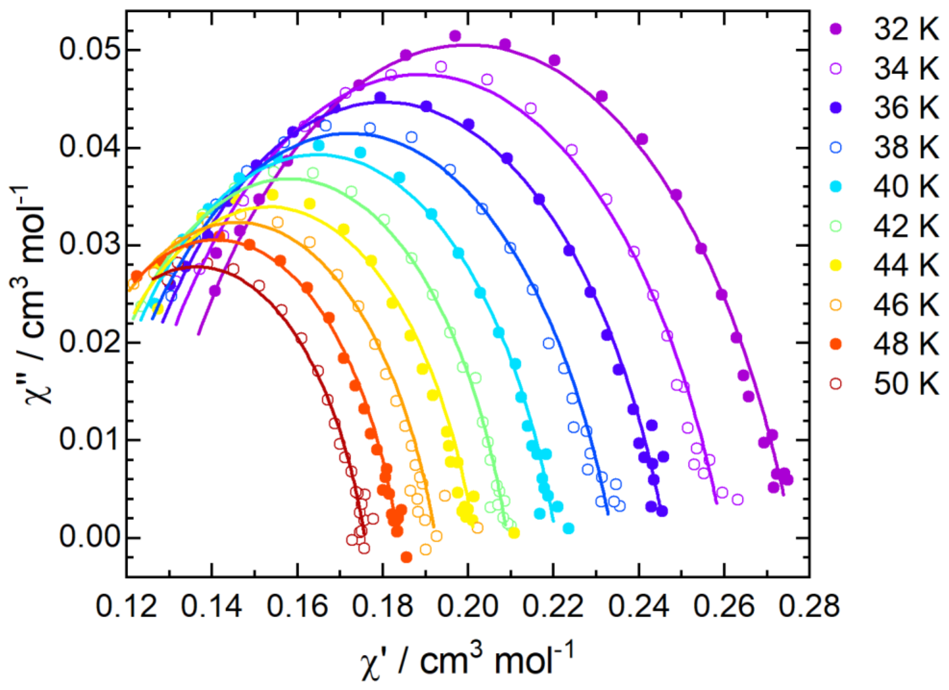

Figure S63. Cole-Cole plots measured in the temperature range of 32-50 K for the 100 mM frozen solution of **1** in 9:1 toluene:hexane.

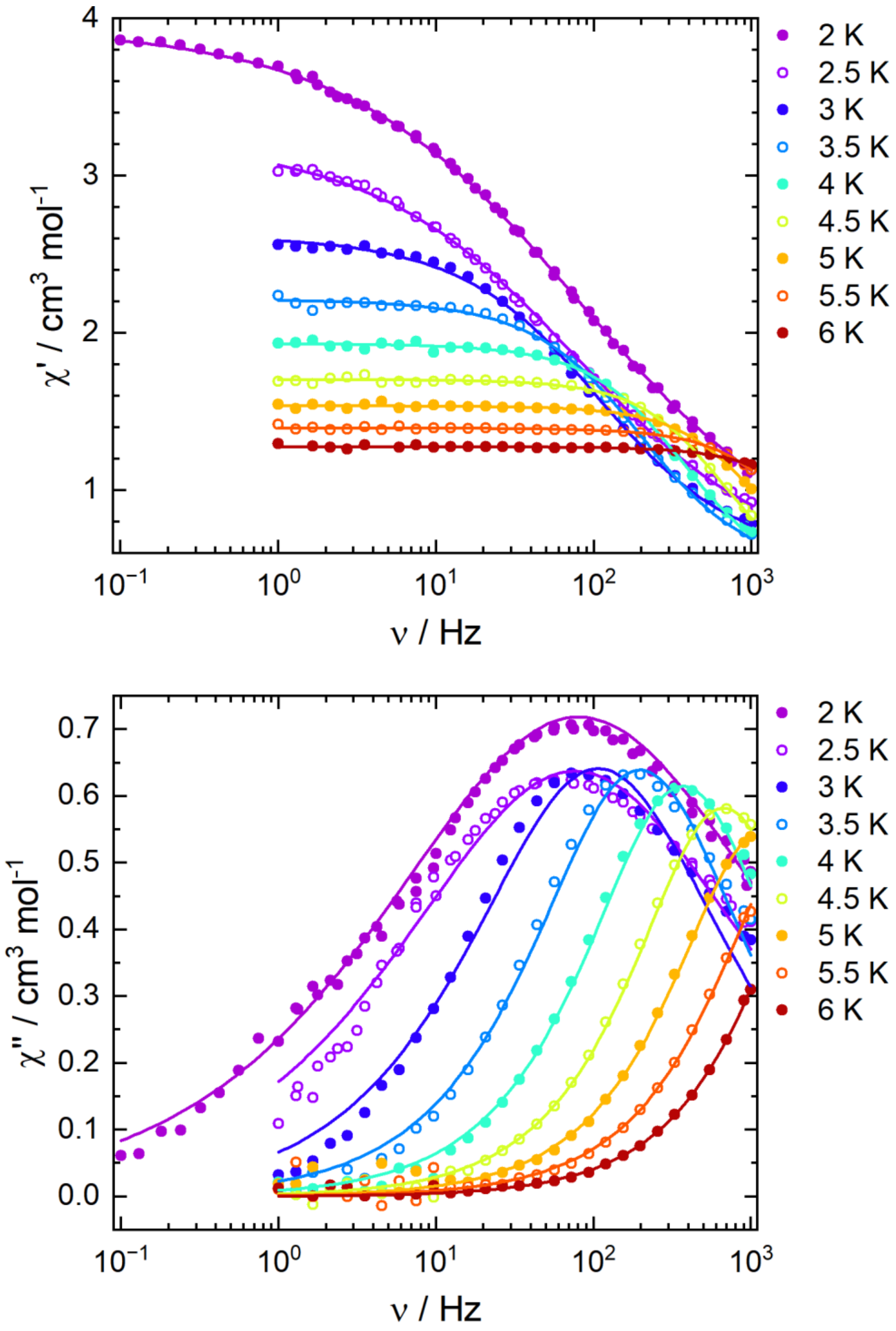

Figure S64. AC frequency dependences of the real (χ', top) and imaginary (χ'', bottom) parts of the AC susceptibility for **5%Dy@1-Y**, between 2 and 6 K for ac frequencies between 1 and 1000 Hz, or 0.1-1000 Hz at 2 K (in a zero-dc field). Solid lines are the generalised Debye fit of the ac data used to extract the temperature dependence of the relaxation time.

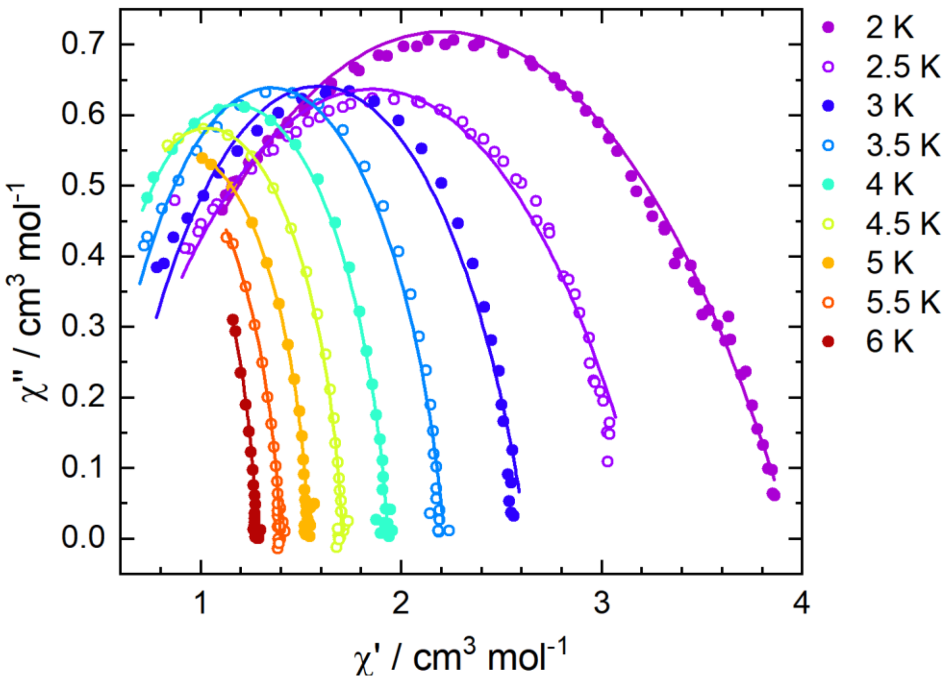

Figure S65. Cole-Cole plots measured in the temperature range of 2-6 K for **5%Dy@1-Y**.

Table S8. The fitting parameters of ac data for **1** under zero dc field to the Generalised Debye Model using the CC-FIT2 program.

T	τ	τ^{err}	χ_S	χ_S^{err}	χ_T	χ_T^{err}	α	α^{err}
(K)	(s)		($cm^3 mol^{-1}$)		($cm^3 mol^{-1}$)			
2.00	1.60E-3	1.62E-5	2.621	7.25E-3	4.680	3.02E-3	0.434	2.55E-3
2.50	1.42E-3	1.33E-5	2.112	5.47E-3	3.796	2.10E-3	0.437	2.24E-3
3.00	1.30E-3	1.12E-5	1.777	4.20E-3	3.200	1.52E-3	0.444	1.96E-3
4.00	1.28E-3	1.79E-5	1.396	5.01E-3	2.433	1.79E-3	0.442	3.19E-3
5.00	1.32E-3	1.92E-5	1.167	4.06E-3	1.966	1.48E-3	0.432	3.45E-3
6.00	1.34E-3	1.87E-5	1.008	3.19E-3	1.651	1.18E-3	0.419	3.46E-3
7.00	1.34E-3	1.73E-5	0.890	2.50E-3	1.422	9.19E-4	0.406	3.33E-3
8.00	1.31E-3	1.61E-5	0.794	2.06E-3	1.251	7.45E-4	0.402	3.18E-3
9.00	1.29E-3	1.98E-5	0.721	2.25E-3	1.117	8.08E-4	0.394	4.05E-3
10.00	1.24E-3	2.08E-5	0.660	2.17E-3	1.008	7.60E-4	0.390	4.41E-3
11.00	1.24E-3	2.01E-5	0.610	1.87E-3	0.920	6.55E-4	0.387	4.28E-3
12.00	1.23E-3	2.20E-5	0.565	1.88E-3	0.849	6.52E-4	0.393	4.65E-3
13.00	1.22E-3	2.93E-5	0.528	2.29E-3	0.788	7.93E-4	0.397	6.15E-3
14.00	1.24E-3	2.25E-5	0.499	1.59E-3	0.732	5.57E-4	0.383	4.87E-3
15.00	1.23E-3	2.46E-5	0.471	1.61E-3	0.686	5.63E-4	0.383	5.37E-3
16.00	1.21E-3	1.87E-5	0.447	1.15E-3	0.643	3.98E-4	0.372	4.24E-3
18.00	1.19E-3	3.06E-5	0.408	1.69E-3	0.574	5.77E-4	0.353	7.46E-3
20.00	1.16E-3	2.56E-5	0.373	1.28E-3	0.520	4.31E-4	0.350	6.39E-3
22.00	1.13E-3	2.61E-5	0.345	1.21E-3	0.474	4.02E-4	0.338	6.88E-3
24.00	1.07E-3	2.45E-5	0.320	1.09E-3	0.436	3.49E-4	0.326	6.90E-3
26.00	1.01E-3	2.58E-5	0.300	1.12E-3	0.404	3.43E-4	0.318	7.75E-3
28.00	9.29E-4	3.49E-5	0.279	1.52E-3	0.377	4.35E-4	0.328	1.08E-2
30.00	8.69E-4	3.43E-5	0.264	1.47E-3	0.353	4.03E-4	0.323	1.12E-2
32.00	8.23E-4	3.74E-5	0.250	1.58E-3	0.332	4.16E-4	0.318	1.28E-2
34.00	7.76E-4	3.24E-5	0.238	1.35E-3	0.313	3.41E-4	0.307	1.19E-2
36.00	7.19E-4	3.26E-5	0.227	1.38E-3	0.297	3.28E-4	0.308	1.26E-2
38.00	6.61E-4	4.90E-5	0.216	2.11E-3	0.283	4.85E-4	0.328	1.91E-2
40.00	6.24E-4	3.12E-5	0.209	1.33E-3	0.269	2.83E-4	0.299	1.35E-2
42.00	5.81E-4	3.36E-5	0.200	1.44E-3	0.258	2.86E-4	0.314	1.45E-2
44.00	4.68E-4	4.46E-5	0.190	2.21E-3	0.247	3.64E-4	0.344	1.99E-2
46.00	4.97E-4	4.56E-5	0.188	1.99E-3	0.236	3.49E-4	0.286	2.34E-2
48.00	4.29E-4	5.12E-5	0.181	2.38E-3	0.227	3.64E-4	0.308	2.68E-2
50.00	4.45E-4	5.02E-5	0.180	2.08E-3	0.218	3.48E-4	0.251	3.07E-2
52.00	3.73E-4	5.89E-5	0.175	2.59E-3	0.210	3.50E-4	0.268	3.74E-2
54.00	3.04E-4	5.19E-5	0.171	2.53E-3	0.203	2.79E-4	0.278	3.56E-2
56.00	2.73E-4	5.92E-5	0.169	2.83E-3	0.196	2.81E-4	0.251	4.61E-2
58.00	2.63E-4	5.90E-5	0.169	2.45E-3	0.189	2.35E-4	0.192	5.45E-2

Table S9. The fitting parameters of ac data for **2** under zero dc field to the Generalised Debye Model using the CC-FIT2 program.

T	τ	τ^{err}	χ_S	χ_S^{err}	χ_T	χ_T^{err}	α	α^{err}
(K)	(s)		(cm^3 mol^{-1})		(cm^3 mol^{-1})			
2.00	7.27E-3	7.61E-5	2.360	7.16E-3	4.972	8.10E-3	0.490	2.97E-3
2.50	7.12E-3	8.92E-5	1.911	6.97E-3	4.029	7.77E-3	0.502	3.47E-3
3.00	7.25E-3	1.13E-4	1.627	7.11E-3	3.383	8.03E-3	0.504	4.27E-3
4.00	7.71E-3	1.20E-4	1.299	5.01E-3	2.548	5.88E-3	0.475	4.55E-3
5.00	7.77E-3	1.06E-4	1.074	3.44E-3	2.049	4.06E-3	0.460	4.13E-3
6.00	7.50E-3	8.44E-5	0.915	2.37E-3	1.714	2.74E-3	0.449	3.50E-3
7.00	7.26E-3	9.30E-5	0.797	2.30E-3	1.475	2.65E-3	0.443	4.03E-3
8.00	6.87E-3	9.22E-5	0.709	2.16E-3	1.293	2.35E-3	0.435	4.33E-3
9.00	6.59E-3	8.64E-5	0.637	1.90E-3	1.156	2.02E-3	0.436	4.22E-3
10.00	6.24E-3	8.75E-5	0.579	1.84E-3	1.042	1.89E-3	0.433	4.54E-3
11.00	6.22E-3	1.07E-4	0.532	2.07E-3	0.955	2.12E-3	0.437	5.55E-3
12.00	5.86E-3	1.08E-4	0.494	2.05E-3	0.875	2.02E-3	0.430	6.03E-3
13.00	5.75E-3	1.09E-4	0.460	1.95E-3	0.812	1.90E-3	0.430	6.18E-3
14.00	5.51E-3	1.05E-4	0.432	1.82E-3	0.755	1.72E-3	0.425	6.27E-3
15.00	5.28E-3	1.08E-4	0.408	1.81E-3	0.705	1.67E-3	0.418	6.80E-3
16.00	5.18E-3	1.07E-4	0.387	1.74E-3	0.664	1.58E-3	0.417	6.91E-3
18.00	4.86E-3	1.05E-4	0.351	1.61E-3	0.592	1.41E-3	0.407	7.32E-3
20.00	4.64E-3	1.14E-4	0.321	1.66E-3	0.536	1.40E-3	0.404	8.35E-3
22.00	4.21E-3	8.78E-5	0.298	1.28E-3	0.487	1.02E-3	0.387	7.32E-3
24.00	3.98E-3	1.05E-4	0.279	1.48E-3	0.448	1.14E-3	0.372	9.45E-3
26.00	3.73E-3	8.53E-5	0.260	1.21E-3	0.417	8.91E-4	0.377	8.08E-3
28.00	3.50E-3	7.62E-5	0.245	1.07E-3	0.388	7.59E-4	0.365	7.83E-3
30.00	3.20E-3	7.55E-5	0.233	1.08E-3	0.362	7.26E-4	0.349	8.68E-3
32.00	2.97E-3	8.24E-5	0.220	1.21E-3	0.342	7.72E-4	0.348	1.01E-2
34.00	2.78E-3	7.44E-5	0.211	1.10E-3	0.322	6.77E-4	0.330	1.01E-2
36.00	2.50E-3	6.20E-5	0.202	9.65E-4	0.304	5.56E-4	0.316	9.43E-3
38.00	2.36E-3	7.55E-5	0.193	1.20E-3	0.291	6.65E-4	0.325	1.18E-2
40.00	2.07E-3	4.91E-5	0.185	8.44E-4	0.276	4.29E-4	0.319	8.66E-3
42.00	1.89E-3	5.29E-5	0.178	9.63E-4	0.266	4.59E-4	0.325	9.90E-3
44.00	1.49E-3	5.79E-5	0.172	1.27E-3	0.252	5.16E-4	0.310	1.35E-2
46.00	1.23E-3	5.03E-5	0.165	1.26E-3	0.243	4.42E-4	0.351	1.20E-2
48.00	1.03E-3	5.98E-5	0.164	1.66E-3	0.233	5.14E-4	0.322	1.76E-2
50.00	8.08E-4	7.35E-5	0.160	2.33E-3	0.226	5.97E-4	0.367	2.21E-2
52.00	7.23E-4	7.90E-5	0.161	2.48E-3	0.217	5.85E-4	0.343	2.74E-2
54.00	6.06E-4	6.12E-5	0.162	2.05E-3	0.208	4.25E-4	0.296	2.71E-2
56.00	4.04E-4	8.12E-5	0.157	3.66E-3	0.202	5.31E-4	0.359	3.78E-2
58.00	3.26E-4	4.21E-5	0.157	2.15E-3	0.195	2.71E-4	0.313	2.57E-2
60.00	2.30E-4	3.54E-5	0.156	2.27E-3	0.189	1.98E-4	0.307	2.62E-2

Table S10. The fitting parameters of ac data for a 200 mM solution of **1** in 9:1 toluene/hexane under zero dc field to the Generalised Debye Model using the CC-FIT2 program.

T	τ	τ^{err}	χ_S	χ_S^{err}	χ_T	χ_T^{err}	α	α^{err}
**(K)**	**(s)**		**(cm^3 mol^{-1})**		**(cm^3 mol^{-1})**			
2.00	1.87E-2	1.21E-4	0.395	4.58E-3	3.920	4.97E-3	0.586	1.24E-3
2.50	1.67E-2	1.35E-4	0.351	4.79E-3	3.199	4.91E-3	0.572	1.62E-3
3.00	1.52E-2	2.15E-4	0.327	7.18E-3	2.699	7.01E-3	0.559	2.94E-3
4.00	1.29E-2	3.13E-4	0.331	9.23E-3	2.034	8.31E-3	0.522	5.53E-3
5.00	1.16E-2	3.29E-4	0.339	8.51E-3	1.625	7.28E-3	0.480	7.20E-3
6.00	9.50E-3	1.35E-4	0.320	3.63E-3	1.350	2.83E-3	0.446	3.90E-3
7.00	7.78E-3	8.87E-5	0.293	2.56E-3	1.158	1.81E-3	0.430	3.21E-3
8.00	6.49E-3	8.87E-5	0.265	2.75E-3	1.019	1.78E-3	0.433	3.76E-3
9.00	5.58E-3	9.39E-5	0.245	3.04E-3	0.911	1.82E-3	0.438	4.49E-3
10.00	5.12E-3	1.17E-4	0.233	3.72E-3	0.826	2.13E-3	0.442	5.98E-3
12.00	4.71E-3	1.47E-4	0.224	4.09E-3	0.693	2.26E-3	0.428	8.38E-3
14.00	4.68E-3	1.47E-4	0.222	3.38E-3	0.593	1.90E-3	0.387	9.42E-3
16.00	4.56E-3	1.15E-4	0.212	2.32E-3	0.518	1.31E-3	0.350	8.28E-3
18.00	4.21E-3	8.38E-5	0.200	1.64E-3	0.461	9.16E-4	0.310	7.14E-3
20.00	3.78E-3	5.58E-5	0.186	1.11E-3	0.416	5.98E-4	0.289	5.51E-3
22.00	3.37E-3	4.79E-5	0.173	1.00E-3	0.378	5.20E-4	0.262	5.61E-3
24.00	2.98E-3	4.56E-5	0.161	1.02E-3	0.349	5.05E-4	0.252	6.13E-3
26.00	2.59E-3	3.49E-5	0.151	8.50E-4	0.321	4.01E-4	0.231	5.60E-3
28.00	2.25E-3	2.72E-5	0.142	7.32E-4	0.299	3.26E-4	0.222	5.07E-3
30.00	1.93E-3	2.70E-5	0.135	8.22E-4	0.278	4.90E-4	0.198	6.66E-3
31.83	1.73E-3	2.66E-5	0.127	8.78E-4	0.265	4.87E-4	0.216	7.00E-3
33.67	1.53E-3	3.72E-5	0.122	1.36E-3	0.251	7.07E-4	0.202	1.13E-2
35.50	1.33E-3	2.12E-5	0.116	8.62E-4	0.238	4.13E-4	0.201	7.27E-3
37.33	1.17E-3	2.75E-5	0.111	1.24E-3	0.227	5.49E-4	0.196	1.07E-2
39.17	1.03E-3	2.07E-5	0.107	1.03E-3	0.216	4.26E-4	0.188	9.12E-3
41.00	8.99E-4	2.36E-5	0.104	1.32E-3	0.206	4.97E-4	0.182	1.18E-2
42.83	7.61E-4	2.56E-5	0.099	1.66E-3	0.198	5.52E-4	0.192	1.43E-2
44.67	6.22E-4	1.81E-5	0.096	1.40E-3	0.191	4.00E-4	0.198	1.16E-2
46.50	4.87E-4	1.78E-5	0.093	1.72E-3	0.182	4.24E-4	0.194	1.38E-2
48.33	3.67E-4	1.58E-5	0.089	1.91E-3	0.177	3.48E-4	0.225	1.32E-2
50.17	2.64E-4	1.21E-5	0.088	1.92E-3	0.170	2.57E-4	0.226	1.22E-2

Table S11. The fitting parameters of ac data for a 100 mM solution of **1** in 9:1 toluene/hexane under zero dc field to the Generalised Debye Model using the CC-FIT2 program.

**T**	τ	τ^{err}	χ_S	χ_S^{err}	χ_T	χ_T^{err}	α	α^{err}
**(K)**	**(s)**		**(cm^3 mol^{-1})**		**(cm^3 mol^{-1})**			
2.00	3.96E-1	4.35E-2	0.048	2.15E-2	4.782	8.67E-2	0.734	5.12E-3
2.50	3.19E-1	3.45E-2	0.037	2.01E-2	4.051	7.25E-2	0.739	5.22E-3
3.00	2.62E-1	3.51E-2	0.058	2.35E-2	3.478	7.74E-2	0.741	6.80E-3
4.00	1.45E-1	1.90E-2	0.219	2.11E-2	2.493	5.86E-2	0.697	9.99E-3
5.00	8.53E-2	7.09E-3	0.319	1.28E-2	1.876	2.98E-2	0.627	1.02E-2
6.00	5.69E-2	3.10E-3	0.328	8.34E-3	1.529	1.60E-2	0.584	8.85E-3
7.00	4.22E-2	1.71E-3	0.312	6.07E-3	1.311	9.98E-3	0.557	7.70E-3
8.00	3.18E-2	1.02E-3	0.293	4.62E-3	1.137	6.57E-3	0.537	6.77E-3
9.00	2.56E-2	7.78E-4	0.274	4.17E-3	1.015	5.31E-3	0.527	6.75E-3
10.00	2.10E-2	6.34E-4	0.257	3.98E-3	0.923	4.58E-3	0.518	6.96E-3
11.00	1.78E-2	5.50E-4	0.245	3.87E-3	0.844	4.10E-3	0.505	7.44E-3
12.00	1.46E-2	4.61E-4	0.234	3.73E-3	0.767	3.57E-3	0.487	7.96E-3
13.00	1.27E-2	3.86E-4	0.226	3.37E-3	0.709	3.02E-3	0.473	7.88E-3
14.00	1.10E-2	3.49E-4	0.218	3.35E-3	0.660	2.79E-3	0.456	8.53E-3
15.00	9.85E-3	3.12E-4	0.210	3.18E-3	0.618	2.52E-3	0.444	8.74E-3
16.00	8.82E-3	2.85E-4	0.203	3.08E-3	0.579	2.32E-3	0.429	9.18E-3
18.00	7.37E-3	2.53E-4	0.192	3.06E-3	0.528	2.12E-3	0.405	1.02E-2
20.00	6.14E-3	2.28E-4	0.180	3.10E-3	0.480	1.98E-3	0.388	1.14E-2
22.00	4.88E-3	1.95E-4	0.164	3.09E-3	0.429	1.78E-3	0.371	1.25E-2
24.00	3.42E-3	1.23E-4	0.154	2.52E-3	0.367	1.27E-3	0.310	1.28E-2
26.00	2.74E-3	6.61E-5	0.144	1.61E-3	0.336	7.44E-4	0.287	8.83E-3
28.00	2.28E-3	5.59E-5	0.137	1.57E-3	0.312	6.78E-4	0.265	9.32E-3
30.00	1.93E-3	4.03E-5	0.130	1.30E-3	0.293	5.19E-4	0.256	8.00E-3
32.00	1.65E-3	3.35E-5	0.124	1.25E-3	0.276	6.60E-4	0.251	8.58E-3
34.00	1.37E-3	3.22E-5	0.118	1.41E-3	0.260	6.62E-4	0.249	9.74E-3
36.00	1.18E-3	2.67E-5	0.114	1.29E-3	0.246	5.58E-4	0.242	9.25E-3
38.00	1.02E-3	2.48E-5	0.111	1.32E-3	0.234	5.19E-4	0.244	9.68E-3
40.00	8.67E-4	2.11E-5	0.109	1.28E-3	0.221	4.56E-4	0.219	9.98E-3
42.00	7.38E-4	1.29E-5	0.107	8.74E-4	0.209	2.82E-4	0.204	7.14E-3
44.00	6.27E-4	3.10E-5	0.105	2.33E-3	0.202	6.59E-4	0.219	1.87E-2
46.00	4.69E-4	3.04E-5	0.099	3.00E-3	0.192	7.03E-4	0.225	2.22E-2
48.00	3.59E-4	1.40E-5	0.098	1.74E-3	0.184	3.13E-4	0.207	1.25E-2
50.00	2.50E-4	1.47E-5	0.097	2.40E-3	0.176	3.06E-4	0.217	1.56E-2

Table S12. The fitting parameters of ac data for **5%Dy@1-Y** under zero dc field to the Generalised Debye Model using the CC-FIT2 program.

T	τ	τ^{err}	χ_S	χ_S^{err}	χ_T	χ_T^{err}	α	α^{err}
(K)	(s)		(cm^3 mol^{-1})		(cm^3 mol^{-1})			
2.00	1.99E-3	3.44E-5	0.457	1.72E-2	3.949	7.29E-3	0.503	3.41E-3
2.50	2.10E-3	4.97E-5	0.470	2.12E-2	3.237	1.30E-2	0.450	6.66E-3
3.00	1.49E-3	4.21E-5	0.547	2.28E-2	2.624	1.16E-2	0.295	1.08E-2
3.50	8.07E-4	1.70E-5	0.482	1.78E-2	2.215	6.53E-3	0.191	9.28E-3
4.00	4.30E-4	7.70E-6	0.404	1.59E-2	1.934	3.83E-3	0.138	7.73E-3
4.50	2.38E-4	5.09E-6	0.353	1.91E-2	1.705	2.69E-3	0.095	8.04E-3
5.00	1.33E-4	6.54E-6	0.278	4.18E-2	1.538	2.82E-3	0.088	1.30E-2
5.50	6.91E-5	8.34E-6	0.119	1.06E-1	1.397	2.67E-3	0.092	1.95E-2
6.00	4.06E-5	6.26E-6	0.000	1.46E-1	1.277	1.45E-3	0.070	1.67E-2

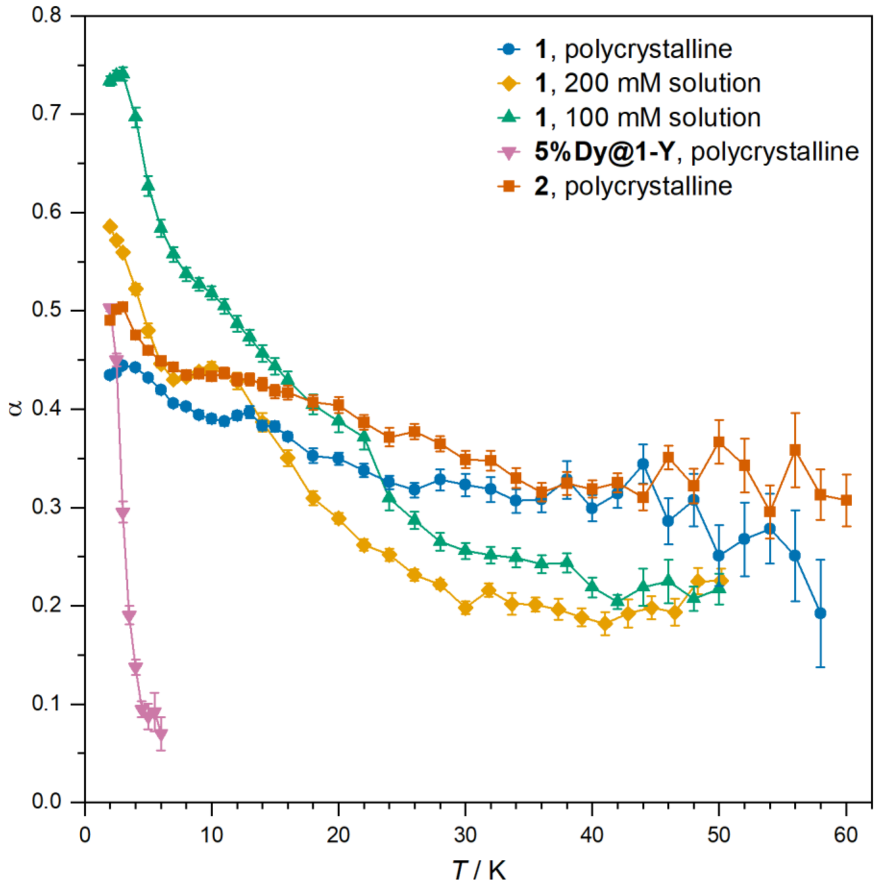

Figure S66. Temperature dependence of the parameter α between 2 and 60 K in zero-dc field deduced as extracted from the generalised Debye fit of the frequency dependence of the real (χ') and imaginary (χ'') components of the ac susceptibility of polycrystalline **1**, **2** and **5%Dy@1-Y**, and solution state **1**.

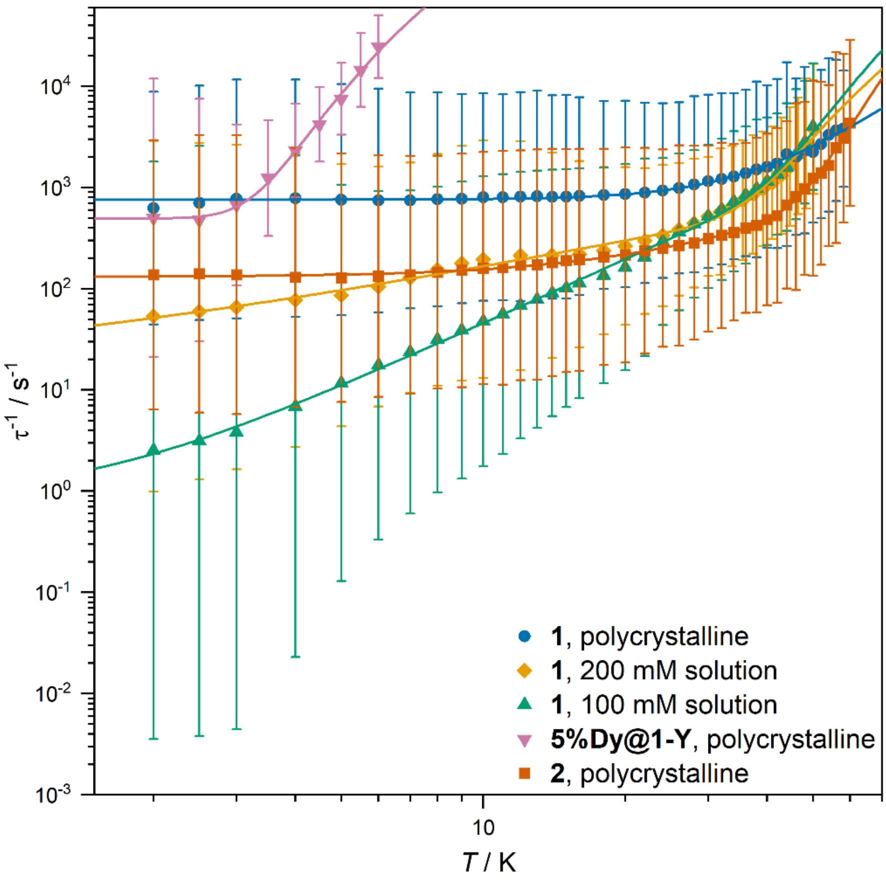

Figure S67. Temperature dependence of the relaxation times for polycrystalline **1**, **2** and **5%Dy@1-Y**, and solution state **1** under a zero applied field with fits to the relaxation profile as given in Table 2. Error bars represent 1 ESD in the distribution of experimental relaxation rates.

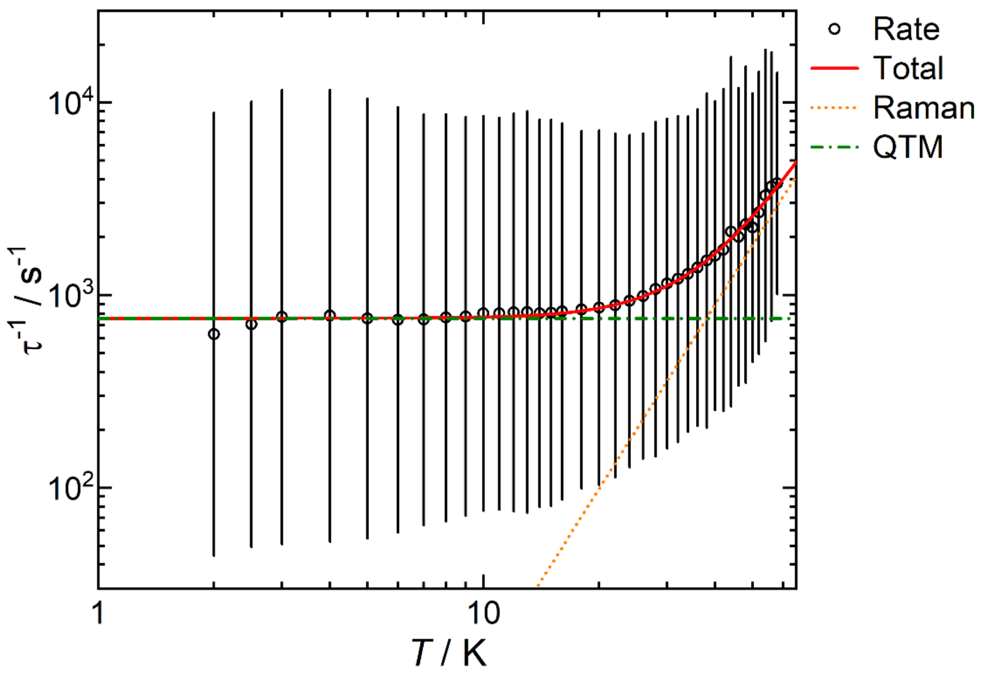

Figure S68. Temperature dependence of the relaxation times for **1** under a zero applied field. The error bars represent 1 ESD in the distribution of experimental rates but have not been used when fitting the temperature-dependence. The solid red line is the best fit to a combination of Raman (orange dot) and QTM (green dash-dot) processes [$\tau^{-1} = 10^{R}T^{n} + 10^{Q}$] with the following parameters: R = -2.1 ± 0.2 $\log_{10}$[s^{-1} K^{-n}], n = 3.17 ± 0.13, Q = -2.880 ± 0.006 $\log_{10}$[s].

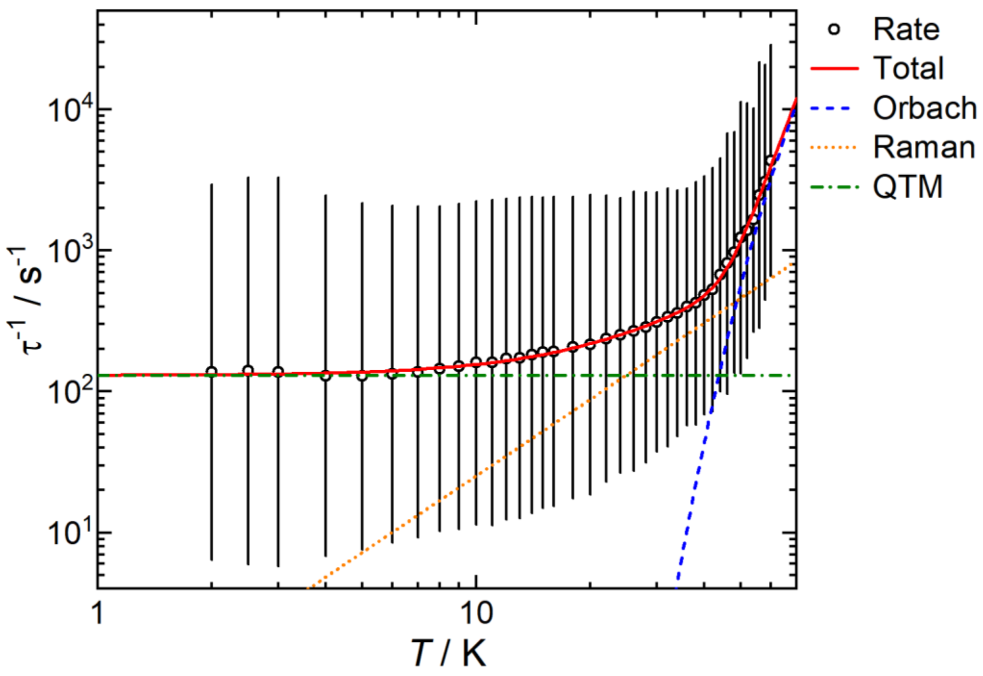

Figure S69. Temperature dependence of the relaxation times for **2** under a zero applied field. The error bars represent 1 ESD in the distribution of experimental rates but have not been used when fitting the temperature-dependence. The solid red line is the best fits to a combination of Orbach (blue dash), Raman (orange dot) and QTM (green dash-dot) processes [$\tau^{-1} = 10^{-A}\exp(U_{eff}/T) + 10^{R}T^{n} + 10^{-Q}$] with the following parameters: A = -7.2 ± 0.2 $\log_{10}$[s], U_{eff} = 510 ± 20 K, R = -0.4 ± 0.2 $\log_{10}$[s^{-1} K^{-n}], n = 1.80 ± 0.11, Q = -2.116 ± 0.010 $\log_{10}$[s].

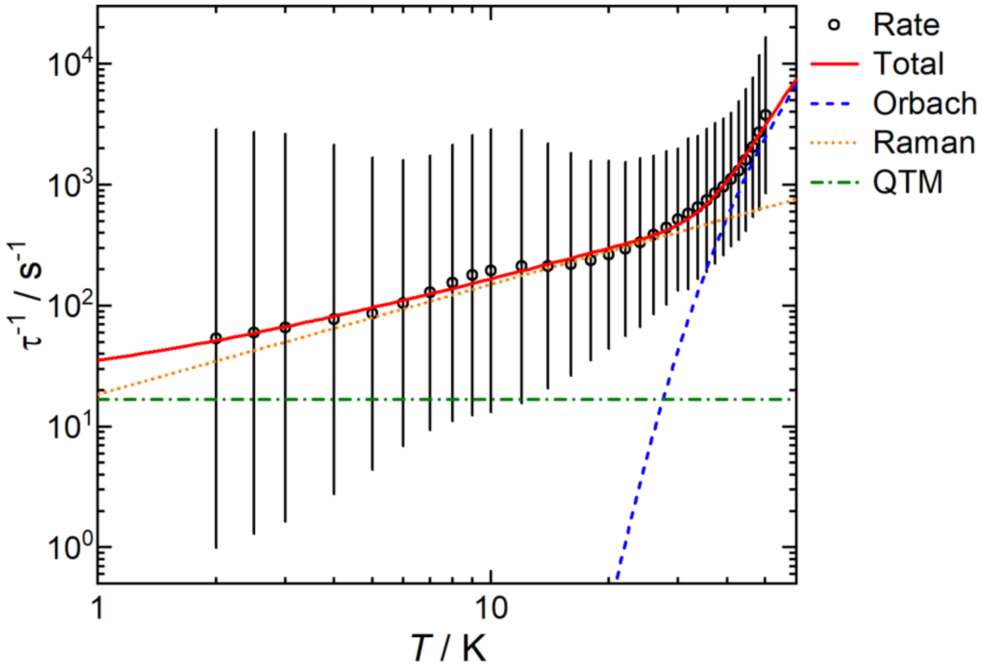

Figure S70. Temperature dependence of the relaxation times for a 200 mM frozen solution of **1** in 9 : 1 toluene : hexane under a zero applied field. The error bars represent 1 ESD in the distribution of experimental rates but have not been used when fitting the temperature-dependence. The solid red line is the best fits to a combination of Orbach (blue dash), Raman (orange dot) and QTM (green dash-dot) processes [$\tau^{-1} = 10^{-A}\exp(U_{eff}/T) + 10^{R}T^{n} + 10^{-Q}$] with the following parameters: A = -6.0 ± 0.3 $\log_{10}$[s], U_{eff} = 300 ± 30 K, R = 1.3 ± 0.2 $\log_{10}$[s^{-1} K^{-n}], n = 0.91 ± 0.11, Q = -1.2 ± 0.3 $\log_{10}$[s].

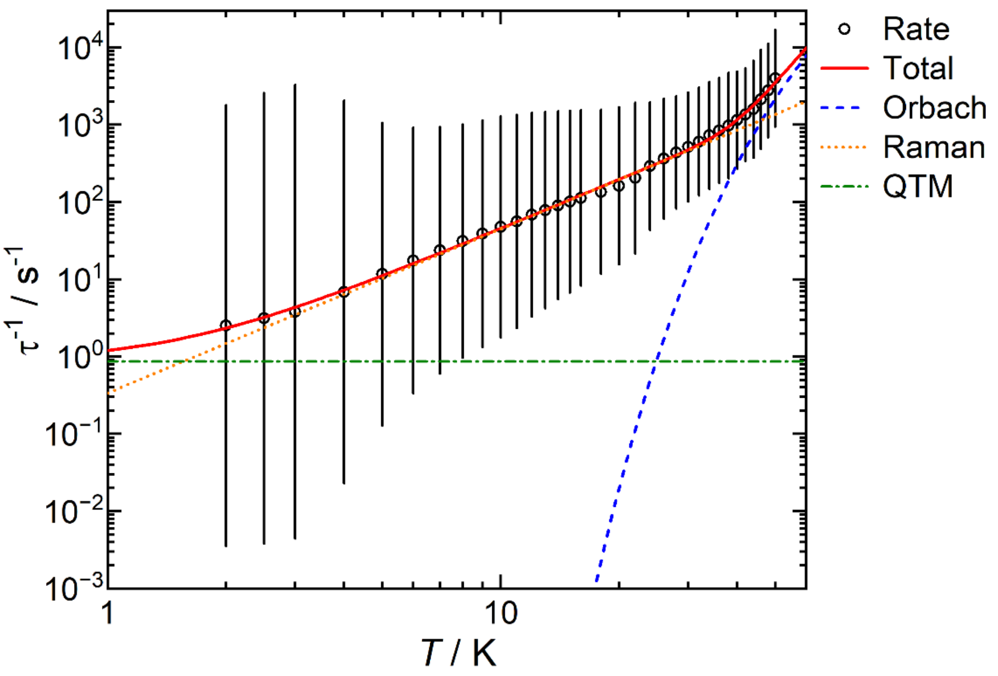

Figure S71**.** Temperature dependence of the relaxation times for a 100 mM frozen solution of **1** in 9 : 1 toluene : hexane under a zero applied field. The error bars represent 1 ESD in the distribution of experimental rates but have not been used when fitting the temperature-dependence. The solid red line is the best fits to a combination of Orbach (blue dash), Raman (orange dot) and QTM (green dash-dot) processes [$\tau^{-1} = 10^{-A}\exp(U_{eff}/T) + 10^{R}T^{n} + 10^{-Q}$] with the following parameters: A = -6.7 ± 0.5 $\log_{10}$[s], U_{eff} = 390 ± 50 K, R = -0.47 ± 0.05 $\log_{10}$[s^{-1} K^{-n}], n = 2.12 ± 0.05, Q = 0.06 ± 0.13 $\log_{10}$[s].

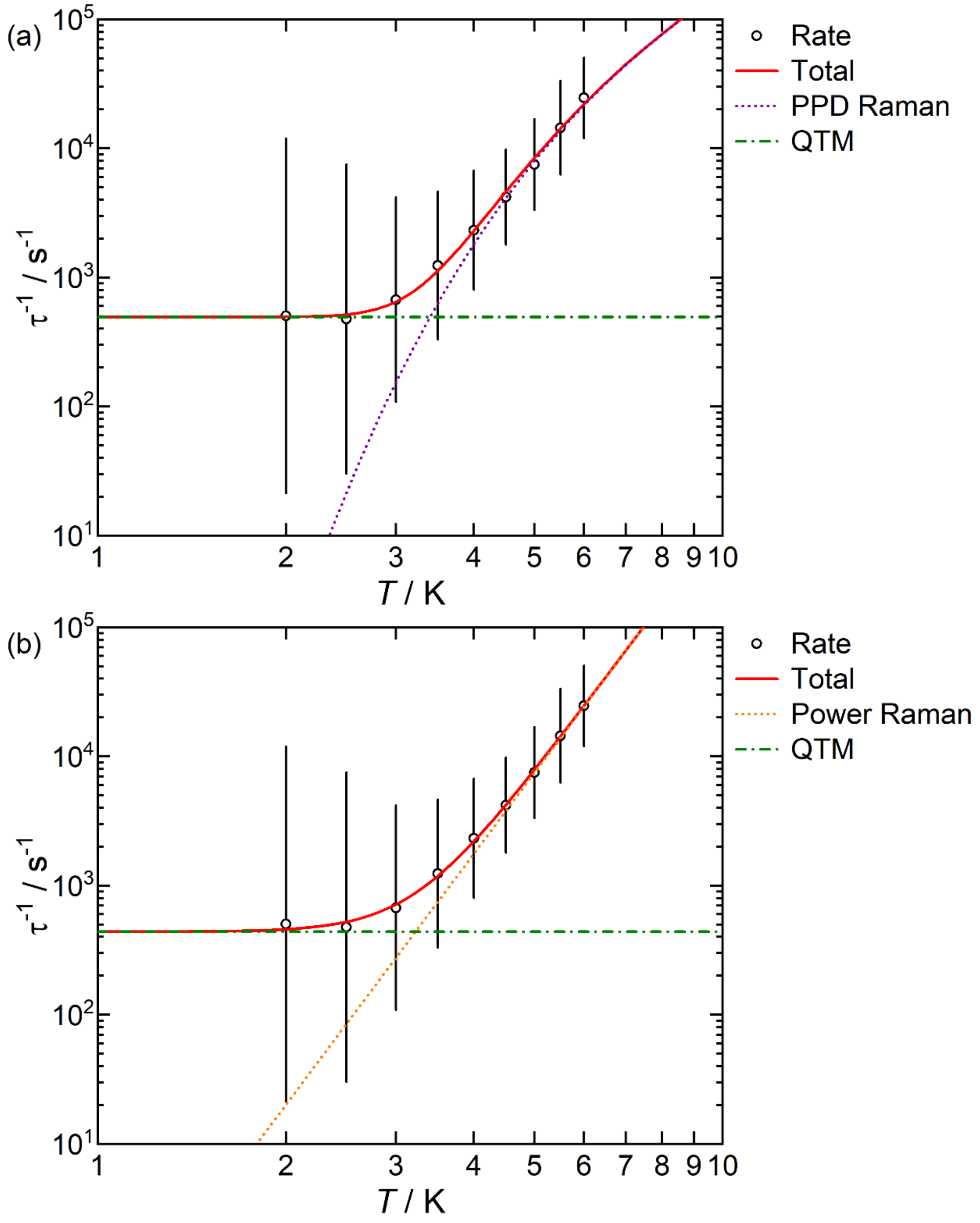

Figure S72. Temperature dependence of the relaxation times for **5%Dy@1-Y** under a zero applied field showing a fit (solid red line). (a) Fit to a combination of phonon pair-driven Raman (purple dot) and QTM (green dash-dot) processes [$\tau^{-1} = 10^{M}\exp(\omega/T)/(\exp(\omega/T)-1)^{2} + 10^{Q}$] with the following parameters: M = 6.47 ± 0.12 $\log_{10}$[s^{-1}], ω= 29.6 ± 1.3 K, Q = -2.69 ± 0.03 $\log_{10}$[s], equivalent to $P = 10^{M} = 2.9^{+0.9}_{-0.7} \times 10^{6}$ s^{-1} and $\tau_{QTM} = 10^{Q} = 2.02^{+0.14}_{-0.13} \times 10^{-3}$ s. (b) Fit to a combination of power-law

Raman (orange dot) and QTM (green dash-dot) processes [$\tau^{-1} = 10^{R}T^{n} + 10^{Q}$] with the following parameters: R = -0.64 ± 0.14 $\log_{10}$[s^{-1} K^{-n}], n = 6.5 ± 0.2, Q = -2.64 ± 0.03 $\log_{10}$[s], equivalent to $C = 10^{R} = 0.23^{+0.09}_{-0.06}$ s^{-1} K^{-n} and $\tau_{\mathrm{QTM}} = 10^{Q} = 2.28^{+0.14}_{-0.13} \times 10^{-3}$ s. The error bars represent 1 ESD in the distribution of experimental rates but have not been used when fitting the temperature-dependence.

9. References

1 W. Huang, J. J. Le Roy, S. I. Khan, L. Ungur, M. Murugesu and P. L. Diaconescu, *Inorg. Chem.*, 2015, **54**, 2374–2382.

2 K. Searles, B. L. Tran, M. Pink, C.-H. Chen and D. J. Mindiola, *Inorg. Chem.*, 2013, **52**, 11126–11135.

3 S. K. Sur, *J. Magn. Reson.*, 1989, **82**, 169–173.

4 D. F. Evans, *J. Chem. Soc.*, 1959, 2003.

5 CrysAlis PRO. Agilent Technologies Ltd, Yarnton, Oxfordshire, England. 2014.

6 G. M. Sheldrick, *Acta Crystallogr. A Found. Crystallogr.*, 2008, **64**, 112–122.

7 G. M. Sheldrick, *Acta Crystallogr. C Struct. Chem.*, 2015, **71**, 3–8.

8 O. V. Dolomanov, L. J. Bourhis, R. J. Gildea, J. A. K. Howard and H. Puschmann, *J. Appl. Crystallogr.*, 2009, **42**, 339–341.

9 L. J. Farrugia, *J. Appl. Crystallogr.*, 2012, **45**, 849–854.

10 Persistence of Vision Raytracer (version 3.6) Persistence of Vision Pty. Ltd., Williamstown, Victoria, Australia 2004.

11 S. Alvarez, P. Alemany, D. Casanova, J. Cirera, M. Llunell and D. Avnir, *Cord. chem. rev.*, 2005, **249**, 1693–1708.

12 I. Fdez. Galván, M. Vacher, A. Alavi, C. Angeli, F. Aquilante, J. Autschbach, J. J. Bao, S. I. Bokarev, N. A. Bogdanov, R. K. Carlson, L. F. Chibotaru, J. Creutzberg, N. Dattani, M. G. Delcey, S. S. Dong, A. Dreuw, L. Freitag, L. M. Frutos, L. Gagliardi, F. Gendron, A. Giussani, L. González, G. Grell, M. Guo, C. E. Hoyer, M. Johansson, S. Keller, S. Knecht, G. Kovačević, E. Källman, G. Li Manni, M. Lundberg, Y. Ma, S. Mai, J. P. Malhado, P. Å. Malmqvist, P. Marquetand, S. A. Mewes, J. Norell, M. Olivucci, M. Oppel, Q. M. Phung, K. Pierloot, F. Plasser, M. Reiher, A. M. Sand, I. Schapiro, P. Sharma, C. J. Stein, L. K. Sørensen, D. G. Truhlar, M. Ugandi, L. Ungur, A. Valentini, S. Vancoillie, V. Veryazov, O. Weser, T. A. Wesołowski, P.-O. Widmark, S. Wouters, A. Zech, J. P. Zobel and R. Lindh, *J. Chem. Theory Comput.*, 2019, **15**, 5925–5964.

13 L. Ungur and L. F. Chibotaru, *Chem. Eur. J.*, 2017, **23**, 3708–3718.

14 B. O. Roos, R. Lindh, P.-Å. Malmqvist, V. Veryazov and P.-O. Widmark, *J. Phys. Chem. A*, 2004, **108**, 2851–2858.

15 B. O. Roos, R. Lindh, P.-Å. Malmqvist, V. Veryazov and P.-O. Widmark, *J. Phys. Chem. A*, 2005, **109**, 6575–6579.

16 S. Stoll and A. Schweiger, *J. Magn. Reson.*, 2006, **178**, 42–55.

17 A. K. Hassan, L. A. Pardi, J. Krzystek, A. Sienkiewicz, P. Goy, M. Rohrer and L.-C. Brunel, *J. Magn. Reson.*, 2000, **142**, 300–312.

18 D. Reta and N. F. Chilton, *Phys. Chem. Chem. Phys.*, 2019, **21**, 23567–23575.

19 W. J. A. Blackmore, G. K. Gransbury, P. Evans, J. G. C. Kragskow, D. P. Mills and N. F. Chilton, *Phys. Chem. Chem. Phys.*, 2023, **25**, 16735–16744.
